# Environment and Pollen Diversity Differentially Affect the Gut Microbiomes of Introduced Honeybees and Bumblebees

**DOI:** 10.1111/eva.70234

**Published:** 2026-04-13

**Authors:** Sabrina Haque, Fleur Ponton, Andrew P. Allen, Hasinika K. A. H. Gamage, Francisco Encinas‐Viso, Ian T. Paulsen, Rachael Y. Dudaniec

**Affiliations:** ^1^ School of Natural Sciences Macquarie University Sydney New South Wales Australia; ^2^ Pollinator Futures Research Centre Macquarie University Sydney New South Wales Australia; ^3^ ARC Training Centre for Facilitated Advancement of Australia's Bioactives Macquarie University Sydney New South Wales Australia; ^4^ Centre for Australian National Biodiversity Research CSIRO Black Mountain Australian Capital Territory Australia; ^5^ ARC Centre of Excellence in Synthetic Biology Macquarie University Sydney New South Wales Australia

**Keywords:** *Apis mellifera*, bee, *Bombus terrestris*, environment, gut microbiome, invasive species, island, pollen, Tasmania

## Abstract

Invasive species may exhibit shifts in their gut microbiome in response to novel environments and diet, but this may differ across host species and their time since colonisation. We investigate if site environmental variables and foraged pollen resources differentially shape the gut microbiomes of two bee species with contrasting introduction histories: The European honeybee, *Apis mellifera* (introduced 1831), and the recently invasive bumblebee, *Bombus terrestris* (invaded 1992). Using landscape‐scale metabarcoding across the island state of Tasmania in Australia, we characterised gut bacteria (16S rRNA) and corbicular pollen diversity (ITS2) for each species. Gut bacterial composition was significantly associated with mean annual temperature for *A. mellifera* and with mean annual precipitation and percentage of pasture for *B. terrestris* . In *B. terrestris* , the core and facultative gut microbial diversity and richness showed associations with precipitation, foraged pollen diversity, wind velocity and temperature. Foraged pollen diversity of native plants more strongly predicted the facultative gut microbiome across species. Overall, the gut microbiome of *B. terrestris* showed a stronger response to abiotic and biotic predictors compared to *A. mellifera* . Our findings advance understanding of how environmental and dietary factors shape pollinator gut microbiomes at landscape scales, with implications for pollinator health and survival.

## 1 Introduction

The gut microbiome plays a critical role in host health (Clemente et al. 2012) and it may shift in composition and diversity in response to environmental or dietary changes (Falony et al. 2016; Rothschild et al. 2018), such as those encountered by invasive species during colonisation (Dragičević et al. 2021; Zhu et al. 2021). The mechanisms by which such shifts in the gut microbiome occur, and the effects of those shifts on the health and adaptability of hosts is of interest for understanding and managing biological invasions (Escalas et al. 2022; Martignoni and Kolodny 2024). Invasive populations of social Hymenoptera (e.g., bees, wasps, and ants) represent important models for assessing how the gut microbiome influences invasion dynamics due to their global ubiquity and their ecological and economic relevance (Manfredini et al. 2019; Ghisbain et al. 2021). Bees are essential pollinators and common invaders worldwide (Aizen et al. 2020), and they host a conserved core and an environmentally responsive facultative gut microbiome (Engel et al. 2016). Determining how the gut microbiomes of bees respond to changes in climate and dietary resources may therefore be important for understanding how some bee species have proven to be successful invaders.

The European honeybee ( *Apis mellifera* ), likely originating in Asia or Africa, spread through Europe and is now found on every continent except Antarctica (Han et al. 2012). The gut microbiome of *A. mellifera* includes bacteria restricted to the bee gut or hive (Martinson et al. 2011; Anderson et al. 2013), which are transmitted via nestmate contact (Moran et al. 2012; Powell et al. 2014; Kwong and Moran 2016). The buff‐tailed bumblebee, *Bombus terrestris* , native to Europe, is globally invasive and present in regions including South America, New Zealand, Tasmania, and Japan (Aizen et al. 2020). Despite diverging from honeybees around 80 million years ago, bumblebees share most core gut bacterial genera with honeybees, underscoring the functional significance of these genera (Lim et al. 2015; Kwong et al. 2017).

The core gut microbiomes of *A. mellifera* and *B. terrestris* differ substantially and are largely shaped by social behaviour. Honeybees acquire microbes via trophallaxis and hive contact (Kwong et al. 2017; Bulson et al. 2021), while bumblebee workers partially inherit their microbiomes from the founding queen (Hammer et al. 2021; Su et al. 2021). These species share five core gut bacterial genera: *Snodgrassella*, *Gilliamella*, *Bifidobacterium*, *Bombilactobacillus*, and *Lactobacillus* Firm‐5 (Kwong et al. 2017; Raymann and Moran 2018). *B. terrestris* is also found to host *Schmidhempelia* and *Bombiscardovia* (Hammer et al. 2021). In contrast, low‐abundance facultative taxa (~1%–7%) such as *Apibacter*, *Bartonella*, *Bombella*, *Acetobacter*, and *Frischella* are likely acquired from floral resources (Kwong and Moran 2016; Callegari et al. 2021) and differ more markedly between *Apis* and *Bombus* across habitats (Amiri et al. 2023). Identifying which facultative taxa introduced bees acquire may reveal how local floral diversity and climate shape gut microbiomes in novel ecosystems.

Invasive bees can modify local plant‐pollinator networks by altering floral abundance and composition via their pollination behaviour (Feinsinger et al. 1987; Morales and Aizen 2002). Introduced bees, including *A. mellifera* and *B. terrestris* , often prefer exotic plants and may act as their main pollinators (Goulson 2003; Hanley and Goulson 2003; Dafni et al. 2010). Honeybees forage opportunistically on both native and non‐native plants (Stanley et al. 2020), and pollen nutritional content can vary across plant species (Roulston and Cane 2000). Such dietary differences may affect bee health, as polyfloral pollen has been shown to shift gut microbial composition and modulate immune function in honeybees (Braglia et al. 2025). In some areas, *A. mellifera* has reduced native plant pollination while boosting that of invasive species (Barthell et al. 2001; Morales and Aizen 2002). Thus, determining the plant taxa that bees forage on within introduced ranges can help to understand their impacts on ecological processes and ecosystem services.

Bee gut microbiomes are heavily influenced by diet. Nectar provides carbohydrates, while pollen offers key nutrients that support gut bacteria (Wright et al. 2018; Zheng et al. 2019). Pollen intake can boost bacterial abundance in the hindgut (Ricigliano et al. 2017; Kešnerová et al. 2020), whereas poor or pollen‐free diets can reduce beneficial microbes (Ricigliano and Anderson 2020; Luo et al. 2024). Low‐nutrient pollen, such as from *Eucalyptus*, can deplete *Lactobacillus* and *Bifidobacterium*, while increasing *Bartonella apis* and vulnerability to *Nosema ceranae* in honeybees (Castelli et al. 2020). Similarly, protein substitutes used in honeybee hives may lower microbial diversity and raise pathogen risk (Powell et al. 2023). Characterising foraged pollen diversity can therefore help to clarify how diet shapes and supports bee microbiomes and their persistence in new environments.

The European honeybee ( *A. mellifera* ) was introduced to mainland Australia in 1822 and to the island state of Tasmania in 1831 for honey and pollination and is now a widespread introduced species (Oldroyd et al. 1995). Although effective pollinators of some native plants, honeybees can reduce seed set in species like *Melastoma affine* (Gross and Mackay 1998). In contrast, *B. terrestris* was found to have invaded Tasmania in 1992 and rapidly spread across the island (Semmens et al. 1993), but it remains absent from the mainland. In Tasmania, *B. terrestris* is considered invasive and competes with native bees and birds (Hingston and McQuillan 1999), displaces native pollinators (Dafni and Shmida 1996; Matsumura et al. 2004), and may promote invasive weeds (Hingston 2005), lowering pollination efficiency for native plants (Hingston et al. 2004; A. B. Hingston 2006, 2007).

Here, we investigate how gut microbiomes of a long‐established pollinator, *A. mellifera* (since 1831) and a recent invader, *B. terrestris* (since 1992), vary with foraged pollen diversity and local environmental conditions using landscape‐scale metabarcoding of gut bacteria (16S rRNA) and corbicular pollen (ITS2) across Tasmania, Australia. Due to inherent differences between *A. mellifera* and *B. terrestris* (e.g., time since colonisation, colony size, diet, habitat), we tested whether: (i) core and facultative gut microbiomes differ between bee species in relation to pollen type (native versus introduced) and environmental variables, with the prediction that the more recently invaded *B. terrestris* will show stronger associations compared to *A. mellifera*. Our findings highlight how diet and environment interact to shape the gut microbiome and success of invasive species in novel landscapes.

## 2 Materials and Methods

### 2.1 Study Design and Bee Collections

**FIGURE 1:**
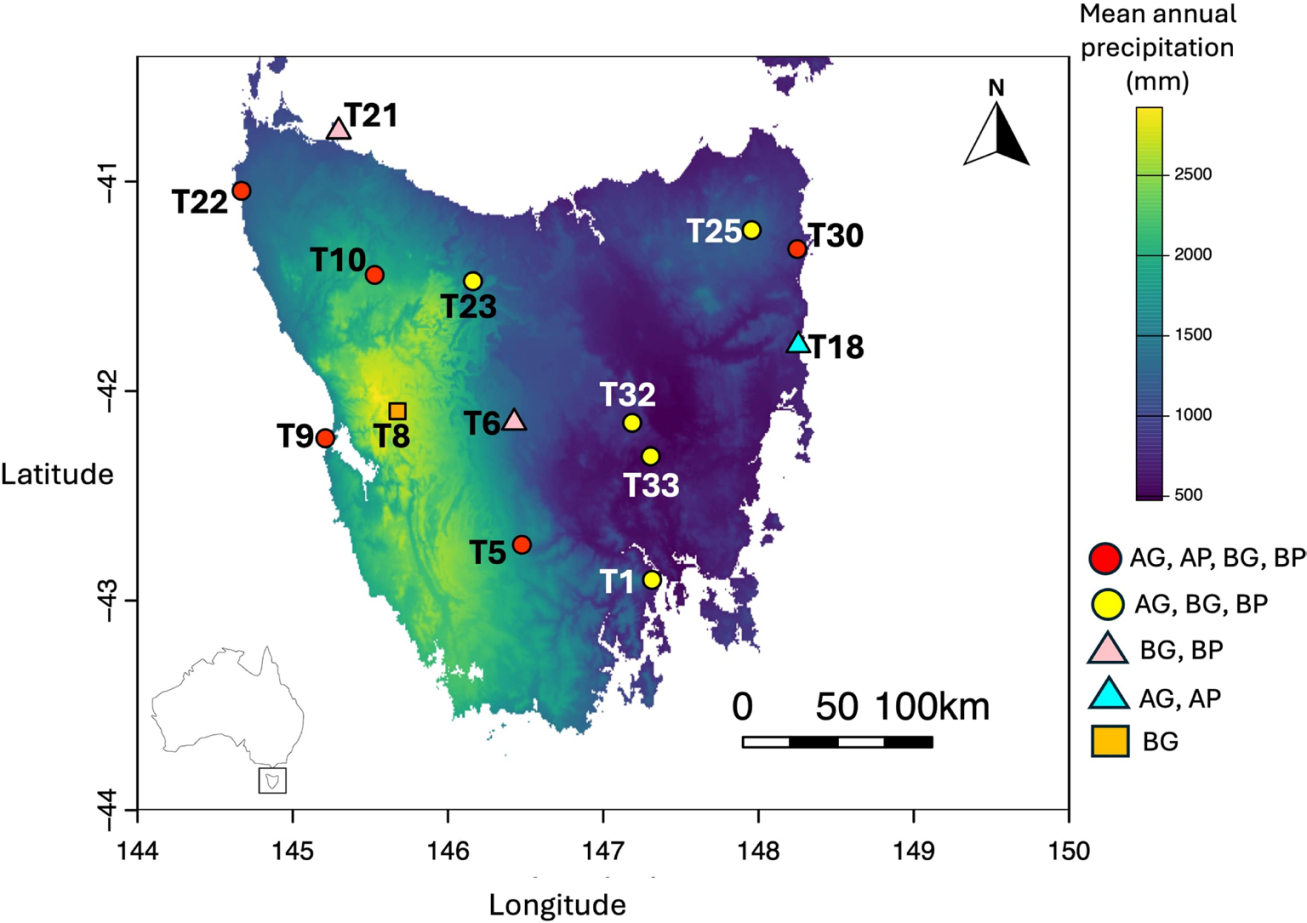
Sampling sites for *A. mellifera* (*n* = 11) and *B. terrestris* (*n* = 13) shown across Tasmania, Australia (inset). The sites are overlaid on a map of mean annual precipitation (mm). Legend shows sites included for the: AG, *A. mellifera* gut microbiome study; AP, *A. mellifera* pollen study; BG, *B. terrestris* gut microbiome study; BP, *B. terrestris* pollen study. Refer to Table 1 for site names.

Female worker *A. mellifera* and *B. terrestris* were collected during peak summer activity (24 January–1 February 2023) from 14 sites across Tasmania (Table 1; Figure 1). Sampling was conducted using a free‐search or opportunistic approach consistent with a previous study investigating bee gut microbiomes and pollen‐associated floral communities (Haque et al. 2025). Sites were selected based on species occurrence records (Hingston et al. 2002; Hingston 2006), prior ecological and microbiome studies (Kardum Hjort et al. 2023, 2024; Haque et al. 2025), and the presence of flowering resources during the sampling period. Within each site, bees were collected from flowering plants in open areas spanning urban, rural, and residential locations during peak daytime activity. Bees that were actively engaged in foraging were targeted. Although individual foraging behaviour was not directly observed for every captured bee, this approach maximised sampling of foraging female workers.

**TABLE 1:** Sites (*N* = 14) sampled for *A. mellifera* and *B. terrestris* across Tasmania.

Site ID	Site name	N Am	N Bt	AT	AR	PP	WV
T1	Hobart	9	7	11.48	752	0	5.80
T5	Southwest	10	6	8.88	1632	6.06	5.10
T6	Bronte Park	N/A	8	8.43	1242	0	3.90
T8	Franklin Gordon	N/A	6	10.63	2605	0	5.60
T9	Macquarie Heads	5	7	11.74	1571	2.85	6.70
T10	Tikkawoppa Waratah	10	8	8.88	2046	4.84	4.90
T18	Douglas River	10	N/A	12.66	687	0	5.10
T21	Stanley	N/A	8	13.03	1038	48.8	6.40
T22	Arthur River	7	7	12.59	1166	0	6.70
T23	Cethana	9	7	9.82	1478	5.08	4.60
T25	Weldborough	10	7	9.30	1177	11.5	4.30
T30	St Helens	10	5	13.15	766	30	5.30
T32	Interlaken	10	4	7.96	804	0	4.00
T33	Oatlands	10	8	10.64	506	71	3.90

*Note:* Refer to Figure 1 for corresponding site locations.

Abbreviations: AR, mean annual precipitation (mm); AT, mean annual temperature (°C); N/A, not applicable; N Am, number of honeybees ( *A. mellifera* ) sampled for gut microbiome and pollen; N Bt, number of bumblebees ( *B. terrestris* ) sampled for gut microbiome and pollen; PP, percentage of pasture (%); WV, average velocity of summer wind (m/s).

Sampling was restricted to female worker bees, the primary foraging caste responsible for pollen and nectar collection. In contrast, male bees do not forage and lack key anatomical features associated with resource collection, such as pollen baskets and wax glands. For *B. terrestris* , sex was determined by counting the number of antennal segments under a dissecting microscope (males: 11 segments, females: 10 segments after the pedicel). Although bees were sampled from 14 sites, successful gut microbiome collection was achieved at 11 sites for *A. mellifera* and 13 sites for *B. terrestris* due to low availability of female workers at a site and sample quality following gut dissection and DNA extraction. Comparative microbiome analyses between species were performed on the overlapping sites (*N* = 10, Figure 1).

### 2.2 Selection and Correlation of Environmental Variables

Environmental data for the 14 sites were obtained (Table 1) as described in Kardum Hjort et al. (2023, 2024) and Haque et al. (2025), which analysed sites that largely overlapped with our present study. Here, we retain a subset of four variables that were previously informative about gut microbiome, morphological and genetic patterns in Tasmanian bees (Kardum Hjort et al. 2023, 2024; Haque et al. 2025). Mean annual temperature, mean annual precipitation, and average summer wind velocity were sourced from WorldClim v2.1 (Fick and Hijmans 2017), and pasture percentage from the Dynamic Land Cover Dataset v2.1 within a 1 km radius of each site (Lymburner et al. 2015), Our previous studies evaluated a broader set of climatic and land‐use variables, including precipitation seasonality, vegetation height and percentage of urban area, but these variables were excluded due to high correlation (Kardum Hjort et al. 2023, 2024; Haque et al. 2025). Pearson correlation coefficients were calculated in R version 4.4.1 (R Core Team 2024) to assess collinearity among the selected environmental variables for both species, with pairwise correlations ≥ 0.70 considered indicative of strong correlation (Text S1 and Table S1).

### 2.3 Gut Microbiome DNA Extraction and 16S rRNA Sequencing

Each bee was rinsed in 70% ethanol and 1X PBS before dissecting the mid and hindguts of *A. mellifera* (*n* = 100; 5–10 bees per site; 11 sites) and *B. terrestris* (*n* = 88; 4–8 bees per site; 13 sites) under a stereo microscope (Table 1). DNA was extracted using a modified DNeasy Blood & Tissue protocol (Qiagen) and quantified with a Qubit dsDNA High‐Sensitivity kit (Text S2). The V4 region of the 16S rRNA gene was amplified and sequenced (2 × 250 bp paired‐end) on an Illumina MiSeq at the Ramaciotti Centre, UNSW, Sydney, Australia (Text S3). Reads were processed in QIIME2 v2024.10 (Bolyen et al. 2019), denoised with Deblur (Amir et al. 2017), and classified using a Naïve Bayes classifier against Silva‐138, with genus‐level validation via *blastn* (*e*‐value ≤ 1 × 10^−30^; identity ≥ 95%; Text S4).

### 2.4 Gut Bacterial Taxonomic Composition and Alpha Diversity

Sequencing of 16S rRNA from *A. mellifera* individuals across 11 sites (*n* = 100) yielded 5,082,524 reads, of which 4,255,076 remained after demultiplexing and quality filtering. For *B. terrestris* individuals from 13 sites (*n* = 88), 3,910,448 reads were generated, with 3,312,408 retained post‐filtering. The total number of reads generated per sample for both species can be found in Tables S2 and S3. For plotting core and facultative gut bacterial relative abundances, only those contributing to ≥ 1% relative abundance across all sites were retained and visualised. This 1% threshold was applied to reduce noise from low‐abundance bacterial ASVs and highlight the dominant taxa contributing to the gut bacterial composition; however, all ASVs were retained for analysis. The core bacterial taxa—*Snodgrassella, Gilliamella, Bifidobacterium, Bombilactobacillus*, and *Lactobacillus* Firm‐5—were defined based on their consistent detection across many previous studies of *A. mellifera* and *B. terrestris* (e.g., Hammer et al. 2021; Yang et al. 2024). All other bacterial genera were classified as facultative.

Bray–Curtis dissimilarities in gut bacterial composition were separately calculated for samples of *A. mellifera* (*n* = 100) and *B. terrestris* (*n* = 88) based on relative abundances of amplicon sequence variants (ASVs) and Non‐metric Multi‐Dimensional Scaling (NMDS) ordination was applied using the *vegan* R package v2.6‐4 (Oksanen et al. 2024). Correlations of NMDS scores on axes 1 and 2 with environmental vectors (temperature, precipitation, % pasture, and wind velocity) were represented as arrows in the bivariate NMDS plots. NMDS scores were averaged for plotting to visualise patterns at the site level using centroids and standard error bars. Pairwise permutation multivariate analysis of variance (PERMANOVA) was performed on sample‐level dissimilarities using the *pairwiseAdonis* R package v0.4.1 (Martinez Arbizu 2020) to test for site‐level differences in community composition. Bonferroni‐adjusted *p*‐values were used to correct for multiple comparisons. Group dispersion was assessed with *betadisper* and *permutest* in *vegan*, confirming no significant heterogeneity (*p* > 0.05), and validating the PERMANOVA outputs.

Principal Coordinates Analysis (PCoA) was performed using Jaccard dissimilarities based on presence–absence–transformed ASV data to assess patterns in community membership independent of abundance, with percentage variance reported for each axis. Environmental variables were fitted to the Jaccard PCoA ordination using permutation tests (envfit; 999 permutations), and only significant vectors were visualised. PCoA was subsequently conducted on Bray–Curtis dissimilarities calculated from ASV count data to visualise abundance‐weighted community structure, with percentage variance calculated from positive eigenvalues only. Environmental variables were similarly fitted to the Bray–Curtis PCoA ordination (envfit; 999 permutations), and only significant vectors were displayed.

Alpha diversity of gut bacteria was assessed using Chao1 richness and Shannon diversity indices, calculated with the *phyloseq* R package v1.44.0 (McMurdie and Holmes 2013). To evaluate pairwise differences in alpha diversity among sites, a one‐way analysis of variance (ANOVA) was performed for both Chao1 and Shannon indices, using site as the factor followed by Tukey's post hoc tests.

### 2.5 Pollen ITS2 Sequencing and Taxonomic Classification

Corbicular pollen was removed from each bee, pooled per site and extracted as outlined in Text S5. PCR amplification of the ITS2 region was performed using S2F/S3R primers (Chen et al. 2010) and amplicons were purified and sequenced (2 × 250 bp, Illumina MiSeq; Text S4) at the Ramaciotti Centre, UNSW, Sydney. Primer sequences were trimmed with *cutadapt* (Martin 2011) and reads processed in *DADA2* v1.8 using the ITS pipeline (Callahan et al. 2017). Sequencing read summaries for pollen samples, including reads retained after filtering, denoising, and chimera removal were recorded for both species (Tables S4 and S5). *A. mellifera* samples (*N* = 7 sites) yielded 387,390 reads (with 58,730 retained), and *B. terrestris* samples (*N* = 12 sites) yielded 703,942 reads (with 316,826 retained). ASVs with < 10 reads were excluded for both species. Plant genera were assigned via *blastn* (*e*‐value ≤ 1 × 10^−50^, identity ≥ 90%). Alpha diversity measures (Chao1, Shannon) were calculated using *phyloseq*.

### 2.6 Interactions Between Gut Bacteria, Pollen and the Local Environment

Pollen alpha diversity (Chao1 and Shannon) was separately calculated for three classifications of plant genera: (i) ‘native’ (including endemic) Australian genera, (ii) ‘introduced’ (or invasive) genera in Tasmania, and (iii) a ‘both’ category, including plant genera with both native and introduced species (Key to Tasmanian Vascular Plants 2024; Australian Virtual Herbarium 2024). To examine how gut bacterial diversity responds to pollen and environmental factors, linear mixed‐effects models were fitted using *lmer* (lme4 v1.1.35.5; Bates et al. 2015) and *lmerTest* (v3.1.3; Kuznetsova et al. 2017), with ‘site’ as a random effect. Fixed‐effect significance was tested using Satterthwaite's approximation. Pasture percentages were logit‐transformed to meet normality assumptions. Interaction plots were created using *visreg* v2.7.0 (Brehany and Burchett 2017) and *akima* v0.6.3.4 (Akima and Gebhardt 2022). Linear mixed‐effect models were conducted using both Chao1 richness and Shannon's diversity indices for corbicular pollen and gut bacterial taxa each for *A. mellifera* and *B. terrestris*. Specifically, we tested: (i) how total pollen and environmental variables (temperature, precipitation, pasture, and wind) affect the total gut bacteria, followed by independent tests of how core and facultative bacteria are affected by (ii) native pollen and environmental variables; (iii) introduced pollen and environmental variables; and (iv) ‘both’ pollen and environmental variables.

## 3 Results

### 3.1 Taxonomic Characterisation of Gut Bacteria

**FIGURE 2:**
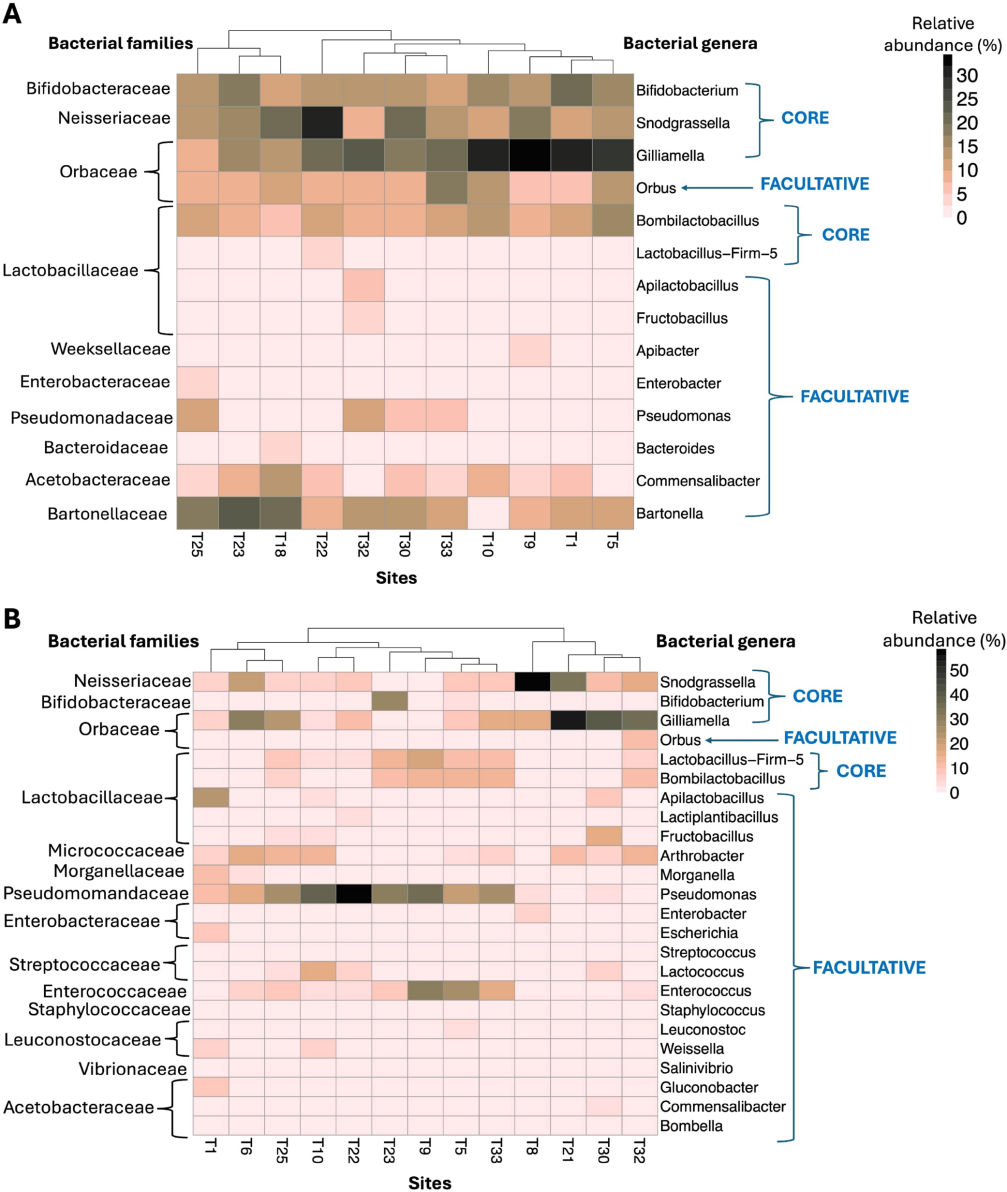
Core and facultative members of (A) *A. mellifera* gut microbiome across 11 sites in Tasmania and (B) *B. terrestris* gut microbiome across 13 sites in Tasmania. The heatmap shows the relative abundance of bacterial genera within each site. The dendrogram at the top depicts the distance in bacterial relative abundances among sites, which is generated via a Euclidean distance matrix.

Ten major (relative abundance > 1%) bacterial families were identified in the gut microbiome of *A. mellifera* and 15 in *B. terrestris* (Figure S1). At the genus level, taxa with > 1% relative abundance were classified as core or facultative genera (Figure 2). Both species shared five core genera: *Snodgrassella*, *Gilliamella*, *Bifidobacterium*, *Lactobacillus* Firm‐5, and *Bombilactobacillus*. Facultative genera were more diverse in *B. terrestris* (*N* = 19) than in *A. mellifera* (*N* = 9), with six genera shared: *Orbus*, *Commensalibacter*, *Apilactobacillus*, *Fructobacillus*, *Enterobacter*, and *Pseudomonas* (Figure 2). Heatmap‐dendrograms based on Euclidean distances showed distinct site‐level clustering of bacterial genera for each species (Figure 2). In *A. mellifera* , one cluster (T25, T23, T18) was enriched in *Bartonella*, while another (T33, T10, T9, T1, T5) was dominated by *Gilliamella* (Figure 2A). In *B. terrestris* , T1 had elevated *Apilactobacillus* (22%), while T10, T22, T23, T9, T5 and T33 formed a cluster with high *Pseudomonas* (20%–56%). A third cluster (T8, T21, T30, T32) showed dominance of *Snodgrassella* and *Gilliamella* (Figure 2B). These patterns were site‐ and species‐specific, but not geographically structured, suggesting environmental or dietary influences.

### 3.2 Gut Bacterial Community Composition and Environmental Correlations

**FIGURE 3:**
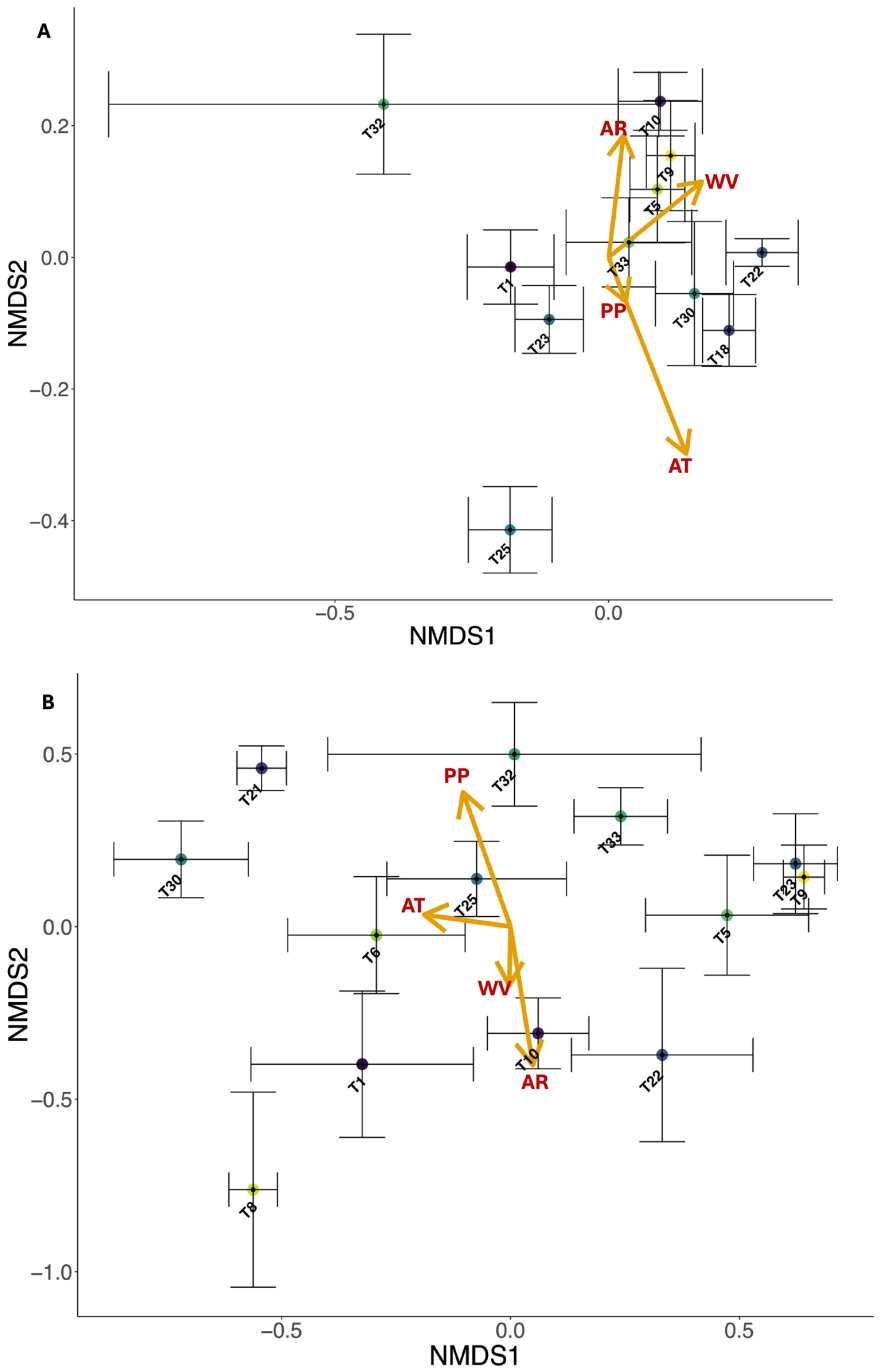
NMDS ordination of gut microbial communities for (A) *A. mellifera* and (B) *B. terrestris* across Tasmania, based on Bray–Curtis dissimilarity of ASV abundance. Sample NMDS scores were averaged at the site level and represented graphically using centroids and standard error bars. Stress for both NMDS plots = 0.2. AR, mean annual precipitation (mm); AT, mean annual temperature (°C); PP, percentage of pasture (%); WV, average summer wind velocity (m/s). Refer to Table S6 for summary of environmental vector correlations (envfit; 999 permutations).

Environmental variable correlations were mainly weak, and all variables were retained (Text S1). For *A. mellifera* , the first NMDS axis (NMDS‐1) of the site‐level ordination, which represented the dominant gradient in gut bacterial composition (Figure 3A and Figure S2A), was significantly associated with mean annual temperature (*p* = 0.01, *r* ^2^ = 0.09; Table S6). In *B. terrestris* , NMDS‐1 (Figure 3B and Figure S2B) was significantly correlated with both mean annual precipitation (*p* = 0.002, *r* ^2^ = 0.16; Table S6) and pasture percentage (*p* = 0.001, *r* ^2^ = 0.16; Table S6). PERMANOVA revealed significant site‐level variation in gut bacterial composition for both bee species. In *A. mellifera* , 41.8% of pairwise comparisons (23/55) were significant (*p* ≤ 0.05), with site T10, which had the second‐highest precipitation, differing in 7 of 11 comparisons (Table S7). *B. terrestris* showed even greater differentiation, with 56.4% of comparisons (44/78) significant, 14.6% more than in *A. mellifera* (Table S8). Notably, site T1 (city of Hobart with the highest urbanisation) differed significantly from all other sites, while T21 (second‐highest mean annual temperature; Table 1) differed from all sites except for T32 (lowest mean annual temperature; Table 1).

Jaccard‐based PCoA ordinations revealed clearer environmental structuring of gut microbiome community membership than Bray–Curtis PCoA for both host species. In *A. mellifera* , the first two Jaccard PCoA axes explained 11.4% and 9.6% of the variance (Figure S3A), with significant associations with mean annual temperature (*R* ^2^ = 0.23, *p* = 0.001; Table S9), mean annual precipitation (*R* ^2^ = 0.14, *p* = 0.001; Table S9), pasture percentage (*R* ^2^ = 0.21, *p* = 0.001; Table S9), and wind velocity (*R* ^2^ = 0.10, *p* = 0.02; Table S9). In *B. terrestris* , Jaccard PCoA accounted for 13.3% and 9.2% of variance along the first two axes (Figure S3C), with precipitation (*R* ^2^ = 0.17, *p* = 0.001; Table S9) and pasture (*R* ^2^ = 0.18, *p* = 0.002; Table S9) emerging as significant environmental correlates, consistent with the NMDS ordination. In contrast, Bray–Curtis PCoA explained a moderate proportion of variance for *A. mellifera* (16.7% and 11.7%) and *B. terrestris* (29% and 14.8%) but showed no significant environmental associations (Table S9; Figure S3B,D).

### 3.3 Alpha Diversity of Gut Bacteria Across Sites

In *A. mellifera* , Chao1 richness was highest at site T30 (Figure S4A) and varied significantly across sites (ANOVA: *F* = 2.44, *p* = 0.01; Table S10). However, only one of 55 pairwise comparisons (1.8%) was significant, between T9 and T25 (Tukey: *p* = 0.03; Table S10), indicating limited site differentiation in gut bacterial richness for *A. mellifera* . Gut bacterial diversity (Shannon's index) in *A. mellifera* was highest at T30 and T32 (Figure S4B), but there was no significant variation across sites (ANOVA: *F* = 1.01, *p* = 0.44; Table S10), and no significant pairwise comparisons (Tukey: all *p* > 0.05; Table S10).

In contrast, *B. terrestris* showed stronger site‐level variation. Chao1 richness differed significantly among sites (ANOVA: *F* = 7.66, *p* = 4.75e^−9^; Table S11), and T8 (which notably had the highest mean annual precipitation; Table 1) exhibiting the highest taxonomic richness (Figure S5A) and differing significantly from all other sites (Tukey: all *p* < 0.001; Table S11). Overall, 12 of 78 pairwise comparisons (15%) were significant, all involving T8. Shannon's diversity of gut bacteria in *B. terrestris* varied marginally among sites (ANOVA: *F* = 1.87, *p* = 0.05; Table S11) with T8 again showing the highest diversity (Figure S5B) while differing significantly from T21 (Tukey: *p* = 0.04; Table S11) and T22 (Tukey: *p* = 0.02; Table S11). Hence, two out of 78 comparisons (2.56%) were significant. Thus, there was greater inter‐site variation in gut bacterial communities in *B. terrestris* than in *A. mellifera*.

### 3.4 Environmental Effects on the Bee Gut Microbiome

The two bee species differed significantly with respect to the effect of precipitation on facultative gut bacterial diversity (*lmer*: *p* = 0.004; Table S12), overall bacterial richness (*lmer*: *p* = 0.01; Table S13) and facultative bacterial richness (*lmer*: *p* = 0.03; Table S13). Specifically, in *B. terrestris* , precipitation was positively correlated with facultative bacterial diversity (*lmer*: *p* = 0.02, *r* ^2^ = 0.48; Table S14; Figure S6A), overall bacterial diversity (*lmer*: *p* = 0.05, *r* ^2^ = 0.35; Table S14; Figure S6B), facultative bacterial richness (*lmer*: *p* = 0.01, *r* ^2^ = 0.41; Table S15; Figure S6C), and overall bacterial richness (*lmer*: *p* = 0.05, *r* ^2^ = 0.48; Table S15; Figure S6D). The overall bacterial richness of *B. terrestris* was also significantly predicted by the interaction between precipitation × pasture (*lmer*: *p* = 0.001; Table S15; Figure S7A). For *A. mellifera* , this interaction effect was also significant for overall bacterial richness (*lmer*: *p* = 0.03; Table S15; Figure S7B), and facultative bacterial richness (*lmer*: *p* = 0.02; Table S15; Figure S7C). Together, this suggests that the *B. terrestris* facultative gut microbiome is particularly sensitive to precipitation, while combined effects of precipitation and pasture may shape gut bacterial richness in both bee species.

**FIGURE 4:**
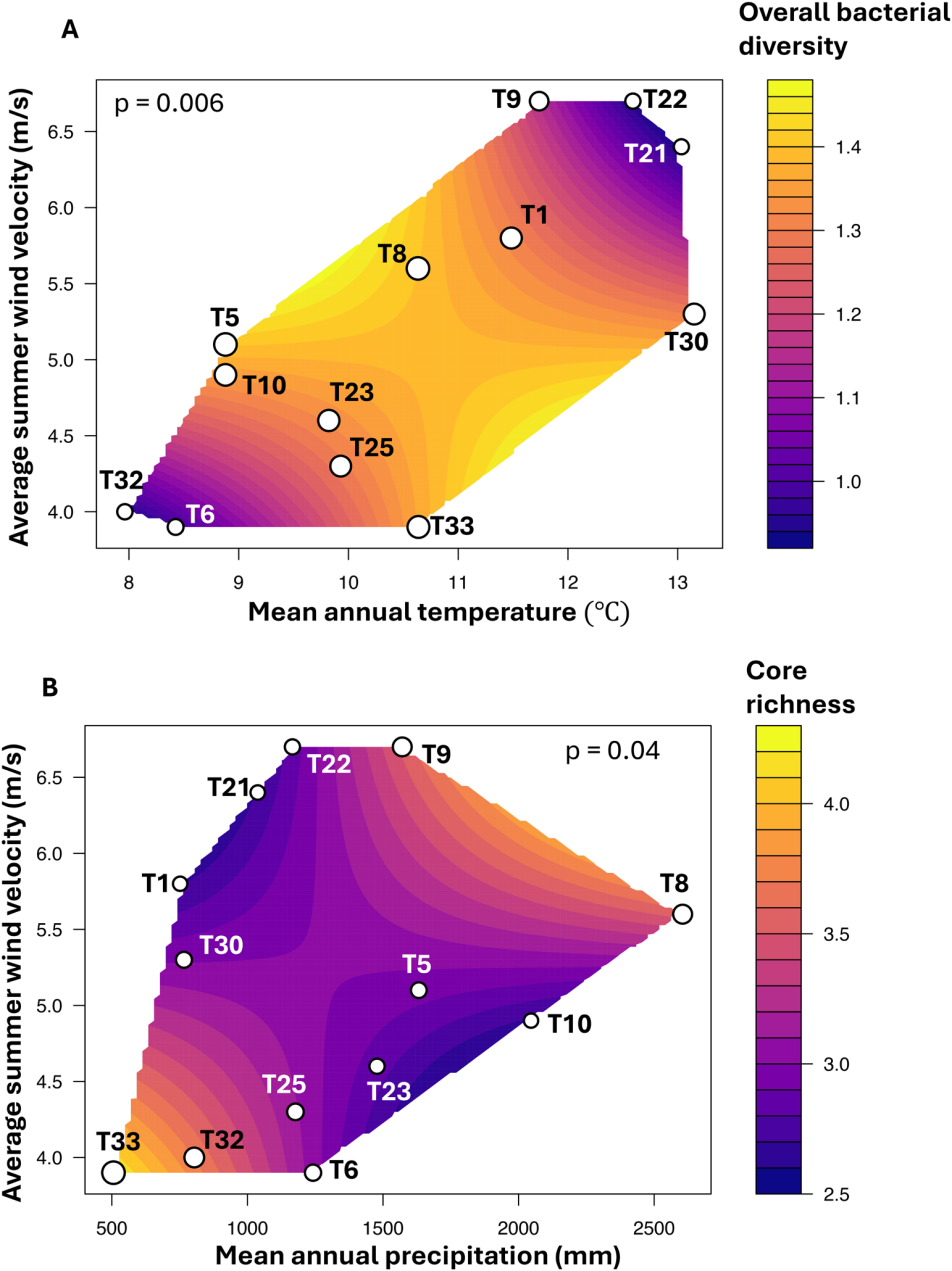
Interaction effects of—(A) wind velocity and temperature on overall gut bacterial diversity of *B. terrestris* , (B) wind velocity and precipitation on core gut bacterial richness of *B. terrestris*. Diversity measure = Shannon; Richness measure = Chao1.

The influence of wind velocity on core bacterial diversity varied significantly between the two species (*lmer*: *p* = 0.03; Table S12). In *B. terrestris* , wind velocity was negatively correlated with core bacterial diversity (*lmer*: *p* = 0.04, *r* ^2^ = 0.30; Table S14; Figure S8A), while this relationship was not observed in *A. mellifera* (*lmer*: *p* = 0.69; Table S16). Moreover, in *B. terrestris* , overall bacterial diversity could be predicted by an interaction between wind velocity and temperature (*lmer*: *p* = 0.006; Table S14; Figure 4A). Core bacterial richness in *B. terrestris* could also be predicted by an interaction between precipitation and wind velocity (*lmer*: *p* = 0.04; Table S15; Figure 4B), another pattern that was absent in *A. mellifera* (*p* = 0.44; Table S17). Wind velocity showed a negative correlation with the overall diversity of pollen foraged by *B. terrestris* (*lm*: *p* = 0.05, *r* ^2^ = 0.34; Table S18; Figure S8B). Therefore, wind velocity, both independently and through interactions with other environmental variables, played a greater role in shaping the gut microbiome and foraging patterns of *B. terrestris* than *A. mellifera* .

### 3.5 Corbicular Pollen Taxonomic Diversity and Relative Abundance

**FIGURE 5:**
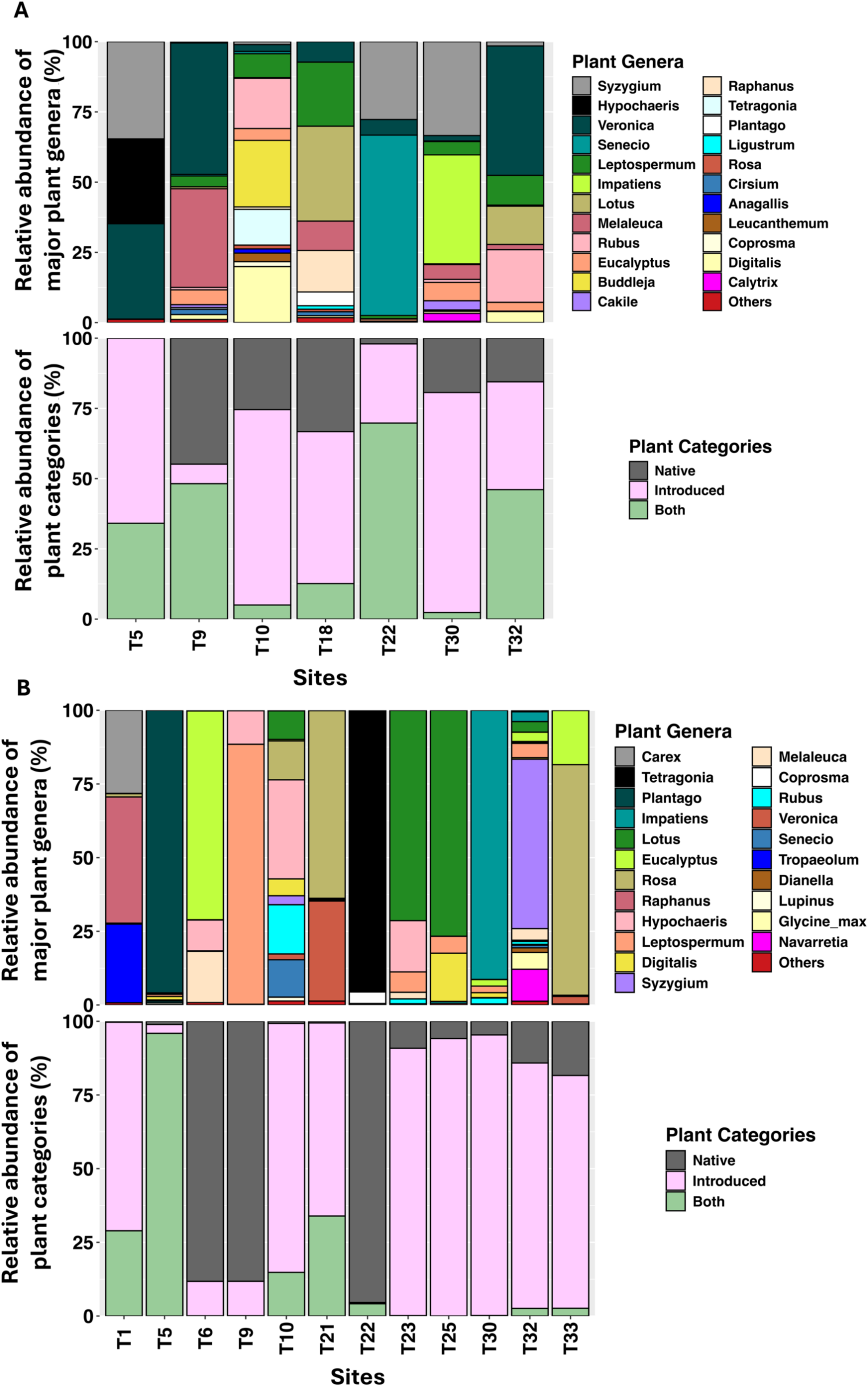
Composition of plant sources collected from pollen baskets of (A) *A. mellifera* and (B) *B. terrestris* . Both (A) and (B) show the relative abundance of major plant (> 1%) genera (top panels) and corresponding plant categories (native, introduced or both; bottom panels) for *A. mellifera* and *B. terrestris* , respectively, across sampled sites. In the ‘Plant Genera’ legend, ‘Others’ denote sum of all plant genera with relative abundance of less than 1%.

ITS2 analysis showed that *A. mellifera* foraged on 34 plant genera and *B. terrestris* on 52 (Figure S9). Of these, 23 and 22 genera, respectively, contributed > 1% of total reads (Figure 5A–B, top panels). The two species shared 23 genera (44.2% overlap), including four native (*Tetragonia*, *Eucalyptus*, *Leptospermum*, and *Melaleuca*), 14 introduced (*Hypochaeris, Impatiens, Lotus, Rubus, Raphanus, Syzygium, Rosa, Cirsium, Anagallis, Trifolium, Medicago, Tropaeolum, Linaria*, and *Digitalis*), and five classified as ‘both’ (*Carex*, *Coprosma*, *Plantago*, *Senecio*, and *Veronica*), reflecting a diverse and partially overlapping floral diet (Figure S9). Relative abundances suggested that both bee species mainly foraged on introduced plants, with *A. mellifera* averaging 48.8% and *B. terrestris* 57.5% introduced pollen (Tables S19 and S20). Introduced genera dominated at 4 of 7 *A. mellifera* sites and 8 of 12 *B. terrestris* sites (Figure 5A,B, bottom panels), indicating notable reliance, particularly for *B. terrestris* , on non‐native floristic resources.

### 3.6 Pollen Diversity and the Bee Gut Microbiome

**FIGURE 6:**
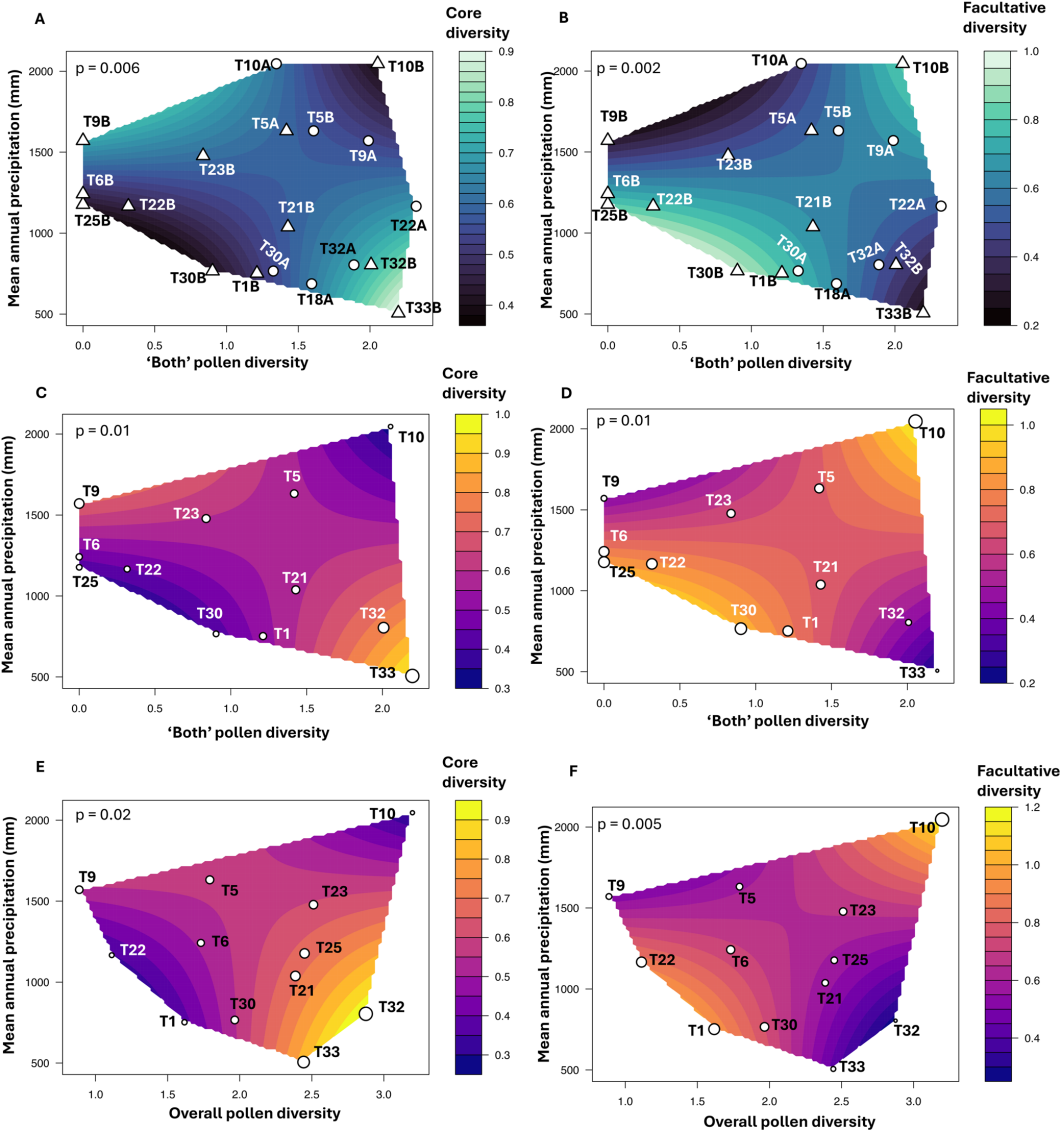
Precipitation × pollen interactions. (A, B) Species variation in the interaction between precipitation and ‘both’ pollen diversity on (A) core and (B) facultative gut bacterial diversity. Site abbreviations (A = *Apis* and B=*Bombus*) are included alongside site names; (C, D) Effects of precipitation and ‘both’ pollen diversity on (C) core and (D) facultative gut bacterial diversity in *B. terrestris* ; (E–F) Effects of precipitation and overall pollen diversity on (E) core and (F) facultative gut bacterial diversity in *B. terrestris* . In (A) and (B), circles represent *A. mellifera* sites and triangles represent *B. terrestris* sites; Diversity measure = Shannon.

Significant species‐level differences were observed in the interaction between precipitation × ‘both’ (i.e., genera that are both native and introduced) pollen (Shannon) diversity on core (*lmer*: *p* = 0.006; Table S12; Figure 6A) and facultative (*lmer*: *p* = 0.002; Table S12; Figure 6B) bacterial diversity. These effects can be explained by the response of *B. terrestris* , for which core (*lmer*: *p* = 0.01; Table S14; Figure 6C) and facultative (*lmer*: *p* = 0.01; Table S14; Figure 6D) bacterial diversity was significantly associated with the interaction between precipitation × ‘both’ pollen diversity. Additionally, the interaction between precipitation × overall pollen diversity showed significant associations with both the core (*lmer*: *p* = 0.02; Table S14; Figure 6E) and facultative (*lmer*: *p* = 0.005; Table S14; Figure 6F) gut bacterial diversity of *B. terrestris*. These patterns were absent in *A. mellifera* (*lmer*: *p* > 0.05; Table S16). The interaction between foraged pollen diversity and precipitation was therefore more influential on both core and facultative gut microbiome of *B. terrestris* compared to *A. mellifera*.

**FIGURE 7:**
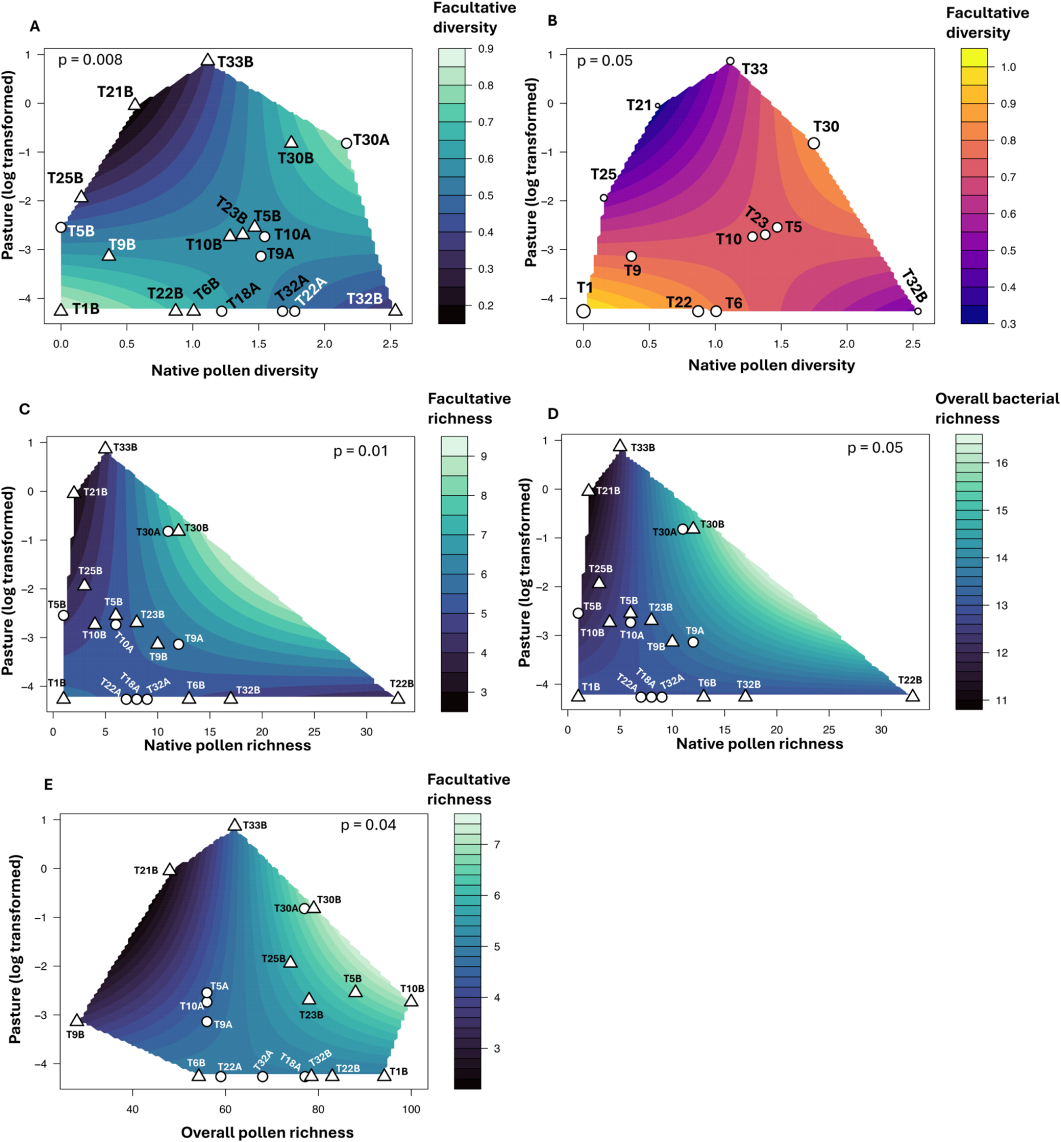
Pasture × pollen interactions. (A) Species variation in the interaction effect of pasture and native pollen diversity on facultative gut bacterial diversity. Site abbreviations (A = *Apis* and B = Bombus) are included alongside site names; (B) Effects of pasture and native pollen diversity on facultative gut bacterial diversity of *B. terrestris* . (C, D) Species variation in the interaction between pasture and native pollen richness on (C) facultative and (D) overall gut bacterial richness. (E) Species variation in the interaction between pasture and overall pollen richness on facultative gut bacterial richness. In (A–E), circles represent *Apis* sites and triangles represent *Bombus* sites; Diversity measure = Shannon; Richness measure = Chao1.

The interaction effect between pasture × native pollen diversity on facultative gut bacterial diversity differed between species (*lmer*: *p* = 0.008; Table S12; Figure 7A). In *B. terrestris* , this interaction was significantly associated with facultative bacterial diversity (*lmer*: *p* = 0.05; Table S14; Figure 7B) whereas no significant effect was observed in *A. mellifera* (*lmer*: *p* = 0.20; Table S16). Species‐level differences were also evident in the interaction effect between pasture × native pollen (Chao1) richness on facultative bacterial richness (*lmer*: *p* = 0.01; Table S13; Figure 7C) and overall bacterial richness (*lmer*: *p* = 0.05; Table S13; Figure 7D). The interaction effect between pasture × overall pollen richness on facultative gut bacterial richness also differed between species (*lmer*: *p* = 0.04; Table S13; Figure 7E). Hence, the facultative gut microbiome of *B. terrestris* was more responsive than *A. mellifera* to the combined effects of land use and native foraged pollen diversity.

The interaction effect of temperature and native pollen diversity on facultative bacterial diversity (*lmer*: *p* = 0.05; Table S12; Figure S10A) and overall bacterial diversity (*lmer*: *p* = 0.02; Table S12; Figure S10B) significantly varied between species, suggesting that native floristic diversity and thermal conditions jointly influence the gut microbial diversity of both species. In *A. mellifera* , facultative bacterial richness was negatively correlated with introduced (*lmer*: *p* = 0.03, *r* ^2^ = 0.67; Table S17; Figure S11A) and overall (*lmer*: *p* = 0.03, *r* ^2^ = 0.67; Table S17; Figure S11B) pollen richness, but there were no significant interactions between pollen diversity and environmental factors (*lmer*: all *p* > 0.05; Table S16). However, the overall richness of foraged pollen was negatively correlated with precipitation in *A. mellifera* (*lm*: *p* = 0.009, *r* ^2^ = 0.78; Table S21; Figure S12).

## 4 Discussion

Here we uncover distinct species‐specific responses of the gut microbiomes of *A. mellifera* and *B. terrestris* to local environmental variables and foraged pollen diversity within an introduced landscape. In *A. mellifera* , gut bacterial composition was significantly associated with mean annual temperature, whereas in *B. terrestris* , it was shaped by mean annual precipitation and pasture percentage (Table S6; Figure 3). In *B. terrestris*, overall and facultative gut bacterial diversity and richness was also positively influenced by precipitation (Tables S14 and S15; Figure S6), core and facultative bacterial diversity was predicted by the interaction between precipitation and pollen diversity (Table S14, Figure 6); patterns that were absent in *A. mellifera* (Table S16). Furthermore, in *B. terrestris*, average summer wind velocity was negatively correlated with core bacterial diversity (Table S14; Figure S8A), and the interaction between wind with precipitation and temperature could predict core bacterial richness and overall bacterial diversity, respectively (Tables S15 and S14; Figure 4). Finally, the two species differed in their gut microbial response to site pasture percentage and pollen diversity (Tables S12 and S13; Figure 7). While the different life‐histories of both species inherently shape their gut microbiomes rather than time since colonisation, our findings reinforce that the recently invaded *B. terrestris* harbours a more environmentally responsive gut microbiome than the longer established *A. mellifera* . This may reflect inter‐specific differences in colony size, habitat and behaviour, while also supporting the survival and persistence of *B. terrestris* in novel areas.

### 4.1 Effect of Temperature, Precipitation and Pollen Diversity

In *A. mellifera*, we found that mean annual temperature was a significant driver of gut bacterial composition (Table S6; Figure 3A). Temperature affects insect metabolism, development, and survival (Bale et al. 2002; Zuo et al. 2012; Kühsel and Blüthgen 2015), with heat stress reducing bee fitness through desiccation (Hamblin et al. 2018). These effects vary by species, often linked to body size (Burdine and McCluney 2019), underscoring the complex, taxon‐specific impacts of climate on pollinator microbiomes. Mean annual precipitation was a major driver of gut bacterial composition in *B. terrestris* (Table S6; Figure 3B), positively correlating with overall and facultative diversity and richness (Tables S14 and S15; Figure S6). This supports previous findings in *Bombus pyrosoma* (Zhang et al. 2024) and Tasmanian *B. terrestris* (Kardum Hjort et al. 2023, 2024; Haque et al. 2025). Notably, site T8 for *B. terrestris* had the highest precipitation, and its microbiome was significantly divergent from all other sites. Our previous work found that a nearby site with similarly high precipitation (S9) showed a divergent microbiome in *B. terrestris* (Haque et al. 2025). Rainfall may impact microbiomes by limiting foraging and disrupting visual cues (Totland 1994), while many pollinators reduce flight in response to weather changes (Lawson and Rands 2019). Evidence for local adaptation in Tasmanian *B. terrestris* included identification of candidate genes related to cuticle water retention and precipitation seasonality (Kardum Hjort et al. 2024), highlighting an additional role of rainfall in evolutionary traits.

The interaction between precipitation and overall pollen diversity significantly influenced core and facultative gut bacterial diversity in *B. terrestris* (Table S14; Figure 6). While *B. terrestris* was more responsive to rainfall than *A. mellifera* , the latter showed a negative correlation between precipitation and pollen richness (Table S21; Figure S12). Rainfall can reduce floral quality by degrading pollen and diluting nectar—reducing pollen viability and attractiveness to bees (Burke 2002; Sun et al. 2008), while diluted nectar may discourage foraging (Cnaani et al. 2006). Thus, precipitation may affect bee gut microbiomes indirectly by altering both the quality and diversity of floral resources.

### 4.2 Effect of Pasture, Pollen Diversity and Wind Velocity

The two bee species differed in the effect of pasture × pollen diversity on facultative gut bacterial diversity and richness (Tables S12 and S13; Figure 7). Pasture significantly shaped gut bacterial composition in *B. terrestris* (Table S6; Figure 3B), consistent with prior findings in Tasmania (Kardum Hjort et al. 2023, 2024; Haque et al. 2025), but this was not upheld in the present study for *A. mellifera* . Grazed pastures, often low in floristic and nesting resources due to trampling and flower loss (Kearns et al. 1998), are linked to reduced pollinator visitation (Robson 2019) and flowering suppression (Debano 2006). Early‐season grazing is also known to lower bumblebee abundance and diversity (Kimoto et al. 2012). Notably, *B. terrestris* from pasture‐dominated sites in Tasmania showed shorter proboscis lengths, suggesting morphological adaptation to altered floral traits (Kardum Hjort et al. 2023). These findings highlight how land use may shape both foraging behaviour and the gut microbiome in *B. terrestris* , underscoring the role of human‐modified landscapes in pollinator health and adaptation.

In *B. terrestris* , core gut bacterial diversity declined with increasing wind velocity (Table S14; Figure S8A), a pattern not seen in *A. mellifera* . Core bacterial richness could also be predicted by interactions between wind × precipitation, and overall diversity by wind × temperature in *B. terrestris* (Tables S15 and S14; Figure 4A–B). Wind can disrupt bee foraging activity by impairing flight and landing stability (Combes and Dudley 2009; Tuell and Isaacs 2010; Chang et al. 2016), with larger bumblebees particularly affected (Goyal et al. 2024). Genomic evidence of wind velocity‐associated selection in Tasmanian *B. terrestris* (Kardum Hjort et al. 2024) further suggests that wind may act as a selection pressure in the region.

### 4.3 Role of Gut Microbiome in Host Fitness and Invasion Success

The facultative bacterial genera we found support key traits for colonising novel environments, including immunity, detoxification, and pathogen defence (Kwong et al. 2018; Li et al. 2022). While explicit studies of *Apis* and *Bombus* gut microbiomes within invasive ranges are lacking for comparison, a study on native and invasive resin bees (*Megachile sculpturalis*) found that bees from invasive regions shared a more similar gut microbiota and an absence of pathogens (Tuerlings et al. 2023). The five core gut bacterial genera we found in both *A. mellifera* and *B. terrestris* (Figure 2), align with previous studies (Hammer et al. 2021; Carlini et al. 2024) and are known to aid pollen and nectar digestion via enzymes like pectin lyases and glycoside hydrolases (Engel et al. 2012; Zheng et al. 2019; Kešnerová et al. 2017). While the functions of the gut microbiome are diverse with many aspects unknown, there is evidence showing that the gut microbiome shapes social interactions within bee colonies, reinforcing nestmate bonds and influencing the development of specialised roles (Vernier et al. 2020). These effects, likely mediated through changes in brain metabolites and transcriptomic profiles, highlight the gut microbiome's involvement in the gut–brain axis that regulates behaviours like foraging and division of labour, thus underscoring its importance in bee health and colony organisation (Liberti et al. 2023). The gut microbiome also helps to protect bees from pathogens by competing for nutrients, reducing gut pH and oxygen levels (Palmer‐Young et al. 2019), and potentially inhibiting harmful microbes (Steele et al. 2021). It also activates the innate immune system and promotes the production of antimicrobial peptides, supporting colony health (Danihlík et al. 2015), enabling bees to withstand pathogen invasion. Collectively, these microbiome‐driven processes enhance physiological resilience and social organisation, providing a competitive advantage that may facilitate the successful establishment and persistence of introduced bee species in novel environments.

Although sampling sites encompassed urban, rural, and residential areas, sites were not classified as such in statistical models, and we chose to use continuous environmental variables that capture broader climatic and landscape gradients that were previously associated with bee microbiomes in Tasmania (Haque et al. 2025). We acknowledge that local land‐use or site habitat type can influence floral composition and microclimate, which may affect bee gut microbiomes and pollen foraging (Nguyen and Rehan 2023; Peters et al. 2025) but these variables may also correlate with landscape‐scale environmental variables. Future studies employing balanced, replicated sampling designs across land‐use or vegetation categories will be required to explicitly disentangle habitat‐specific effects from correlated environmental gradients.

Our study provides important insights into the potential role of the gut microbiome and environmental factors in supporting the persistence and spread of introduced pollinators, though we acknowledge our sampling represents a single temporal snapshot. While our findings for *B. terrestris* are largely concordant with previous work (Kardum Hjort et al. 2023, 2024; Haque et al. 2025), seasonal sampling would help to capture temporal variation for both species. Expanding the number of sampling sites, especially with a broader distribution of the percentage of pasture, may help to strengthen our conclusions regarding the effect of pastural land use on bee gut health. Furthermore, using seasonal and annual climatic averages could be augmented with higher resolution environmental data. Despite this, we effectively uncover gut microbiome–environment relationships in wild‐caught bees across a diverse landscape. While the interspecific patterns we observe are likely to be driven by inherent species differences rather than residence time, our findings provide a reference for future studies to examine how bee microbiomes within species facilitate invasion across different geographic regions.

## 5 Conclusion

Here we compare landscape‐scale predictors of gut microbiomes in a long‐established pollinator, *A. mellifera* , and a recently invasive pollinator, *B. terrestris* , and found species‐specific links to variation in foraged pollen resources and local environmental conditions. Our findings underscore the sensitivity of the bee gut microbiome to abiotic and biotic factors, with *B. terrestris* showing stronger environmental associations than *A. mellifera* , particularly with respect to precipitation, wind, and pasture. Our sampling across Tasmania offers valuable baseline data on bee health prior to the imminent arrival of *Varroa destructor* on the island, following its 2022 incursion into mainland Australia (Chapman et al. 2023). Although direct measures of bee health were not assessed, gut microbiome structure and pollen foraging profiles are increasingly recognised as indicators of immune function, pathogen susceptibility, and environmental exposure in bees. This baseline is particularly relevant in the context of *V. destructor* , whose establishment is closely linked to changes in viral dynamics, immune dysregulation, and microbiome composition in honeybee populations. As such, the patterns documented here offer an important reference framework for future monitoring of bee microbiomes, viruses, foraging shifts associated with the spread of *V.* *destructor*, environmental change, and ongoing population establishment in Tasmania.

## Funding

This work was supported by the Australian Research Council (FT230100478); Macquarie University; and Bioplatforms Australia.

## Conflicts of Interest

The authors declare no conflicts of interest.

## Supporting information


**Text S1:** Correlations among environmental variables.
**Text S2:** Bee gut bacterial DNA extractions.
**Text S3:** 16S rRNA library preparation and sequencing.
**Text S4:** 16S rRNA data processing using QIIME‐2.
**Text S5:** Pollen collection, DNA extraction and PCR.
**Table S1:** Pearson correlation matrix of environmental variables across sites where *A. mellifera* and *B. terrestris* were sampled. Values above the diagonal correspond to *A. mellifera* , and those below the diagonal correspond to *B. terrestris* . Bolded values indicate strong correlations (*r* ≥ 0.7). Temperature and wind velocity were strongly correlated for *B. terrestris* study sites. N/A, not applicable; Pasture, percentage of pasture (%); Rain, mean annual precipitation (mm); Temp, mean annual temperature (°C); Wind, average summer wind velocity (m/s).
**Table S2:** Total number of features (read count) per sample for *A. mellifera* gut microbiome (*n* = 100) following quality filtering.
**Table S3:** Total number of features (read count) per sample for *B. terrestris* gut microbiome (*n* = 88) following quality filtering.
**Table S4:** Summary of sequencing read counts for *A. mellifera* pollen samples (*N* = 7) following DADA2 pipeline.
**Table S5:** Summary of sequencing read counts for *B. terrestris* pollen samples (*N* = 12) following DADA2 pipeline.
**Table S6:** Squared correlations (*r* ^2^) of environmental variables with the site scores on NMDS axis 1 for *A. mellifera* and *B. terrestris* based on Bray‐Curtis dissimilarities. Correlations that are statistically significant (*p* ≤ 0.05) are highlighted in bold for both datasets. Refer to Figure 3 and Figure S2 for corresponding NMDS ordination plots.
**Table S7:** Pairwise PERMANOVA showing significant differences in *A. mellifera* gut bacterial communities between sites. Cells with bolded *p*‐values indicate statistically significant differences between sites (*p* ≤ 0.05). The *p*‐values were Bonferroni‐adjusted to control for multiple comparisons.
**Table S8:** Pairwise PERMANOVA showing significant differences in *B. terrestris* gut bacterial communities between sites. Cells with bolded *p*‐values indicate statistically significant differences between sites (*p* ≤ 0.05). The *p*‐values were Bonferroni‐adjusted to control for multiple comparisons.
**Table S9:** Summary of PCoA statistics showing the results of environmental vector fitting (envfit), including squared correlations (*R* ^2^) values and permutation‐based (999) *p*‐values, for *A. mellifera* and *B. terrestris* gut microbiomes based on Jaccard and Bray‐Curtis dissimilarities. Correlations that are statistically significant (*p* ≤ 0.05) are highlighted in bold for both datasets. Refer to Figure S3 for corresponding PCoA ordination plots.
**Table S10:** Summary of Tukey post hoc tests assessing the statistical significance of pairwise differences among sites in average alpha diversity (Shannon and Chao1) for gut microbiomes of *A. mellifera* . The *p*‐values are respectively listed above and below the diagonal for the Shannon and Chao1 indices, with significant *p*‐values (*p* ≤ 0.05) in bold. One‐way ANOVA indicated that site ID was a significant predictor of diversity variation among samples for the Chao1 index (*F*_10,89_ = 2.44, *p* = 0.01), but not the Shannon index (*F*_10,89_ = 1.01, *p* = 0.44). Abbreviation: N/A = Not applicable.
**Table S11:** Summary of Tukey post hoc tests assessing the statistical significance of pairwise differences among sites in average alpha diversity (Shannon and Chao1) for gut microbiomes of *B. terrestris* . The *p*‐values are respectively listed above and below the diagonal for the Shannon and Chao1 indices, with significant *p*‐values (*p* ≤ 0.05) in bold. One‐way ANOVA indicated that site ID was a highly significant predictor of diversity variation among samples for the Chao1 index (*F*_12,75_ = 7.66, *p* = 4.75 × 10^−9^), and a marginally significant predictor for the Shannon index (*F*_12,75_ = 1.87, *p* = 005). Abbreviation: N/A = Not applicable.
**Table S12:** Tests used to evaluate the significance of the highest order interaction or main effect (if no interaction was present) involving bee species (two levels), pollen (Shannon) diversity, and/or environmental variables on gut bacterial (Shannon) diversity. Statistically significant (*p* ≤ 0.05) interactions are emphasised in bold. Abbreviations: Temp = Mean annual temperature, Rain = Mean annual precipitation, Wind = Average summer wind velocity, log_pasture = logit transformed values for percentage of pasture, DF = Degrees of freedom, Fac bacterial diversity = Facultative bacterial diversity. The asterisk (*) within predictor variables denotes a model with main effect and interaction.
**Table S13:** Tests used to evaluate the significance of the highest order interaction or main effect (if no interaction was present) involving bee species (two levels), pollen (Chao1) richness, and/or environmental variables on gut bacterial (Chao1) richness. Statistically significant (*p* ≤ 0.05) interactions are emphasized in bold. Abbreviations: Temp = Mean annual temperature, Rain = Mean annual precipitation, Wind = Average summer wind velocity, log_pasture = logit transformed values for percentage of pasture, DF = Degrees of freedom, Fac bacterial richness = Facultative bacterial richness. The asterisk (*) within predictor variables denotes a model with main effect and interaction.
**Table S14:** Tests used to evaluate the significance of the highest order interaction or main effect (if no interaction was present) involving *B. terrestris*, pollen (Shannon) diversity, and/or environmental variables on gut bacterial (Shannon) diversity. Statistically significant (*p* ≤ 0.05) interactions are highlighted in bold. Abbreviations: Temp = Mean annual temperature, Rain = Mean annual precipitation, Wind = Average summer wind velocity, log_pasture = logit transformed values for percentage of pasture, DF = Degrees of freedom, Fac bacterial diversity = Facultative bacterial diversity. The asterisk (*) within predictor variables denotes a model with main effect and interaction.
**Table S15:** Tests used to evaluate the significance of the highest order interaction or main effect (if no interaction was present) involving *B. terrestris*, pollen (Chao1) richness, and/or environmental variables on gut bacterial (Chao1) richness. Statistically significant (*p* ≤ 0.05) interactions are highlighted in bold. Abbreviations: Temp = Mean annual temperature, Rain = Mean annual precipitation, Wind = Average summer wind velocity, log_pasture = logit transformed values for percentage of pasture, DF = Degrees of freedom, Fac bacterial richness = facultative bacterial richness. The asterisk (*) within predictor variables denotes a model with main effect and interaction.
**Table S16:** Tests used to evaluate the significance of the highest order interaction or main effect (if no interaction was present) involving *A. mellifera*, pollen (Shannon) diversity, and/or environmental variables on gut bacterial (Shannon) diversity. All interactions were statistically insignificant (*p* > 0.05). Abbreviations: Temp = Mean annual temperature, Rain = Mean annual precipitation, Wind = Average summer wind velocity, log_pasture = logit transformed values for percentage of pasture, DF = Degrees of freedom, Fac bacterial diversity = facultative bacterial diversity. The asterisk (*) within predictor variables denotes a model with main effect and interaction.
**Table S17:** Tests used to evaluate the significance of the highest order interaction or main effect (if no interaction was present) involving *A. mellifera*, pollen (Chao1) richness, and/or environmental variables on gut bacterial (Chao1) richness. Statistically significant (*p* ≤ 0.05) interactions are shown in bold. Abbreviations: Temp = Mean annual temperature, Rain = Mean annual precipitation, Wind = Average summer wind velocity, log_pasture = logit transformed values for percentage of pasture, DF = Degrees of freedom, Fac bacterial richness = facultative bacterial richness. The asterisk (*) within predictor variables denotes a model with main effect and interaction.
**Table S18:** Tests to assess linear relationships between alpha diversity (Shannon's diversity and Chao1 richness) of pollen foraged by *B. terrestris* and environmental factors. Statistically significant (*p* ≤ 0.05) relationships are shown in bold. Abbreviations: Temp = Mean annual temperature, Rain = Mean annual precipitation, Wind = Average summer wind velocity, Pasture = Percentage of pasture.
**Table S19:** Percentage of different plant types foraged by *A. mellifera* across Tasmania. ‘Native’ indicates native (including endemic) plant genera in Australia; ‘introduced’ indicates plant genera that have been introduced or naturalised in Tasmania; ‘both’ indicates plant genera containing both native and introduced species in Tasmania.
**Table S20:** Percentage of different plant types foraged by *B. terrestris* across Tasmania. ‘Native’ indicates native (including endemic) plant genera in Australia; ‘introduced’ indicates plant genera that have been introduced or naturalised in Tasmania; ‘both’ indicates plant genera containing both native and introduced species in Tasmania.
**Table S21:** Tests to assess linear relationships between alpha diversity (Shannon's diversity and Chao1 richness) of pollen foraged by *A. mellifera* and environmental factors. Statistically significant (*p* ≤ 0.05) relationships are shown in bold. Abbreviations: Temp = Mean annual temperature, Rain = Mean annual precipitation, Wind = Average summer wind velocity, Pasture = Percentage of pasture.
**Figure S1:** Key bacterial families found in the guts of (A) *A. mellifera* and (B) *B. terrestris* across Tasmania. In both plots, ‘Others’ represents sum of all bacterial families with relative abundance of less than 1%, while ‘unassigned_uncultured’ refers to bacteria that could not be classified into any specific family.
**Figure S2:** NMDS ordination of gut bacterial communities for (A) *A. mellifera* and (B) *B. terrestris* based on Bray–Curtis dissimilarity of ASV abundance of individual samples. Stress for both NMDS plots = 0.2. Abbreviations: AT = Mean annual temperature (°C), AR = Mean annual precipitation (mm), PP = Percentage of pasture (%), WV = Average summer wind velocity (m/s). Refer to Table S6 for summary of environmental vector correlations (envfit; 999 permutations).
**Figure S3:** PCoA ordinations of gut microbiome composition based on Jaccard (A, C) and Bray–Curtis (B, D) dissimilarities for *A. mellifera* (A, B) and *B. terrestris* (C, D). Percent variance explained by the first two axes is shown on each axis. Abbreviations: AT = Mean annual temperature (°C), AR = Mean annual precipitation (mm), PP = Percentage of pasture (%), WV = Average summer wind velocity (m/s). Refer to Table S7 for summary of environmental vector correlations (envfit; 999 permutations).
**Figure S4:** Alpha diversity of *A. mellifera* gut microbiomes across Tasmania. (A) Chao1 richness of *A. mellifera* per site. All sites showed statistical significance (ANOVA: *p* = 0.01) and pairwise site comparisons revealed T9 significantly differed from T25 (Tukey: *p* = 0.03). (B) Shannon's diversity of *A. mellifera* per site. All sites were statistically insignificant (ANOVA: all *p* > 0.05). Refer to Table S5 for all corresponding ANOVA and Tukey post hoc results for *A. mellifera* alpha diversity.
**Figure S5:** Alpha diversity of *B. terrestris* bee gut microbiomes across Tasmania. (A) Chao1 richness of *B. terrestris* per site. All sites showed statistical significance (ANOVA: *p* = 4.75e^−9^) with T8 significantly differing from all other sites (Tukey: *p* < 0.001) (B) Shannon's diversity of *B. terrestris* per site. All sites showed marginal significance (ANOVA: *p* = 0.05) with T8 differing from T21 (Tukey: *p* = 0.04) and T22 (Tukey: *p* = 0.02). Refer to Table S6 for all corresponding ANOVA and Tukey post hoc results for *B. terrestris* alpha diversity.
**Figure S6:** Positive relationships between mean annual precipitation and (A) facultative gut bacterial diversity, (B) overall gut bacterial diversity, (C) facultative gut bacterial richness, (D) overall gut bacterial richness of *B. terrestris* across Tasmania. Measure of diversity = Shannon; Measure of richness = Chao1.
**Figure S7:** Interaction effect of pasture × precipitation on (A) overall gut bacterial richness of *B. terrestris*. (B) overall gut bacterial richness of *A. mellifera* and (C) facultative gut bacterial richness of *A. mellifera*. Richness measure = Chao1.
**Figure S8:** The negative correlation between average summer wind velocity and (A) core gut bacterial diversity of *B. terrestris*. (B) Overall diversity of pollen foraged by *B. terrestris*. Diversity measure = Shannon.
**Figure S9:** Heatmap displaying all plants identified from the pollen baskets of (A) *A. mellifera* and (B) *B. terrestris* . In both plots, the plant genera are categorized as native, introduced, or ‘both’. The colour scales indicate the sum of ASVs of different plant genera per site. *Calytrix is an endemic plant genus in Australia.
**Figure S10:** Species variation in response of the interaction between native pollen diversity and mean annual temperature on (A) facultative and (B) overall gut bacterial diversity. In both plots, circles represent *Apis* sites and triangles represent *Bombus* sites; site abbreviations (A = *Apis* and B = *Bombus*) are included alongside site names. Diversity measure = Shannon.
**Figure S11:** Positive correlations between facultative gut bacterial richness and (A) introduced pollen richness, (B) overall pollen richness of *A. mellifera* across Tasmania. Richness measure = Chao1.
**Figure S12:** The negative relationship between mean annual precipitation and overall richness of pollen foraged by *A. mellifera*.

## Data Availability

Tables of ASVs for 16S and ITS2 data; Alpha diversity values for each sample and environmental factors; R scripts for linear mixed effect models are all available on DRYAD at this temporary link for reviewers prior to publishing: http://datadryad.org/share/_uXhFLsTx7hkGquTQgXXbzsPokqhnmEPczCsuWOO074. Raw sequence data can be found here: https://mqoutlook‐my.sharepoint.com/:f:/g/personal/rachael_dudaniec_mq_edu_au/IgB7brFOapvgTbjQ2vQ‐LnyGAdfA_aKtqi3QmR050G6pAwY?e=JvetOh, and NCBI.
