# Correction to “Environment and Pollen Diversity Differentially Affect the Gut Microbiomes of Introduced Honeybees and Bumblebees”

**DOI:** 10.1111/eva.70279

**Published:** 2026-06-01

S. Haque, F. Ponton, A. P. Allen, et al. 2026. “Environment and Pollen Diversity Differentially Affect the Gut Microbiomes of Introduced Honeybees and Bumblebees.” *Evolutionary Applications* 19, no. 4: e70234. https://doi.org/10.1111/eva.70234.

In the “Data Availability Statement,” the following text was incorrect:

Tables of ASVs for 16S and ITS2 data; Alpha diversity values for each sample and environmental factors; R scripts for linear mixed effect models are all available on DRYAD at this temporary link for reviewers prior to publishing: http://datadryad.org/share/_uXhFLsTx7hkGquTQgXXbzsPokqhnmEPczCsuWOO074. Raw sequence data can be found here: https://mqoutlook‐my.sharepoint.com/:f:/g/personal/rachael_dudaniec_mq_edu_au/IgB7brFOapvgTbjQ2vQ‐LnyGAdfA_aKtqi3QmR050G6pAwY?e=JvetOh, and NCBI.

This has been replaced with:

Tables of ASVs for 16S and ITS2 data; Alpha diversity values for each sample and environmental factors; R scripts for linear mixed effect models are all available on DRYAD: https://doi.org/10.5061/dryad.ngf1vhj6r. Raw sequencing data for 16S and ITS2 have been deposited in the NCBI Sequence Read Archive (SRA) under BioProject accession number PRJNA1450835 and are accessible through this link: https://www.ncbi.nlm.nih.gov/sra/PRJNA1450835.

