# Supplementary material for: Environment and Pollen Diversity Differentially Affect the Gut Microbiomes of Introduced Honeybees and Bumblebees
Source: Evol Appl. 2026 Apr 13;19(4):e70234. doi: 10.1111/eva.70234 (PMC13076359; doi:10.1111/eva.70234)
Supplement: Supplementary file 1 — Text S1: Correlations among environmental variables. Text S2: Bee gut bacterial DNA extractions. Text S3: 16S rRNA library preparation and sequencing. Text S4: 16S rRNA data processing using QIIME‐2. Text S5: Pollen collection, DNA extraction and PCR. Table S1: Pearson correlation matrix of environmental variables across sites where A. mellifera and B. terrestris were sampled. Values above the diagonal correspond to A. mellifera , and those below the diagonal correspond to B. terrestris . Bolded values indicate strong correlations (r ≥ 0.7). Temperature and wind velocity were strongly correlated for B. terrestris study sites. N/A, not applicable; Pasture, percentage of pasture (%); Rain, mean annual precipitation (mm); Temp, mean annual temperature (°C); Wind, average summer wind velocity (m/s). Table S2: Total number of features (read count) per sample for A. mellifera gut microbiome (n = 100) following quality filtering. Table S3: Total number of features (read count) per sample for B. terrestris gut microbiome (n = 88) following quality filtering. Table S4: Summary of sequencing read counts for A. mellifera pollen samples (N = 7) following DADA2 pipeline. Table S5: Summary of sequencing read counts for B. terrestris pollen samples (N = 12) following DADA2 pipeline. Table S6: Squared correlations (r 2) of environmental variables with the site scores on NMDS axis 1 for A. mellifera and B. terrestris based on Bray‐Curtis dissimilarities. Correlations that are statistically significant (p ≤ 0.05) are highlighted in bold for both datasets. Refer to Figure 3 and Figure S2 for corresponding NMDS ordination plots. Table S7: Pairwise PERMANOVA showing significant differences in A. mellifera gut bacterial communities between sites. Cells with bolded p‐values indicate statistically significant differences between sites (p ≤ 0.05). The p‐values were Bonferroni‐adjusted to control for multiple comparisons. Table S8: Pairwise PERMANOVA showing significant differences in B. ter [file EVA-19-e70234-s001.docx]

**SUPPLEMENTARY MATERIAL**

***Text S1: Correlations among environmental variables***

The Pearson correlation matrix revealed no correlations ≥0.70 among the four environmental variables for sites sampled for *Apis mellifera* (N=11 sites; Table S1). In contrast, for sites at which *Bombus terrestris* was sampled (N=13 sites; 10 overlapping with *A. mellifera*), one positive correlation ≥0.70 was found between mean annual temperature and average summer wind velocity (r = 0.75, Table S1). Despite this, neither of the environmental variables were excluded to ensure consistency in the comparative analysis of both bee species, yet we account for this in our interpretations.

***Text S2. Bee gut bacterial DNA extractions***

Gut dissections of the midgut and hindgut were performed on five to ten *A. mellifera* and four to eight *B. terrestris* per site across 11 and 13 sites, respectively. Each bee was first rinsed with 70% ethanol, placed on a sterile petri dish, and immersed in sterile 1x PBS (137 mM NaCl, 2.7 mM KCl, 10 mM NaH_2_PO_4_, 1.8 mM KH_2_PO_4_). Under a binocular stereo microscope, the midgut and hindgut were isolated using pre-sterilized forceps and stored in 600 μl of 1x PBS. DNA extraction followed a modified DNeasy Blood and Tissue Kit protocol (Qiagen). Prior to extraction, a lysozyme-supplemented enzymatic lysis buffer (20 mM Tris-Cl, pH 8.0; 2 mM Na_2_EDTA; 1.2% Triton X-100) was added to the gut samples, along with glass beads (0.1 mm, Benchmark Scientific). The samples were incubated at 37°C for 45 minutes, then homogenized for 5 minutes at 30 Hz using a Tissue Lyser II (Qiagen), followed by another 45-minute incubation at 37°C. After lysis, 200 μl of Buffer AL and 25 μl of Proteinase-K (Ambion) were added, and the mixture was incubated at 56°C for 30 minutes. DNA extraction proceeded according to the manufacturer’s instructions. DNA concentration was measured using a Qubit 4 Fluorometer with the dsDNA High-Sensitivity Assay Kit (Invitrogen).

***Text S3. 16S rRNA library preparation and sequencing***

Amplicon sequencing of the 16S V4 region was performed at the Ramaciotti Centre for Genomics (University of New South Wales, Sydney, Australia). The full-length gene-specific primer sequences targeting the V4 region were as follows:

Forward Primer: 5' TCGTCGGCAGCGTCAGATGTGTATAAGAGACAGCCTACGGGNGGCWGCAG

Reverse Primer: 5' GTCTCGTGGGCTCGGAGATGTGTATAAGAGACAGGACTACHVGGGTATCTAATCC

Illumina overhang adapter sequences were added to the locus-specific primers for the target region:

Forward Overhang: 5' TCGTCGGCAGCGTCAGATGTGTATAAGAGACAG-[locus specific sequence]

Reverse Overhang: 5' GTCTCGTGGGCTCGGAGATGTGTATAAGAGACAG-[locus specific sequence]

PCR amplification of the 16S gene was carried out under the following conditions: an initial denaturation at 95°C for 3 minutes, followed by 25 cycles of denaturation at 95°C for 30 seconds, annealing at 55°C for 30 seconds, extension at 72°C for 30 seconds, and a final extension at 72°C for 5 minutes. After PCR amplification, the products were purified using AMPure XP beads to remove free primers and primer dimers.

Next, a dual indexing process was performed, and Illumina sequencing adapters were added through index PCR using the Nextera XT Index Kit (FC-131-1001). This PCR was conducted with an initial denaturation at 95°C for 3 minutes, followed by 8 cycles of 95°C for 30 seconds, 55°C annealing for 30 seconds, 72°C extension for 30 seconds, and a final extension at 72°C for 5 minutes. Following the index PCR, a secondary clean-up was done using AMPure XP beads. The library was then quantified and validated. A 1:50 dilution of the final library was run on a Bioanalyzer DNA 1000 chip to confirm the fragment size. The pooled libraries were denatured with NaOH, diluted in hybridization buffer, and heat-denatured before undergoing paired-end 2x250 sequencing on the Illumina MiSeq platform.

***Text S4. 16S rRNA data processing using QIIME-2***

Taxonomic identification of gut bacteria was performed using QIIME-2 (version 2022.8). Paired-end reads were demultiplexed and quality-filtered with the q2-demux plugin, then denoised using Deblur. Amplicon sequence variants (ASVs) were aligned with mafft via q2-alignment and a phylogenetic tree was constructed using fasttree2 in q2-phylogeny. Taxonomic classification of ASVs was done using a pre-trained Naïve Bayes classifier against the Silva-138 reference database for the 515F/R806 region of the 16S rRNA gene, via the q2-feature-classifier plugin. Genus-level assignments were confirmed with *blastn*.

***Text S5. Pollen collection, DNA extraction and PCR***

***Corbicular pollen collection***

Prior to gut dissections, we collected and pooled pollen from all bees sampled at a single site. The tube containing the bee and 70% ethanol was gently shaken to dislodge any attached pollen packets or grains, and the bee was removed from the tube, which was then centrifuged at 10,000 rpm for three minutes. The ethanol supernatant was carefully removed, and the pollen pellet was washed in 200μl of DNase/RNase-free water (Invitrogen, Life Technologies) and then transferred to a 2ml ‘master tube’ containing the pollen transferred from all bees at a single site. This tube was centrifuged again at 10000 rpm for five minutes. The water was removed from the sample, and the pollen was stored at –30$℃$ until DNA extraction.

***Pollen DNA extraction and PCR***

Corbicular pollen was pooled by site, and weighed, with 10–50 mg pollen per site used for DNA extraction. DNA was extracted from pooled pollen samples per site for each bee species separately using a modified protocol with the NucleoSpin Food Kit (Macherey Nagel). The CF (lysis) buffer was preheated to 65°C for 10 minutes, then added to each sample along with glass and zirconium oxide beads (2 mm, Lysing Matrix H; MP Biomedicals) for homogenization at 20 Hz for three minutes using Tissue Lyser II (Qiagen). Proteinase-K was added, and samples were incubated at 65°C for 1.5 hours. DNA was quantified with a Qubit 4 Fluorometer and a Qubit dsDNA High-Sensitivity kit, with DNA concentration standardised to 10 ng/µl. PCR amplification of the ITS2 region was performed using S2F/S3R primers (as in Chen et al., 2010). PCR amplification of the ITS2 region was performed with 12.5 μl of AmpliTaq Gold 360 MasterMix (Life Technologies), 0.5 μl each of forward primer S2F (5'-ATGCGATACTTGGTGTGAAT-3') and reverse primer S3R (5'-GACGCTTCTCCAGACTACAAT-3'), 8.5 μl of DNase/RNase-free water, and 3 μl of pollen DNA. The PCR cycle consisted of an initial denaturation at 94°C for 5 minutes, followed by 30 cycles of 94°C for 30 seconds, 56°C for 30 seconds, and 72°C for 45 seconds, with a final extension at 72°C for 10 minutes.

**SUPPLEMENTARY TABLES**

**Table S1.** Pearson correlation matrix of environmental variables across sites where A. mellifera and B. terrestris were sampled. Values above the diagonal correspond to A. mellifera, and those below the diagonal correspond to B. terrestris. Bolded values indicate strong correlations (r ≥ 0.7). Temperature and wind velocity were strongly correlated for B. terrestris study sites. **Abbreviations:** Temp = Mean annual temperature (℃), Rain = Mean annual precipitation (mm), Wind = Average summer wind velocity (m/s), Pasture = Percentage of pasture (%), N/A = Not applicable.

|  | **Temp** | Rain | Wind | Pasture |
| --- | --- | --- | --- | --- |
| Temp | N/A | –0.46 | 0.58 | 0.14 |
| Rain | –0.29 | N/A | 0.20 | –0.43 |
| **Wind** | **0.75** | 0.18 | N/A | –0.43 |
| Pasture | 0.36 | –0.53 | –0.16 | N/A |

**Table S2.** Total number of features (read count) per sample for *A. mellifera* gut microbiome (n=100) following quality filtering.

| Sample ID | Feature count |
| --- | --- |
| T10A14 | 118956 |
| T25C13 | 117356 |
| T22E16 | 77317 |
| T22E13 | 70438 |
| T25C15 | 69094 |
| T23A19 | 68001 |
| T18A14 | 62284 |
| T10A13 | 60015 |
| T18A18 | 59846 |
| T9A11 | 59741 |
| T10A11 | 58483 |
| T23A16 | 56292 |
| T10A16 | 56261 |
| T32A18 | 56193 |
| T22E17 | 55983 |
| T23A15 | 55420 |
| T9A13 | 55227 |
| T22E15 | 52674 |
| T10A15 | 51752 |
| T10A17 | 51310 |
| T10A12 | 51016 |
| T32A14 | 50305 |
| T18A19 | 49943 |
| T32A19 | 49219 |
| T25C16 | 49021 |
| T22E12 | 48800 |
| T25C12 | 48442 |
| T33A20 | 48313 |
| T18A15 | 48112 |
| T30A15 | 47526 |
| T23A11 | 46645 |
| T23A18 | 46076 |
| T33A11 | 45588 |
| T22E11 | 44744 |
| T9A15 | 44702 |
| T33A18 | 44680 |
| T1C20 | 44451 |
| T33A19 | 43993 |
| T23A12 | 43688 |
| T1C12 | 43100 |
| T5B19 | 43092 |
| T5B17 | 42817 |
| T1C18 | 42542 |
| T32A20 | 42250 |
| T33A12 | 42247 |
| T25C14 | 42220 |
| T33A15 | 42083 |
| T5B20 | 41693 |
| T23A17 | 41165 |
| T1C16 | 40397 |
| T33A13 | 40168 |
| T18A11 | 40163 |
| T33A17 | 40131 |
| T1C14 | 39654 |
| T9A12 | 39012 |
| T30A11 | 38922 |
| T1C15 | 38679 |
| T30A19 | 38407 |
| T25C17 | 38030 |
| T5B14 | 37925 |
| T23A14 | 37798 |
| T1C17 | 37700 |
| T22E19 | 37521 |
| T33A16 | 36622 |
| T30A17 | 36541 |
| T25C11 | 36393 |
| T5B15 | 36257 |
| T32A11 | 36236 |
| T30A16 | 35875 |
| T18A16 | 35708 |
| T30A18 | 35635 |
| T32A17 | 35506 |
| T25C20 | 35461 |
| T18A20 | 34562 |
| T23A13 | 34341 |
| T9A14 | 33789 |
| T5B16 | 33410 |
| T1C19 | 33281 |
| T32A13 | 32586 |
| T1C13 | 32313 |
| T10A19 | 32199 |
| T18A17 | 31857 |
| T18A13 | 31612 |
| T30A14 | 31438 |
| T30A20 | 31211 |
| T5B12 | 31080 |
| T33A14 | 29624 |
| T32A12 | 28999 |
| T5B18 | 28918 |
| T5B11 | 28222 |
| T25C18 | 27551 |
| T25C19 | 26893 |
| T32A16 | 25100 |
| T32A15 | 22852 |
| T18A12 | 20869 |
| T10A20 | 16074 |
| T5B13 | 15332 |
| T30A13 | 14104 |
| T30A12 | 7518 |
| T10A18 | 3484 |

**Table S3.** Total number of features (read count) per sample for *B. terrestris* gut microbiome (n=88) following quality filtering.

| Sample ID | Feature count |
| --- | --- |
| T21E07 | 93161 |
| T1C01 | 84934 |
| T8A07 | 71207 |
| T33A02 | 62283 |
| T1C07 | 60502 |
| T21E06 | 60475 |
| T6E06 | 59231 |
| T5B08 | 58539 |
| T25C08 | 58185 |
| T10A04 | 58142 |
| T32A02 | 57717 |
| T30A01 | 57493 |
| T10A02 | 54087 |
| T9A03 | 52239 |
| T30A06 | 51277 |
| T25C05 | 51247 |
| T21E04 | 50861 |
| T25C01 | 49647 |
| T9A05 | 49304 |
| T23A09 | 49239 |
| T33A03 | 49143 |
| T5B04 | 48926 |
| T10A06 | 48500 |
| T10A01 | 48184 |
| T33A07 | 47049 |
| T6E01 | 46934 |
| T9A02 | 46000 |
| T1C02 | 44473 |
| T23A06 | 44277 |
| T23A02 | 44170 |
| T6E03 | 43135 |
| T10A07 | 42309 |
| T33A05 | 41731 |
| T33A04 | 41568 |
| T23A05 | 41556 |
| T21E05 | 41301 |
| T6E05 | 40706 |
| T9A04 | 40111 |
| T22E02 | 39981 |
| T22E05 | 38978 |
| T30A03 | 38488 |
| T25C02 | 38125 |
| T8A08 | 37680 |
| T23A01 | 37643 |
| T1C03 | 37437 |
| T6E02 | 37189 |
| T21E02 | 36640 |
| T22E07 | 35638 |
| T5B02 | 35191 |
| T10A05 | 35132 |
| T33A01 | 35083 |
| T6E08 | 34308 |
| T9A06 | 33956 |
| T5B03 | 33401 |
| T21E00 | 32775 |
| T30A04 | 32612 |
| T5B01 | 31861 |
| T25C04 | 30985 |
| T22E03 | 30873 |
| T1C08 | 30237 |
| T1C05 | 29339 |
| T21E01 | 29235 |
| T22E04 | 29056 |
| T9A01 | 28942 |
| T9A07 | 28296 |
| T33A06 | 27036 |
| T32A03 | 27017 |
| T10A08 | 26571 |
| T22E06 | 25990 |
| T25C07 | 25842 |
| T32A01 | 23997 |
| T21E03 | 23459 |
| T23A04 | 23091 |
| T25C03 | 22495 |
| T23A03 | 22372 |
| T32A05 | 21587 |
| T5B05 | 20856 |
| T1C06 | 18091 |
| T6E07 | 17781 |
| T6E04 | 17265 |
| T8A04 | 14225 |
| T8A02 | 11781 |
| T8A09 | 11019 |
| T22E08 | 8091 |
| T33A08 | 5539 |
| T10A03 | 4740 |
| T30A05 | 3921 |
| T8A03 | 718 |

**Table S4.** Summary of sequencing read counts for *A. mellifera* pollen samples (N=7) following DADA2 pipeline.

| Sites | Input | Filtered | Denoised_forward | Denoised_reverse | Merged | Non chimeric |
| --- | --- | --- | --- | --- | --- | --- |
| T10 | 72441 | 52705 | 52561 | 52532 | 4166 | 3758 |
| T18 | 62345 | 50106 | 49890 | 49909 | 8325 | 6444 |
| T22 | 52369 | 39344 | 39141 | 39108 | 11340 | 9135 |
| T30 | 32673 | 24645 | 24544 | 24436 | 10947 | 10255 |
| T32 | 58833 | 45639 | 45493 | 45413 | 9396 | 6839 |
| T5 | 45921 | 34242 | 34132 | 34022 | 21182 | 17579 |
| T9 | 62808 | 49253 | 49099 | 49087 | 5900 | 4720 |

**Table S5.** Summary of sequencing read counts for *B. terrestris* pollen samples (N=12) following DADA2 pipeline.

| Sites | Input | Filtered | Denoised_forward | Denoised_reverse | Merged | Non chimeric |
| --- | --- | --- | --- | --- | --- | --- |
| T10 | 48139 | 34240 | 33875 | 33768 | 14798 | 12376 |
| T1 | 61491 | 50447 | 49927 | 50206 | 49280 | 39595 |
| T21 | 61288 | 48514 | 48451 | 48337 | 10283 | 7445 |
| T22 | 84338 | 66684 | 66456 | 66395 | 47544 | 43435 |
| T23 | 72234 | 58063 | 57901 | 57741 | 55738 | 41036 |
| T25 | 48308 | 38930 | 38881 | 38743 | 37755 | 28324 |
| T30 | 58026 | 44763 | 44641 | 44563 | 42376 | 39268 |
| T32 | 37577 | 25904 | 25774 | 25687 | 10201 | 9618 |
| T33 | 70444 | 53402 | 53210 | 52982 | 28486 | 18320 |
| T5 | 58388 | 46333 | 46241 | 46120 | 43132 | 41800 |
| T6 | 66854 | 52473 | 52346 | 52127 | 24108 | 20273 |

**Table S6.** Squared correlations (r^2^) of environmental variables with the site scores on NMDS axis 1 for *A. mellifera* and *B. terrestris* based on Bray-Curtis dissimilarities. Correlations that are statistically significant (p $\leq$ 0.05) are highlighted in bold for both datasets. Refer to Fig. 3 and S2 for corresponding NMDS ordination plots.

| *Environmental variables* | *Apis – NMDS envfit*  *(Bray-Curtis)* | | *Bombus – NMDS envfit*  *(Bray-Curtis)* | |
| --- | --- | --- | --- | --- |
|  | r^2^ | p | r^2^ | p |
| Mean annual temperature (AT) | **0.09** | **0.01** | 0.04 | 0.22 |
| Mean annual precipitation (AR) | 0.03 | 0.21 | **0.16** | **0.002** |
| Percentage of pasture (PP) | 0.04 | 0.12 | **0.16** | **0.001** |
| Average summer wind velocity (WV) | 0.004 | 0.75 | 0.03 | 0.28 |

**Table S7.** Pairwise PERMANOVA showing significant differences in *A. mellifera* gut bacterial communities between sites. Cells with bolded p-values indicate statistically significant differences between sites (p $\leq$ 0.05). The p-values were Bonferroni-adjusted to control for multiple comparisons.

|  | T1 | T10 | T18 | T22 | T23 | T25 | T30 | T32 | T33 | T5 |
| --- | --- | --- | --- | --- | --- | --- | --- | --- | --- | --- |
| T10 | **0.032** |  |  |  |  |  |  |  |  |  |
| T18 | **0.031** | **0.011** |  |  |  |  |  |  |  |  |
| T22 | **0.020** | **0.030** | 0.153 |  |  |  |  |  |  |  |
| T23 | 0.090 | **0.001** | 0.136 | **0.006** |  |  |  |  |  |  |
| T25 | **0.011** | **0.001** | 0.127 | **0.002** | 0.155 |  |  |  |  |  |
| T30 | 0.061 | **0.030** | 0.445 | 0.072 | **0.008** | **0.042** |  |  |  |  |
| T32 | 0.228 | 0.337 | 0.056 | 0.088 | 0.117 | **0.007** | 0.179 |  |  |  |
| T33 | 0.171 | **0.018** | 0.138 | **0.046** | **0.039** | 0.103 | 0.178 | 0.301 |  |  |
| T5 | 0.091 | 0.111 | **0.014** | **0.008** | **0.004** | **0.002** | 0.061 | 0.393 | 0.164 |  |
| T9 | 0.257 | 0.333 | 0.142 | 0.124 | **0.025** | **0.003** | 0.061 | 0.283 | 0.120 | 0.257 |

**Table S8.** Pairwise PERMANOVA showing significant differences in *B. terrestris* gut bacterial communities between sites. Cells with bolded p-values indicate statistically significant differences between sites (p $\leq$ 0.05). The p-values were Bonferroni-adjusted to control for multiple comparisons.

|  | T1 | T10 | T21 | T22 | T23 | T25 | | T30 | T32 | T33 | T5 | T6 | T8 |
| --- | --- | --- | --- | --- | --- | --- | --- | --- | --- | --- | --- | --- | --- |
| T10 | **0.003** |  |  |  |  | |  |  |  |  |  |  |  |
| T21 | **0.001** | **0.002** |  |  |  | |  |  |  |  |  |  |  |
| T22 | **0.028** | 0.531 | **0.003** |  |  | |  |  |  |  |  |  |  |
| T23 | **0.001** | **0.025** | **0.001** | 0.069 |  | |  |  |  |  |  |  |  |
| T25 | **0.016** | 0.431 | **0.001** | 0.197 | 0.116 | |  |  |  |  |  |  |  |
| T30 | **0.037** | **0.003** | **0.015** | **0.022** | **0.001** | | 0.062 |  |  |  |  |  |  |
| T32 | **0.094** | **0.021** | 0.061 | 0.086 | 0.056 | | 0.494 | 0.200 |  |  |  |  |  |
| T33 | **0.004** | **0.016** | **0.001** | 0.107 | 0.088 | | 0.630 | **0.021** | 0.566 |  |  |  |  |
| T5 | **0.006** | 0.078 | **0.001** | 0.207 | 0.293 | | 0.364 | **0.006** | 0.242 | 0.485 |  |  |  |
| T6 | **0.032** | 0.071 | **0.044** | 0.074 | **0.004** | | 0.339 | 0.094 | 0.460 | 0.080 | 0.104 |  |  |
| T8 | **0.012** | **0.002** | **0.004** | **0.024** | **0.001** | | **0.011** | 0.075 | 0.428 | **0.006** | **0.004** | 0.057 |  |
| T9 | **0.003** | **0.006** | **0.001** | **0.021** | 0.121 | | **0.024** | **0.002** | **0.006** | 0.096 | 0.499 | **0.003** | **0.001** |

**Table S9.** Summary of PCoA statistics showing the results of environmental vector fitting (envfit), including squared correlations (R²) values and permutation-based (999) P-values, for A. mellifera and B. terrestris gut microbiomes based on Jaccard and Bray-Curtis dissimilarities. Correlations that are statistically significant (p $\leq$ 0.05) are highlighted in bold for both datasets. Refer to Fig. S3 for corresponding PCoA ordination plots.

| *Environmental variables* | *PCoA envfit (Jaccard)* | | | | *PCoA envfit (Bray-Curtis)* | | | |
| --- | --- | --- | --- | --- | --- | --- | --- | --- |
|  | *Apis* | | *Bombus* | | *Apis* | | *Bombus* | |
|  | R^2^ | p | R^2^ | p | R^2^ | p | R^2^ | p |
| Mean annual temperature (AT) | **0.23** | **0.001** | 0.04 | 0.16 | 0.01 | 0.68 | 0.03 | 0.18 |
| Mean annual precipitation (AR) | **0.14** | **0.001** | **0.17** | **0.001** | 0.05 | 0.06 | 0.02 | 0.29 |
| Percentage of pasture (PP) | **0.21** | **0.001** | **0.18** | **0.002** | 0.01 | 0.61 | 0.05 | 0.06 |
| Average summer wind velocity (WV) | **0.10** | **0.02** | 0.04 | 0.23 | 0.05 | 0.08 | 0.01 | 0.72 |

**Table S10.** Summary of Tukey post hoc tests assessing the statistical significance of pairwise differences among sites in average alpha diversity (Shannon and Chao1) for gut microbiomes of *A. mellifera*. The p-values are respectively listed above and below the diagonal for the Shannon and Chao1 indices, with significant p-values (p $\leq$ 0.05) in bold. One-way ANOVA indicated that site ID was a significant predictor of diversity variation among samples for the Chao1 index (F_10,89_ = 2.44, p = 0.01), but not the Shannon index (F_10,89_ = 1.01, p = 0.44). Abbreviation: N/A = Not applicable.

|  | T1 | T10 | T18 | T22 | T23 | T25 | T30 | T32 | T33 | T5 | T9 |
| --- | --- | --- | --- | --- | --- | --- | --- | --- | --- | --- | --- |
| T1 | N/A | 1.00 | 1.00 | 1.00 | 1.00 | 0.99 | 1.00 | 0.93 | 1.00 | 1.00 | 1.00 |
| T10 | 0.97 | N/A | 1.00 | 1.00 | 1.00 | 0.81 | 0.99 | 1.00 | 1.00 | 1.00 | 1.00 |
| T18 | 0.99 | 1.00 | N/A | 1.00 | 1.00 | 0.87 | 0.99 | 1.00 | 1.00 | 1.00 | 1.00 |
| T22 | 1.00 | 1.00 | 1.00 | N/A | 1.00 | 1.00 | 1.00 | 0.81 | 1.00 | 1.00 | 0.98 |
| T23 | 1.00 | 1.00 | 1.00 | 1.00 | N/A | 0.95 | 1.00 | 0.99 | 1.00 | 1.00 | 1.00 |
| T25 | 0.71 | 0.05 | 0.08 | 0.29 | 0.37 | N/A | 1.00 | 0.28 | 0.92 | 0.87 | 0.75 |
| T30 | 0.96 | 0.23 | 0.31 | 0.65 | 0.77 | 1.00 | N/A | 0.66 | 1.00 | 0.99 | 0.95 |
| T32 | 1.00 | 0.89 | 0.94 | 1.00 | 1.00 | 0.83 | 0.99 | N/A | 0.99 | 1.00 | 1.00 |
| T33 | 1.00 | 1.00 | 1.00 | 1.00 | 1.00 | 0.46 | 0.85 | 1.00 | N/A | 1.00 | 1.00 |
| T5 | 1.00 | 0.99 | 1.00 | 1.00 | 1.00 | 0.51 | 0.88 | 1.00 | 1.00 | N/A | 1.00 |
| T9 | 0.72 | 1.00 | 1.00 | 0.98 | 0.92 | **0.03** | 0.10 | 0.55 | 0.84 | 0.81 | N/A |

**Table S11.** Summary of Tukey post hoc tests assessing the statistical significance of pairwise differences among sites in average alpha diversity (Shannon and Chao1) for gut microbiomes of *B. terrestris*. The p-values are respectively listed above and below the diagonal for the Shannon and Chao1 indices, with significant p-values (p $\leq$ 0.05) in bold. One-way ANOVA indicated that site ID was a highly significant predictor of diversity variation among samples for the Chao1 index (F_12,75_ = 7.66, p = 4.75x10^-9^), and a marginally significant predictor for the Shannon index (F_12,75_ = 1.87, p = 005). Abbreviation: N/A = Not applicable.

|  | T1 | T10 | T21 | T22 | T23 | T25 | T30 | T32 | T33 | T5 | T6 | T8 | T9 |
| --- | --- | --- | --- | --- | --- | --- | --- | --- | --- | --- | --- | --- | --- |
| T1 | N/A | 1.00 | 0.98 | 0.91 | 1.00 | 1.00 | 1.00 | 1.00 | 1.00 | 1.00 | 1.00 | 0.58 | 1.00 |
| T10 | 1.00 | N/A | 0.83 | 0.62 | 1.00 | 1.00 | 1.00 | 1.00 | 1.00 | 1.00 | 0.98 | 0.81 | 1.00 |
| T21 | 1.00 | 1.00 | N/A | 1.00 | 0.75 | 1.00 | 0.87 | 1.00 | 0.94 | 1.00 | 1.00 | **0.04** | 1.00 |
| T22 | 1.00 | 1.00 | 1.00 | N/A | 0.53 | 0.97 | 0.69 | 1.00 | 0.80 | 0.99 | 1.00 | **0.02** | 0.98 |
| T23 | 1.00 | 1.00 | 1.00 | 1.00 | N/A | 1.00 | 1.00 | 0.99 | 1.00 | 1.00 | 0.96 | 0.92 | 1.00 |
| T25 | 1.00 | 0.97 | 1.00 | 1.00 | 1.00 | N/A | 1.00 | 1.00 | 1.00 | 1.00 | 1.00 | 0.43 | 1.00 |
| T30 | 1.00 | 1.00 | 1.00 | 1.00 | 1.00 | 0.96 | N/A | 0.99 | 1.00 | 1.00 | 0.98 | 0.95 | 1.00 |
| T32 | 1.00 | 1.00 | 1.00 | 1.00 | 1.00 | 1.00 | 1.00 | N/A | 1.00 | 1.00 | 1.00 | 0.32 | 1.00 |
| T33 | 1.00 | 1.00 | 1.00 | 1.00 | 1.00 | 1.00 | 1.00 | 1.00 | N/A | 1.00 | 1.00 | 0.65 | 1.00 |
| T5 | 1.00 | 1.00 | 1.00 | 1.00 | 1.00 | 1.00 | 1.00 | 1.00 | 1.00 | N/A | 1.00 | 0.42 | 1.00 |
| T6 | 1.00 | 1.00 | 1.00 | 1.00 | 1.00 | 0.97 | 1.00 | 1.00 | 1.00 | 1.00 | N/A | 0.12 | 1.00 |
| T8 | **6.60e^-8^** | **1.45e^-7^** | **2.87e^-9^** | **4.24e^-8^** | **1.51e^-8^** | **1.12e^-9^** | **8.45e^-6^** | **3.78^-6^** | **8.86e^-9^** | **4.59e^-8^** | **1.49e^-7^** | N/A | 0.36 |
| T9 | 1 | 1.00 | 1.00 | 1.00 | 1.00 | 1.00 | 1.00 | 1.00 | 1.00 | 1.00 | 1.00 | **5.16e^-8^** | N/A |

**Table S12.** Tests used to evaluate the significance of the highest order interaction or main effect (if no interaction was present) involving bee species (two levels), pollen (Shannon) diversity, and/or environmental variables on gut bacterial (Shannon) diversity. Statistically significant (p $\leq$ 0.05) interactions are emphasised in bold. Abbreviations: Temp = Mean annual temperature, Rain = Mean annual precipitation, Wind = Average summer wind velocity, log_pasture = logit transformed values for percentage of pasture, DF = Degrees of freedom, Fac bacterial diversity = Facultative bacterial diversity. The asterisk (*) within predictor variables denotes a model with main effect and interaction.

| *Response variables* | *Predictor variables* | *DF* | *t* | *p* |
| --- | --- | --- | --- | --- |
| overall bacterial diversity | species*overall pollen diversity*log_pasture | 9.8 | -1.42 | 0.18 |
| overall bacterial diversity | species*overall pollen diversity*temp | 8.6 | -0.04 | 0.97 |
| overall bacterial diversity | species*overall pollen diversity*rain | 10.6 | 0.19 | 0.86 |
| overall bacterial diversity | species*overall pollen diversity*wind | 9.7 | 0.23 | 0.82 |
| overall bacterial diversity | species*native pollen diversity*log_pasture | 8.0 | 0.38 | 0.72 |
| overall bacterial diversity | species*native pollen diversity*temp | 9.4 | 0.36 | 0.72 |
| overall bacterial diversity | species*native pollen diversity*rain | 9.4 | 1.01 | 0.34 |
| overall bacterial diversity | species*native pollen diversity*wind | 14.1 | -1.23 | 0.24 |
| overall bacterial diversity | species*introduced pollen diversity*log_pasture | 10.2 | -0.78 | 0.45 |
| overall bacterial diversity | species*introduced pollen diversity*temp | 7.4 | -0.69 | 0.51 |
| overall bacterial diversity | species*introduced pollen diversity*rain | 9.4 | 0.22 | 0.83 |
| overall bacterial diversity | species*introduced pollen diversity*wind | 10.3 | -0.22 | 0.83 |
| overall bacterial diversity | species* ‘both’ pollen diversity*log_pasture | 11.0 | 0.74 | 0.48 |
| overall bacterial diversity | species* ‘both’ pollen diversity*temp | 7.3 | -0.35 | 0.74 |
| overall bacterial diversity | species* ‘both’ pollen diversity*rain | 9.5 | -0.46 | 0.66 |
| overall bacterial diversity | species* ‘both’ pollen diversity*wind | 9.1 | -0.6 | 0.56 |
| core bacterial diversity | species*overall pollen diversity*log_pasture | 10.3 | -0.56 | 0.59 |
| core bacterial diversity | species*overall pollen diversity*temp | 9.9 | 0.65 | 0.53 |
| core bacterial diversity | species*overall pollen diversity*rain | 10.4 | -0.95 | 0.36 |
| core bacterial diversity | species*overall pollen diversity*wind | 11.4 | 0.06 | 0.95 |
| core bacterial diversity | species*native pollen diversity*log_pasture | 9.2 | -0.04 | 0.97 |
| core bacterial diversity | species*native pollen diversity*temp | 10.7 | -0.43 | 0.67 |
| core bacterial diversity | species*native pollen diversity*rain | 9.3 | 0.084 | 0.93 |
| core bacterial diversity | species*native pollen diversity*wind | 14.5 | -1.04 | 0.31 |
| core bacterial diversity | species*introduced pollen diversity*log_pasture | 9.1 | 1.25 | 0.24 |
| core bacterial diversity | species*introduced pollen diversity*temp | 9.9 | 0.87 | 0.41 |
| core bacterial diversity | species*introduced pollen diversity*rain | 9.5 | -0.13 | 0.90 |
| core bacterial diversity | species*introduced pollen diversity*wind | 11.8 | 0.01 | 0.99 |
| core bacterial diversity | species* ‘both’ pollen diversity*log_pasture | 10.2 | 0.33 | 0.75 |
| core bacterial diversity | species* ‘both’ pollen diversity*temp | 9.0 | -0.07 | 0.95 |
| core bacterial diversity | species* ‘both’ pollen diversity*rain | 9.1 | -1.51 | 0.16 |
| core bacterial diversity | species* ‘both’ pollen diversity*wind | 11.3 | -0.67 | 0.52 |
| fac bacterial diversity | species*overall pollen diversity*log_pasture | 9.9 | -0.57 | 0.58 |
| fac bacterial diversity | species*overall pollen diversity*temp | 9.9 | -0.48 | 0.64 |
| fac bacterial diversity | species*overall pollen diversity*rain | 10.6 | 1.46 | 0.17 |
| fac bacterial diversity | species*overall pollen diversity*wind | 11.0 | 0.76 | 0.46 |
| fac bacterial diversity | species*native pollen diversity*log_pasture | 9.6 | -0.64 | 0.54 |
| fac bacterial diversity | species*native pollen diversity*temp | 10.6 | 0.87 | 0.40 |
| fac bacterial diversity | species*native pollen diversity*rain | 9.8 | 0.85 | 0.42 |
| fac bacterial diversity | species*native pollen diversity*wind | 12.6 | -0.74 | 0.47 |
| fac bacterial diversity | species*introduced pollen diversity*log_pasture | 9.7 | -1.26 | 0.24 |
| fac bacterial diversity | species*introduced pollen diversity*temp | 9.8 | -0.92 | 0.38 |
| fac bacterial diversity | species*introduced pollen diversity*rain | 9.4 | 1.3 | 0.22 |
| fac bacterial diversity | species*introduced pollen diversity*wind | 11.1 | 0.98 | 0.35 |
| fac bacterial diversity | species* ‘both’ pollen diversity*log_pasture | 10.7 | 1.004 | 0.34 |
| fac bacterial diversity | species* ‘both’ pollen diversity*temp | 9.5 | 0.36 | 0.73 |
| fac bacterial diversity | species* ‘both’ pollen diversity*rain | 10.5 | 0.61 | 0.56 |
| fac bacterial diversity | species* ‘both’ pollen diversity*wind | 11.1 | 0.07 | 0.94 |
| overall bacterial diversity | species*log_pasture | 16.0 | -0.45 | 0.66 |
| overall bacterial diversity | species*temp | 16.4 | -1.1 | 0.29 |
| overall bacterial diversity | species*rain | 17.2 | 1.86 | 0.08 |
| overall bacterial diversity | species*wind | 18.0 | -0.78 | 0.44 |
| overall bacterial diversity | species*overall pollen diversity | 10.8 | 0.71 | 0.49 |
| overall bacterial diversity | species*native pollen diversity | 13.4 | 0.028 | 0.98 |
| overall bacterial diversity | species*introduced pollen diversity | 13.0 | 1.31 | 0.21 |
| overall bacterial diversity | species* ‘both’ pollen diversity | 13.0 | 0.53 | 0.61 |
| core bacterial diversity | species*log_pasture | 15.4 | 1.21 | 0.24 |
| core bacterial diversity | species*temp | 19.3 | -1.13 | 0.27 |
| core bacterial diversity | species*rain | 16.9 | -1.27 | 0.22 |
| **core bacterial diversity** | **species*wind** | **20.2** | **-2.33** | **0.03** |
| core bacterial diversity | species*overall pollen diversity | 12.8 | 0.97 | 0.35 |
| core bacterial diversity | species*native pollen diversity | 14.5 | 1.31 | 0.21 |
| core bacterial diversity | species*introduced pollen diversity | 13.8 | 0.05 | 0.96 |
| core bacterial diversity | species* ‘both’ pollen diversity | 14.3 | 0.34 | 0.74 |
| fac bacterial diversity | species*log_pasture | 18.1 | -1.51 | 0.15 |
| fac bacterial diversity | species*temp | 19.2 | -0.64 | 0.53 |
| **fac bacterial diversity** | **species*rain** | **18.5** | **3.23** | **0.004** |
| fac bacterial diversity | species*wind | 19.8 | 0.56 | 0.58 |
| fac bacterial diversity | species*overall pollen diversity | 13.2 | -0.72 | 0.48 |
| fac bacterial diversity | species*native pollen diversity | 14.4 | -0.77 | 0.45 |
| fac bacterial diversity | species*introduced pollen diversity | 14.0 | 0.48 | 0.64 |
| fac bacterial diversity | species* ‘both’ pollen diversity | 14.5 | -0.22 | 0.83 |
| overall bacterial diversity | overall pollen diversity*temp + species | 11.9 | 0.857 | 0.41 |
| overall bacterial diversity | overall pollen diversity*rain + species | 14.5 | 0.31 | 0.76 |
| overall bacterial diversity | overall pollen diversity*wind + species | 13.5 | 0.75 | 0.46 |
| overall bacterial diversity | overall pollen diversity*log_pasture + species | 11.4 | 0.04 | 0.97 |
| overall bacterial diversity | introduced pollen diversity*temp + species | 12.4 | -1.15 | 0.27 |
| overall bacterial diversity | introduced pollen diversity*rain + species | 12.5 | 0.43 | 0.67 |
| overall bacterial diversity | introduced pollen diversity*wind + species | 15.5 | -1.85 | 0.08 |
| overall bacterial diversity | introduced pollen diversity*log_pasture + species | 13.5 | 1.74 | 0.10 |
| **overall bacterial diversity** | **native pollen diversity*temp + species** | **11.9** | **2.7** | **0.02** |
| overall bacterial diversity | native pollen diversity*rain + species | 12.9 | 0.06 | 0.95 |
| overall bacterial diversity | native pollen diversity*wind + species | 16.0 | 1.69 | 0.11 |
| overall bacterial diversity | native pollen diversity*log_pasture + species | 13.5 | 1.74 | 0.10 |
| overall bacterial diversity | ‘both’ pollen diversity*temp + species | 13.6 | 0.79 | 0.44 |
| overall bacterial diversity | ‘both’ pollen diversity*rain + species | 14.1 | 0.35 | 0.73 |
| overall bacterial diversity | ‘both’ pollen diversity*wind + species | 14.2 | 0.76 | 0.46 |
| overall bacterial diversity | ‘both’ pollen diversity*log_pasture + species | 14.9 | -1.06 | 0.31 |
| core bacterial diversity | overall pollen diversity*temp + species | 12.9 | 1.72 | 0.11 |
| core bacterial diversity | overall pollen diversity*rain + species | 15.3 | -0.95 | 0.36 |
| core bacterial diversity | overall pollen diversity*wind + species | 13.8 | 1.31 | 0.21 |
| core bacterial diversity | overall pollen diversity*log_pasture + species | 11.7 | -0.81 | 0.43 |
| core bacterial diversity | introduced pollen diversity*temp + species | 13.1 | 1.11 | 0.29 |
| core bacterial diversity | introduced pollen diversity*rain + species | 13.1 | -0.35 | 0.73 |
| core bacterial diversity | introduced pollen diversity*wind + species | 15.2 | -0.28 | 0.78 |
| core bacterial diversity | introduced pollen diversity*log_pasture + species | 11.5 | 0.17 | 0.87 |
| core bacterial diversity | native pollen diversity*temp + species | 14.4 | 0.45 | 0.66 |
| core bacterial diversity | native pollen diversity*rain + species | 14.1 | -0.6 | 0.56 |
| core bacterial diversity | native pollen diversity*wind + species | 16.4 | 0.16 | 0.88 |
| core bacterial diversity | native pollen diversity*log_pasture + species | 14.7 | -1.29 | 0.22 |
| core bacterial diversity | ‘both’ pollen diversity*temp + species | 13.8 | 1.79 | 0.10 |
| **core bacterial diversity** | **‘both’ pollen diversity*rain + species** | **15.9** | **-3.17** | **0.006** |
| core bacterial diversity | ‘both’ pollen diversity*wind + species | 14.7 | 0.24 | 0.81 |
| core bacterial diversity | ‘both’ pollen diversity*log_pasture + species | 13.3 | 1.35 | 0.2 |
| fac bacterial diversity | overall pollen diversity*temp + species | 13.4 | 0.03 | 0.98 |
| fac bacterial diversity | overall pollen diversity*rain + species | 14.4 | 0.96 | 0.35 |
| fac bacterial diversity | overall pollen diversity*wind + species | 13.8 | 0.013 | 0.99 |
| fac bacterial diversity | overall pollen diversity*log_pasture + species | 11.9 | 1.07 | 0.31 |
| fac bacterial diversity | introduced pollen diversity*temp + species | 13.4 | -0.49 | 0.63 |
| fac bacterial diversity | introduced pollen diversity*rain + species | 13.3 | 0.58 | 0.57 |
| fac bacterial diversity | introduced pollen diversity*wind + species | 14.0 | -0.4 | 0.69 |
| fac bacterial diversity | introduced pollen diversity*log_pasture + species | 11.7 | 0.85 | 0.41 |
| **fac bacterial diversity** | **native pollen diversity*temp + species** | **14.6** | **2.06** | **0.05** |
| fac bacterial diversity | native pollen diversity*rain + species | 13.7 | 0.32 | 0.76 |
| fac bacterial diversity | native pollen diversity*wind + species | 15.1 | 1.5 | 0.15 |
| **fac bacterial diversity** | **native pollen diversity*log_pasture + species** | **14.2** | **3.08** | **0.008** |
| fac bacterial diversity | ‘both’ pollen diversity*temp + species | 14.0 | -0.02 | 0.99 |
| **fac bacterial diversity** | **‘both’ pollen diversity*rain + species** | **14.3** | **3.72** | **0.002** |
| fac bacterial diversity | ‘both’ pollen diversity*wind + species | 14.0 | 0.86 | 0.40 |
| fac bacterial diversity | ‘both’ pollen diversity*log_pasture + species | 13.9 | -1.15 | 0.27 |

**Table S13.** Tests used to evaluate the significance of the highest order interaction or main effect (if no interaction was present) involving bee species (two levels), pollen (Chao1) richness, and/or environmental variables on gut bacterial (Chao1) richness. Statistically significant (p $\leq$ 0.05) interactions are emphasized in bold. Abbreviations: Temp = Mean annual temperature, Rain = Mean annual precipitation, Wind = Average summer wind velocity, log_pasture = logit transformed values for percentage of pasture, DF = Degrees of freedom, Fac bacterial richness = Facultative bacterial richness. The asterisk (*) within predictor variables denotes a model with main effect and interaction.

| *Response variables* | *Predictor variables* | *DF* | *t* | *p* |
| --- | --- | --- | --- | --- |
| overall bacterial richness | species*overall pollen richness * log_pasture | 136.0 | -0.32 | 0.75 |
| overall bacterial richness | species*overall pollen richness*temp | 8.7 | -0.006 | 0.99 |
| overall bacterial richness | species*overall pollen richness*rain | 6.5 | -1.33 | 0.23 |
| overall bacterial richness | species*overall pollen richness*wind | 8.7 | -0.37 | 0.72 |
| overall bacterial richness | species*native pollen richness* log_pasture | 136.0 | -0.52 | 0.6 |
| overall bacterial richness | species*native pollen richness*temp | 8.1 | -0.73 | 0.49 |
| overall bacterial richness | species*native pollen richness*rain | 136.0 | 1.21 | 0.23 |
| overall bacterial richness | species*native pollen richness*wind | 16.3 | 0.63 | 0.54 |
| overall bacterial richness | species*introduced pollen richness * log_pasture | 136.0 | -0.72 | 0.47 |
| overall bacterial richness | species*introduced pollen richness*temp | 6.4 | -0.96 | 0.37 |
| overall bacterial richness | species*introduced pollen richness*rain | 11.5 | -0.63 | 0.54 |
| overall bacterial richness | species*introduced pollen richness*wind | 7.8 | -0.06 | 0.96 |
| overall bacterial richness | species* ‘both’ pollen richness * log_pasture | 136.0 | 0.81 | 0.42 |
| overall bacterial richness | species* ‘both’ pollen richness*temp | 9.4 | 0.77 | 0.46 |
| overall bacterial richness | species* ‘both’ pollen richness*rain | 10.1 | 0.37 | 0.72 |
| overall bacterial richness | species* ‘both’ pollen richness*wind | 12.6 | -0.08 | 0.94 |
| core bacterial richness | species*overall pollen richness * log_pasture | 8.8 | 0.22 | 0.83 |
| core bacterial richness | species*overall pollen richness*temp | 9.6 | -1.14 | 0.28 |
| core bacterial richness | species*overall pollen richness*rain | 9.1 | -0.11 | 0.92 |
| core bacterial richness | species*overall pollen richness*wind | 9.5 | -1.36 | 0.21 |
| core bacterial richness | species*native pollen richness * log_pasture | 11.2 | 0.004 | 0.99 |
| core bacterial richness | species*native pollen richness*temp | 9.8 | -1.66 | 0.13 |
| core bacterial richness | species*native pollen richness*rain | 10.2 | 0.58 | 0.58 |
| core bacterial richness | species*native pollen richness*wind | 13.2 | -0.52 | 0.61 |
| core bacterial richness | species*introduced pollen richness * log_pasture | 9.8 | 0.27 | 0.79 |
| core bacterial richness | species*introduced pollen richness*temp | 10.4 | 0.3 | 0.77 |
| core bacterial richness | species*introduced pollen richness*rain | 11.8 | 0.15 | 0.88 |
| core bacterial richness | species*introduced pollen richness*wind | 9.1 | -1.6 | 0.15 |
| core bacterial richness | species* ‘both’ pollen richness * log_pasture | 11.6 | -0.29 | 0.78 |
| core bacterial richness | species* ‘both’ pollen richness*temp | 11.1 | -1.07 | 0.31 |
| core bacterial richness | species* ‘both’ pollen richness*rain | 10.4 | -0.71 | 0.49 |
| core bacterial richness | species* ‘both’ pollen richness*wind | 13.0 | -1.22 | 0.24 |
| fac bacterial richness | species*overall pollen richness * log_pasture | 9.5 | 0.15 | 0.88 |
| fac bacterial richness | species*overall pollen richness*temp | 9.9 | -0.26 | 0.8 |
| fac bacterial richness | species*overall pollen richness*rain | 9.2 | -0.63 | 0.55 |
| fac bacterial richness | species*overall pollen richness*wind | 9.6 | -0.53 | 0.61 |
| fac bacterial richness | species*native pollen richness * log_pasture | 11.5 | -0.58 | 0.57 |
| fac bacterial richness | species*native pollen richness*temp | 10.2 | -0.14 | 0.89 |
| fac bacterial richness | species*native pollen richness*rain | 9.6 | 0.33 | 0.75 |
| fac bacterial richness | species*native pollen richness*wind | 11.2 | 0.49 | 0.63 |
| fac bacterial richness | species*introduced pollen richness * log_pasture | 10.2 | -0.22 | 0.83 |
| fac bacterial richness | species*introduced pollen richness*temp | 10.2 | -0.46 | 0.65 |
| fac bacterial richness | species*introduced pollen richness*rain | 10.7 | 0.22 | 0.83 |
| fac bacterial richness | species*introduced pollen richness*wind | 10.0 | 0.38 | 0.71 |
| fac bacterial richness | species*introduced pollen richness * log_pasture | 11.3 | 0.16 | 0.88 |
| fac bacterial richness | species* ‘both’ pollen richness*temp | 10.4 | 0.41 | 0.69 |
| fac bacterial richness | species* ‘both’ pollen richness*rain | 10.5 | 0.25 | 0.81 |
| fac bacterial richness | species* ‘both’ pollen richness*wind | 11.1 | 0.22 | 0.83 |
| overall bacterial richness | species*log_pasture | 18.6 | -1.2 | 0.24 |
| overall bacterial richness | species*temp | 19.2 | 0.09 | 0.93 |
| **overall bacterial richness** | **species*rain** | **18.4** | **2.78** | **0.01** |
| overall bacterial richness | species*wind | 19.4 | 0.64 | 0.53 |
| overall bacterial richness | species*overall pollen richness | 140.0 | -0.92 | 0.36 |
| overall bacterial richness | species*native pollen richness | 140.0 | -0.01 | 0.99 |
| overall bacterial richness | species*introduced pollen richness | 140.0 | -1.3 | 0.21 |
| overall bacterial richness | species*'both' pollen richness | 14.0 | 0.07 | 0.95 |
| core bacterial richness | species*log_pasture | 18.5 | 1.32 | 0.2 |
| core bacterial richness | species*temp | 20.7 | -0.16 | 0.87 |
| core bacterial richness | species*rain | 18.9 | -1.14 | 0.27 |
| core bacterial richness | species*wind | 21.5 | -1.47 | 0.16 |
| core bacterial richness | species*overall pollen richness | 12.6 | 0.18 | 0.86 |
| core bacterial richness | species*native pollen richness | 13.4 | 0.91 | 0.38 |
| core bacterial richness | species*introduced pollen richness | 15.0 | -0.02 | 0.99 |
| core bacterial richness | species* ‘both’ pollen richness | 15.1 | 0.23 | 0.82 |
| fac bacterial richness | species*log_pasture | 18.8 | -1.3 | 0.21 |
| fac bacterial richness | species*temp | 19.5 | -0.25 | 0.81 |
| **fac bacterial richness** | **species*rain** | **18.7** | **2.31** | **0.03** |
| fac bacterial richness | species*wind | 19.8 | 0.68 | 0.5 |
| fac bacterial richness | species*overall pollen richness | 12.6 | -0.96 | 0.36 |
| fac bacterial richness | species*native pollen richness | 13.8 | -0.61 | 0.56 |
| fac bacterial richness | species*introduced pollen richness | 14.1 | -0.67 | 0.52 |
| fac bacterial richness | species* ‘both’pollen richness | 14.4 | 0.18 | 0.86 |
| overall bacterial richness | overall pollen richness*temp + species | 14.9 | 0.81 | 0.43 |
| overall bacterial richness | overall pollen richness*rain + species | 13.3 | 0.3 | 0.77 |
| overall bacterial richness | overall pollen richness*wind + species | 14.9 | 0.27 | 0.79 |
| overall bacterial richness | overall pollen richness*log_pasture + species | 139.0 | 1.59 | 0.11 |
| overall bacterial richness | introduced pollen richness*temp + species | 139.0 | 1.58 | 0.12 |
| overall bacterial richness | introduced pollen richness*rain + species | 13.6 | 0.87 | 0.4 |
| overall bacterial richness | introduced pollen richness*wind + species | 139.0 | 1.72 | 0.09 |
| overall bacterial richness | introduced pollen richness*log_pasture + species | 139.0 | 1.22 | 0.23 |
| overall bacterial richness | native pollen richness*temp + species | 16.3 | -0.21 | 0.84 |
| overall bacterial richness | native pollen richness*rain + species | 139.0 | -1.85 | 0.06 |
| overall bacterial richness | native pollen richness*wind + species | 139.0 | -1.26 | 0.21 |
| **overall bacterial richness** | **native pollen richness*log_pasture + species** | **139.0** | **1.99** | **0.05** |
| overall bacterial richness | ‘both’ pollen richness*temp + species | 14.9 | -0.63 | 0.54 |
| overall bacterial richness | ‘both’ pollen richness*rain + species | 13.1 | 0.44 | 0.67 |
| overall bacterial richness | ‘both’ pollen richness*wind + species | 14.0 | -0.61 | 0.55 |
| overall bacterial richness | ‘both’ pollen richness*log_pasture + species | 15.2 | 0.04 | 0.97 |
| core bacterial richness | overall pollen richness*temp + species | 15.1 | -0.03 | 0.98 |
| core bacterial richness | overall pollen richness*rain + species | 14.5 | 0.48 | 0.64 |
| core bacterial richness | overall pollen richness*wind + species | 15.9 | -0.1 | 0.92 |
| core bacterial richness | overall pollen richness*log_pasture + species | 13.7 | 0.16 | 0.87 |
| core bacterial richness | introduced pollen richness*temp + species | 16.2 | 0.57 | 0.58 |
| core bacterial richness | introduced pollen richness*rain + species | 14.6 | -1.2 | 0.25 |
| core bacterial richness | introduced pollen richness*wind + species | 15.8 | -0.6 | 0.56 |
| core bacterial richness | introduced pollen richness*log_pasture + species | 14.2 | -0.6 | 0.56 |
| core bacterial richness | native pollen richness*temp + species | 17.1 | -0.56 | 0.59 |
| core bacterial richness | native pollen richness*rain + species | 18.1 | -0.03 | 0.97 |
| core bacterial richness | native pollen richness*wind + species | 17.2 | -0.39 | 0.7 |
| core bacterial richness | native pollen richness*log_pasture + species | 14.5 | -0.76 | 0.46 |
| core bacterial richness | ‘both’ pollen richness*temp + species | 15.1 | 0.05 | 0.96 |
| core bacterial richness | ‘both’ pollen richness*rain + species | 14.5 | 0.22 | 0.83 |
| core bacterial richness | ‘both’ pollen richness*wind + species | 14.5 | -0.04 | 0.97 |
| core bacterial richness | ‘both’ pollen richness*log_pasture + species | 15.1 | 1.29 | 0.22 |
| fac bacterial richness | overall pollen richness*temp + species | 13.5 | 0.29 | 0.78 |
| fac bacterial richness | overall pollen richness*rain + species | 12.8 | 0.38 | 0.71 |
| fac bacterial richness | overall pollen richness*wind + species | 13.1 | -0.64 | 0.53 |
| **fac bacterial richness** | **overall pollen richness*log_pasture + species** | **13.0** | **2.28** | **0.04** |
| fac bacterial richness | introduced pollen richness*temp + species | 14.7 | 0.72 | 0.48 |
| fac bacterial richness | introduced pollen richness*rain + species | 13.7 | 0.72 | 0.49 |
| fac bacterial richness | introduced pollen richness*wind + species | 14.2 | 1.28 | 0.22 |
| fac bacterial richness | introduced pollen richness*log_pasture + species | 13.9 | 1.53 | 0.15 |
| fac bacterial richness | native pollen richness*temp + species | 14.8 | 0.45 | 0.66 |
| fac bacterial richness | native pollen richness*rain + species | 14.9 | -1.98 | 0.06 |
| fac bacterial richness | native pollen richness*wind + species | 14.6 | -0.38 | 0.71 |
| **fac bacterial richness** | **native pollen richness*log_pasture + species** | **14.3** | **2.92** | **0.01** |
| fac bacterial richness | ‘both’ pollen richness*temp + species | 14.0 | -0.58 | 0.57 |
| fac bacterial richness | ‘both’ pollen richness*rain + species | 13.7 | 1.26 | 0.23 |
| fac bacterial richness | ‘both’ pollen richness*wind + species | 13.6 | 0.06 | 0.96 |
| fac bacterial richness | ‘both’ pollen richness*log_pasture + species | 14.2 | -0.93 | 0.37 |

**Table S14.** Tests used to evaluate the significance of the highest order interaction or main effect (if no interaction was present) involving *B. terrestris,* pollen (Shannon) diversity, and/or environmental variables on gut bacterial (Shannon) diversity. Statistically significant (p $\leq$ 0.05) interactions are highlighted in bold. Abbreviations: Temp = Mean annual temperature, Rain = Mean annual precipitation, Wind = Average summer wind velocity, log_pasture = logit transformed values for percentage of pasture, DF = Degrees of freedom, Fac bacterial diversity = Facultative bacterial diversity. The asterisk (*) within predictor variables denotes a model with main effect and interaction.

| *Response variables* | *Predictor variables* | *DF* | *t* | *p* |
| --- | --- | --- | --- | --- |
| overall bacterial diversity | overall pollen diversity | 10 | 1.43 | 0.18 |
| overall bacterial diversity | native pollen diversity | 10 | 0.52 | 0.61 |
| overall bacterial diversity | ‘both’ pollen diversity | 10 | 0.94 | 0.37 |
| overall bacterial diversity | introduced pollen diversity | 10 | 1.61 | 0.14 |
| core bacterial diversity | overall pollen diversity | 10 | 0.85 | 0.41 |
| core bacterial diversity | native pollen diversity | 10 | 1.44 | 0.18 |
| core bacterial diversity | ‘both’ pollen diversity | 10 | 1.05 | 0.32 |
| core bacterial diversity | introduced pollen diversity | 10 | -0.32 | 0.76 |
| fac bacterial diversity | overall pollen diversity | 10 | 0.03 | 0.98 |
| fac bacterial diversity | native pollen diversity | 10 | -0.38 | 0.71 |
| fac bacterial diversity | ‘both’ pollen diversity | 10 | -0.67 | 0.52 |
| fac bacterial diversity | introduced pollen diversity | 10 | 1.07 | 0.31 |
| overall bacterial diversity | Temp | 11 | -0.48 | 0.64 |
| **overall bacterial diversity** | **Rain** | **11** | **2.08** | **0.05** |
| overall bacterial diversity | Wind | 11 | -2.38 | 0.51 |
| overall bacterial diversity | log_pasture | 11 | -0.07 | 0.95 |
| core bacterial diversity | Temp | 11 | -0.91 | 0.38 |
| core bacterial diversity | Rain | 11 | -1.23 | 0.24 |
| **core bacterial diversity** | **Wind** | **11** | **-2.21** | **0.04** |
| core bacterial diversity | log_pasture | 11 | 1.60 | 0.14 |
| fac bacterial diversity | Temp | 11 | 0.11 | 0.92 |
| **fac bacterial diversity** | **Rain** | **11** | **2.68** | **0.02** |
| fac bacterial diversity | Wind | 11 | 0.38 | 0.71 |
| fac bacterial diversity | log_pasture | 11 | -1.56 | 0.15 |
| overall bacterial diversity | Rain*Temp | 9 | -0.37 | 0.72 |
| overall bacterial diversity | Rain*log_pasture | 9 | -1.79 | 0.11 |
| overall bacterial diversity | Rain*Wind | 9 | 1.02 | 0.33 |
| overall bacterial diversity | Temp*log_pasture | 9 | -0.52 | 0.62 |
| **overall bacterial diversity** | **Wind*Temp** | **9** | **-3.63** | **0.006** |
| overall bacterial diversity | Wind*log_pasture | 9 | -0.73 | 0.48 |
| core bacterial diversity | Rain*Temp | 9 | 1.11 | 0.3 |
| core bacterial diversity | Rain*log_pasture | 9 | -1.42 | 0.2 |
| core bacterial diversity | Rain*Wind | 9 | 2.09 | 0.06 |
| core bacterial diversity | Temp*log_pasture | 9 | 0.61 | 0.56 |
| core bacterial diversity | Wind*Temp | 9 | 0.54 | 0.6 |
| core bacterial diversity | Wind*log_pasture | 9 | -0.02 | 0.99 |
| fac bacterial diversity | Rain*Temp | 9 | -1.03 | 0.33 |
| fac bacterial diversity | Rain*log_pasture | 9 | -1.03 | 0.33 |
| fac bacterial diversity | Rain*Wind | 9 | -0.69 | 0.51 |
| fac bacterial diversity | Temp*log_pasture | 9 | -0.51 | 0.62 |
| fac bacterial diversity | Wind*Temp | 9 | -1.96 | 0.08 |
| fac bacterial diversity | Wind*log_pasture | 9 | -0.61 | 0.56 |
| overall bacterial diversity | overall pollen diversity*Temp | 8 | 0.07 | 0.95 |
| overall bacterial diversity | overall pollen diversity*Rain | 8 | 0.46 | 0.66 |
| overall bacterial diversity | overall pollen diversity*Wind | 8 | -0.23 | 0.83 |
| overall bacterial diversity | overall pollen diversity*log_pasture | 8 | -0.95 | 0.37 |
| overall bacterial diversity | native pollen diversity*Temp | 8 | 1.67 | 0.13 |
| overall bacterial diversity | native pollen diversity*Rain | 8 | 0.75 | 0.47 |
| overall bacterial diversity | native pollen diversity*Wind | 8 | 0.49 | 0.63 |
| overall bacterial diversity | native pollen diversity*log_pasture | 8 | 1.92 | 0.09 |
| overall bacterial diversity | ‘both’ pollen diversity*Temp | 8 | 0.17 | 0.87 |
| overall bacterial diversity | ‘both’ pollen diversity*Rain | 8 | 0.05 | 0.96 |
| overall bacterial diversity | ‘both’ pollen diversity*Wind | 8 | -0.15 | 0.88 |
| overall bacterial diversity | ‘both’ pollen diversity*log_pasture | 8 | -0.78 | 0.46 |
| overall bacterial diversity | introduced pollen diversity*Temp | 8 | -0.93 | 0.38 |
| overall bacterial diversity | introduced pollen diversity*Rain | 8 | -0.08 | 0.94 |
| overall bacterial diversity | introduced pollen diversity*Wind | 8 | -1.69 | 0.13 |
| overall bacterial diversity | introduced pollen diversity*log_pasture | 8 | -0.47 | 0.65 |
| core bacterial diversity | overall pollen diversity*Temp | 8 | 1.28 | 0.24 |
| **core bacterial diversity** | **overall pollen diversity*Rain** | **8** | **-2.84** | **0.02** |
| core bacterial diversity | overall pollen diversity*Wind | 8 | -0.13 | 0.9 |
| core bacterial diversity | overall pollen diversity*log_pasture | 8 | -0.46 | 0.66 |
| core bacterial diversity | native pollen diversity*Temp | 8 | -0.17 | 0.87 |
| core bacterial diversity | native pollen diversity*Rain | 8 | -0.17 | 0.87 |
| core bacterial diversity | native pollen diversity*Wind | 8 | -1.21 | 0.26 |
| core bacterial diversity | native pollen diversity*log_pasture | 8 | -0.53 | 0.61 |
| core bacterial diversity | ‘both’ pollen diversity*Temp | 8 | 1.23 | 0.25 |
| **core bacterial diversity** | ‘both’ **pollen diversity*Rain** | **8** | **-2.98** | **0.01** |
| core bacterial diversity | ‘both’ pollen diversity*Wind | 8 | -1.06 | 0.32 |
| core bacterial diversity | ‘both’ pollen diversity*log_pasture | 8 | 1.13 | 0.29 |
| core bacterial diversity | introduced pollen diversity*Temp | 8 | 1.18 | 0.27 |
| core bacterial diversity | introduced pollen diversity*Rain | 8 | -0.66 | 0.53 |
| core bacterial diversity | introduced pollen diversity*Wind | 8 | -0.42 | 0.68 |
| core bacterial diversity | introduced pollen diversity*log_pasture | 8 | 1.29 | 0.23 |
| fac bacterial diversity | overall pollen diversity*Temp | 8 | -0.72 | 0.49 |
| **fac bacterial diversity** | **overall pollen diversity*Rain** | **8** | **3.81** | **0.005** |
| fac bacterial diversity | overall pollen diversity*Wind | 8 | 0.33 | 0.75 |
| fac bacterial diversity | overall pollen diversity*log_pasture | 8 | 0.11 | 0.92 |
| fac bacterial diversity | native pollen diversity*Temp | 8 | 1.83 | 0.1 |
| fac bacterial diversity | native pollen diversity*Rain | 8 | 0.91 | 0.39 |
| fac bacterial diversity | native pollen diversity*Wind | 8 | 1.51 | 0.17 |
| **fac bacterial diversity** | **native pollen diversity*log_pasture** | **8** | **2.30** | **0.05** |
| fac bacterial diversity | ‘both’ pollen diversity*Temp | 8 | -0.44 | 0.67 |
| **fac bacterial diversity** | ‘both’ **pollen diversity*Rain** | **8** | **3.22** | **0.01** |
| fac bacterial diversity | ‘both’ pollen diversity*Wind | 8 | 0.82 | 0.44 |
| fac bacterial diversity | ‘both’ pollen diversity*log_pasture | 8 | -0.89 | 0.4 |
| fac bacterial diversity | introduced pollen diversity*Temp | 8 | -0.70 | 0.5 |
| fac bacterial diversity | introduced pollen diversity*Rain | 8 | 1.28 | 0.23 |
| fac bacterial diversity | introduced pollen diversity*Wind | 8 | 0.24 | 0.81 |
| fac bacterial diversity | introduced pollen diversity*log_pasture | 8 | -0.92 | 0.39 |

**Table S15.** Tests used to evaluate the significance of the highest order interaction or main effect (if no interaction was present) involving *B. terrestris,* pollen (Chao1) richness, and/or environmental variables on gut bacterial (Chao1) richness. Statistically significant (p $\leq$ 0.05) interactions are highlighted in bold. Abbreviations: Temp = Mean annual temperature, Rain = Mean annual precipitation, Wind = Average summer wind velocity, log_pasture = logit transformed values for percentage of pasture, DF = Degrees of freedom, Fac bacterial richness = facultative bacterial richness. The asterisk (*) within predictor variables denotes a model with main effect and interaction.

| *Response variables* | *Predictor variables* | *DF* | *t* | *p* |
| --- | --- | --- | --- | --- |
| overall bacterial richness | overall pollen richness | 10 | 0.44 | 0.67 |
| overall bacterial richness | native pollen richness | 10 | 0.69 | 0.51 |
| overall bacterial richness | ‘both’ pollen richness | 10 | -0.02 | 0.98 |
| overall bacterial richness | introduced pollen richness | 10 | -0.4 | 0.7 |
| core bacterial richness | overall pollen richness | 10 | -2.12 | 0.06 |
| core bacterial richness | native pollen richness | 10 | -0.57 | 0.58 |
| core bacterial richness | ‘both’ pollen richness | 10 | -0.16 | 0.87 |
| core bacterial richness | introduced pollen richness | 10 | -0.38 | 0.72 |
| fac bacterial richness | overall pollen richness | 10 | 1.56 | 0.15 |
| fac bacterial richness | native pollen richness | 10 | -0.02 | 0.98 |
| fac bacterial richness | ‘both’ pollen richness | 10 | -0.38 | 0.71 |
| fac bacterial richness | introduced pollen richness | 10 | 1.41 | 0.19 |
| overall bacterial richness | Temp | 11 | 0.04 | 0.97 |
| **overall bacterial richness** | **Rain** | **11** | **2.98** | **0.01** |
| overall bacterial richness | Wind | 11 | -1.13 | 0.28 |
| overall bacterial richness | log_pasture | 11 | 0.4 | 0.7 |
| core bacterial richness | Temp | 11 | -0.17 | 0.87 |
| core bacterial richness | Rain | 11 | -0.25 | 0.81 |
| core bacterial richness | Wind | 11 | -0.81 | 0.44 |
| core bacterial richness | log_pasture | 11 | 1.67 | 0.12 |
| fac bacterial richness | Temp | 11 | 0.07 | 0.94 |
| **fac bacterial richness** | **Rain** | **11** | **2.17** | **0.05** |
| fac bacterial richness | Wind | 11 | 0.09 | 0.93 |
| fac bacterial richness | log_pasture | 11 | -0.88 | 0.4 |
| overall bacterial richness | Rain*Temp | 9 | 1 | 0.34 |
| **overall bacterial richness** | **Rain*log_pasture** | **9** | **-4.68** | **0.001** |
| overall bacterial richness | Rain*Wind | 9 | 1.61 | 0.14 |
| overall bacterial richness | Temp*log_pasture | 9 | 0.23 | 0.82 |
| overall bacterial richness | Wind* Temp | 9 | -1.06 | 0.32 |
| overall bacterial richness | Wind*log_pasture | 9 | -0.32 | 0.76 |
| core bacterial richness | Rain*Temp | 9 | 1.56 | 0.15 |
| core bacterial richness | Rain*log_pasture | 9 | -1.39 | 0.2 |
| **core bacterial richness** | **Rain*Wind** | **9** | **2.28** | **0.04** |
| core bacterial richness | Temp*log_pasture | 9 | 0.37 | 0.72 |
| core bacterial richness | Wind* Temp | 9 | -0.04 | 0.96 |
| core bacterial richness | Wind*log_pasture | 9 | 0.09 | 0.93 |
| fac bacterial richness | Rain*Temp | 9 | -1.21 | 0.26 |
| fac bacterial richness | Rain*log_pasture | 9 | -1.13 | 0.29 |
| fac bacterial richness | Rain*Wind | 9 | -0.23 | 0.82 |
| fac bacterial richness | Temp*log_pasture | 9 | 0.44 | 0.67 |
| fac bacterial richness | Wind* Temp | 9 | -1.26 | 0.24 |
| fac bacterial richness | Wind*log_pasture | 9 | -0.03 | 0.97 |
| overall bacterial richness | overall pollen richness*Temp | 8 | 0.58 | 0.58 |
| overall bacterial richness | overall pollen richness*Rain | 8 | -0.05 | 0.96 |
| overall bacterial richness | overall pollen richness*Wind | 8 | 0.32 | 0.76 |
| overall bacterial richness | overall pollen richness*log_pasture | 8 | 0.84 | 0.42 |
| overall bacterial richness | native pollen richness*Temp | 8 | -0.33 | 0.75 |
| overall bacterial richness | native pollen richness*Rain | 8 | -0.73 | 0.49 |
| overall bacterial richness | native pollen richness*Wind | 8 | -1.07 | 0.32 |
| overall bacterial richness | native pollen richness*log_pasture | 8 | 1.41 | 0.2 |
| overall bacterial richness | ‘both’ pollen richness*Temp | 8 | 0.09 | 0.93 |
| overall bacterial richness | ‘both’ pollen richness*Rain | 8 | 0.04 | 0.97 |
| overall bacterial richness | ‘both’ pollen richness*Wind | 8 | -0.12 | 0.91 |
| overall bacterial richness | ‘both’ pollen richness*log_pasture | 8 | 0.43 | 0.68 |
| overall bacterial richness | introduced pollen richness*Temp | 8 | 0.72 | 0.49 |
| overall bacterial richness | introduced pollen richness*Rain | 8 | 0.58 | 0.58 |
| overall bacterial richness | introduced pollen richness*Wind | 8 | 1.14 | 0.29 |
| overall bacterial richness | introduced pollen richness*log_pasture | 8 | 0.42 | 0.69 |
| core bacterial richness | overall pollen richness*Temp | 8 | -0.22 | 0.83 |
| core bacterial richness | overall pollen richness*Rain | 8 | 0.99 | 0.35 |
| core bacterial richness | overall pollen richness*Wind | 8 | -0.35 | 0.74 |
| core bacterial richness | overall pollen richness*log_pasture | 8 | 0.07 | 0.94 |
| core bacterial richness | native pollen richness*Temp | 8 | -1.29 | 0.23 |
| core bacterial richness | native pollen richness*Rain | 8 | 0.22 | 0.83 |
| core bacterial richness | native pollen richness*Wind | 8 | -0.52 | 0.62 |
| core bacterial richness | native pollen richness*log_pasture | 8 | -0.2 | 0.85 |
| core bacterial richness | ‘both’ pollen richness*Temp | 8 | -0.68 | 0.51 |
| core bacterial richness | ‘both’ pollen richness*Rain | 8 | 0.26 | 0.8 |
| core bacterial richness | ‘both’ pollen richness*Wind | 8 | -1.79 | 0.11 |
| core bacterial richness | ‘both’ pollen richness*log_pasture | 8 | 1.67 | 0.13 |
| core bacterial richness | introduced pollen richness*Temp | 8 | 0.63 | 0.54 |
| core bacterial richness | introduced pollen richness*Rain | 8 | -0.42 | 0.69 |
| core bacterial richness | introduced pollen richness*Wind | 8 | -0.55 | 0.6 |
| core bacterial richness | introduced pollen richness*log_pasture | 8 | -0.34 | 0.74 |
| fac bacterial richness | overall pollen richness*Temp | 8 | 0.1 | 0.92 |
| fac bacterial richness | overall pollen richness*Rain | 8 | 0.11 | 0.91 |
| fac bacterial richness | overall pollen richness*Wind | 8 | -0.44 | 0.67 |
| fac bacterial richness | overall pollen richness*log_pasture | 8 | 1.6 | 0.15 |
| fac bacterial richness | native pollen richness*Temp | 8 | 0.62 | 0.56 |
| fac bacterial richness | native pollen richness*Rain | 8 | -1.53 | 0.16 |
| fac bacterial richness | native pollen richness*Wind | 8 | -0.15 | 0.89 |
| fac bacterial richness | native pollen richness*log_pasture | 8 | 1.99 | 0.08 |
| fac bacterial richness | ‘both’ pollen richness*Temp | 8 | -0.22 | 0.83 |
| fac bacterial richness | ‘both’ pollen richness*Rain | 8 | 0.56 | 0.59 |
| fac bacterial richness | ‘both’ pollen richness*Wind | 8 | 0.29 | 0.78 |
| fac bacterial richness | ‘both’ pollen richness*log_pasture | 8 | -0.9 | 0.4 |
| fac bacterial richness | introduced pollen richness*Temp | 8 | 0.41 | 0.69 |
| fac bacterial richness | introduced pollen richness*Rain | 8 | 0.34 | 0.74 |
| fac bacterial richness | introduced pollen richness*Wind | 8 | 0.93 | 0.38 |
| fac bacterial richness | introduced pollen richness*log_pasture | 8 | 0.91 | 0.39 |

**Table S16.** Tests used to evaluate the significance of the highest order interaction or main effect (if no interaction was present) involving *A. mellifera,* pollen (Shannon) diversity, and/or environmental variables on gut bacterial (Shannon) diversity. All interactions were statistically insignificant (p > 0.05). Abbreviations: Temp = Mean annual temperature, Rain = Mean annual precipitation, Wind = Average summer wind velocity, log_pasture = logit transformed values for percentage of pasture, DF = Degrees of freedom, Fac bacterial diversity = facultative bacterial diversity. The asterisk (*) within predictor variables denotes a model with main effect and interaction.

| *Response variables* | *Predictor variables* | *DF* | *t* | *p* |
| --- | --- | --- | --- | --- |
| overall bacterial diversity | overall pollen diversity | 5 | -0.03 | 0.98 |
| overall bacterial diversity | native pollen diversity | 5 | 0.60 | 0.57 |
| overall bacterial diversity | ‘both’ pollen diversity | 5 | -0.29 | 0.78 |
| overall bacterial diversity | introduced pollen diversity | 5 | -0.19 | 0.86 |
| core bacterial diversity | overall pollen diversity | 5 | -1.16 | 0.30 |
| core bacterial diversity | native pollen diversity | 5 | -0.61 | 0.57 |
| core bacterial diversity | ‘both’ pollen diversity | 5 | 0.01 | 0.99 |
| core bacterial diversity | introduced pollen diversity | 5 | -0.77 | 0.48 |
| fac bacterial diversity | overall pollen diversity | 5 | 1.14 | 0.31 |
| fac bacterial diversity | native pollen diversity | 5 | 0.85 | 0.43 |
| fac bacterial diversity | ‘both’ pollen diversity | 5 | -0.01 | 0.99 |
| fac bacterial diversity | introduced pollen diversity | 5 | 0.48 | 0.65 |
| overall bacterial diversity | Temp | 9 | 1.47 | 0.23 |
| overall bacterial diversity | Rain | 9 | -0.36 | 0.72 |
| overall bacterial diversity | Wind | 9 | 0.46 | 0.65 |
| overall bacterial diversity | log_pasture | 9 | 0.90 | 0.39 |
| core bacterial diversity | Temp | 9 | 0.72 | 0.49 |
| core bacterial diversity | Rain | 9 | 0.42 | 0.68 |
| core bacterial diversity | Wind | 9 | 0.43 | 0.69 |
| core bacterial diversity | log_pasture | 9 | 0.71 | 0.49 |
| fac bacterial diversity | Temp | 9 | 1.45 | 0.18 |
| fac bacterial diversity | Rain | 9 | -2.07 | 0.07 |
| fac bacterial diversity | Wind | 9 | -0.45 | 0.66 |
| fac bacterial diversity | log_pasture | 9 | 0.52 | 0.61 |
| overall bacterial diversity | Rain*Temp | 7 | -0.24 | 0.82 |
| overall bacterial diversity | Rain*log_pasture | 7 | 1.06 | 0.33 |
| overall bacterial diversity | Rain*Wind | 7 | -1.39 | 0.21 |
| overall bacterial diversity | Temp*log_pasture | 7 | -0.48 | 0.65 |
| overall bacterial diversity | Wind* Temp | 7 | -0.42 | 0.69 |
| overall bacterial diversity | Wind*log_pasture | 7 | -0.16 | 0.88 |
| core bacterial diversity | Rain*Temp | 7 | -0.52 | 0.62 |
| core bacterial diversity | Rain*log_pasture | 7 | -0.54 | 0.60 |
| core bacterial diversity | Rain*Wind | 7 | 0.13 | 0.90 |
| core bacterial diversity | Temp*log_pasture | 7 | -0.17 | 0.87 |
| core bacterial diversity | Wind* Temp | 7 | -0.91 | 0.39 |
| core bacterial diversity | Wind*log_pasture | 7 | -0.02 | 0.98 |
| fac bacterial diversity | Rain*Temp | 7 | 0.38 | 0.72 |
| fac bacterial diversity | Rain*log_pasture | 7 | 1.70 | 0.15 |
| fac bacterial diversity | Rain*Wind | 7 | -1.76 | 0.12 |
| fac bacterial diversity | Temp*log_pasture | 7 | -0.44 | 0.68 |
| fac bacterial diversity | Wind* Temp | 7 | -0.69 | 0.51 |
| fac bacterial diversity | Wind*log_pasture | 7 | -0.43 | 0.68 |
| overall bacterial diversity | overall pollen diversity*Temp | 3 | 0.08 | 0.94 |
| overall bacterial diversity | overall pollen diversity*Rain | 3 | -0.04 | 0.97 |
| overall bacterial diversity | overall pollen diversity*Wind | 3 | -0.31 | 0.78 |
| overall bacterial diversity | overall pollen diversity*log_pasture | 3 | 1.37 | 0.26 |
| overall bacterial diversity | native pollen diversity*Temp | 3 | 1.29 | 0.29 |
| overall bacterial diversity | native pollen diversity*Rain | 3 | -0.78 | 0.49 |
| overall bacterial diversity | native pollen diversity*Wind | 3 | 1.72 | 0.18 |
| overall bacterial diversity | native pollen diversity*log_pasture | 3 | 0.31 | 0.78 |
| overall bacterial diversity | ‘both’ pollen diversity*Temp | 3 | 0.62 | 0.58 |
| overall bacterial diversity | ‘both’ pollen diversity*Rain | 3 | 0.70 | 0.53 |
| overall bacterial diversity | ‘both’ pollen diversity*Wind | 3 | 0.64 | 0.57 |
| overall bacterial diversity | ‘both’ pollen diversity*log_pasture | 3 | -1.17 | 0.33 |
| overall bacterial diversity | introduced pollen diversity*Temp | 3 | -0.07 | 0.95 |
| overall bacterial diversity | introduced pollen diversity*Rain | 3 | -0.42 | 0.70 |
| overall bacterial diversity | introduced pollen diversity*Wind | 3 | 0.84 | 0.46 |
| overall bacterial diversity | introduced pollen diversity*log_pasture | 3 | 0.77 | 0.49 |
| core bacterial diversity | overall pollen diversity*Temp | 3 | 0.09 | 0.94 |
| core bacterial diversity | overall pollen diversity*Rain | 3 | -0.12 | 0.91 |
| core bacterial diversity | overall pollen diversity*Wind | 3 | -0.04 | 0.97 |
| core bacterial diversity | overall pollen diversity*log_pasture | 3 | 0.68 | 0.55 |
| core bacterial diversity | native pollen diversity*Temp | 3 | 0.82 | 0.47 |
| core bacterial diversity | native pollen diversity*Rain | 3 | -0.52 | 0.64 |
| core bacterial diversity | native pollen diversity*Wind | 3 | 1.27 | 0.29 |
| core bacterial diversity | native pollen diversity*log_pasture | 3 | -0.22 | 0.84 |
| core bacterial diversity | ‘both’ pollen diversity*Temp | 3 | 1.26 | 0.30 |
| core bacterial diversity | ‘both’ pollen diversity*Rain | 3 | 0.74 | 0.51 |
| core bacterial diversity | ‘both’ pollen diversity*Wind | 3 | 0.77 | 0.50 |
| core bacterial diversity | ‘both’ pollen diversity*log_pasture | 3 | -0.02 | 0.99 |
| core bacterial diversity | introduced pollen diversity*Temp | 3 | 0.08 | 0.94 |
| core bacterial diversity | introduced pollen diversity*Rain | 3 | -1.09 | 0.35 |
| core bacterial diversity | introduced pollen diversity*Wind | 3 | -0.39 | 0.72 |
| core bacterial diversity | introduced pollen diversity*log_pasture | 3 | -0.33 | 0.76 |
| fac bacterial diversity | overall pollen diversity*Temp | 3 | 0.21 | 0.85 |
| fac bacterial diversity | overall pollen diversity*Rain | 3 | -0.27 | 0.80 |
| fac bacterial diversity | overall pollen diversity*Wind | 3 | -0.90 | 0.44 |
| fac bacterial diversity | overall pollen diversity*log_pasture | 3 | 0.81 | 0.48 |
| fac bacterial diversity | native pollen diversity*Temp | 3 | 0.39 | 0.72 |
| fac bacterial diversity | native pollen diversity*Rain | 3 | -0.42 | 0.71 |
| fac bacterial diversity | native pollen diversity*Wind | 3 | 1.26 | 0.30 |
| fac bacterial diversity | native pollen diversity*log_pasture | 3 | 1.62 | 0.20 |
| fac bacterial diversity | ‘both’ pollen diversity*Temp | 3 | -1.52 | 0.23 |
| fac bacterial diversity | ‘both’ pollen diversity*Rain | 3 | 0.66 | 0.56 |
| fac bacterial diversity | ‘both’ pollen diversity*Wind | 3 | 0.03 | 0.97 |
| fac bacterial diversity | ‘both’ pollen diversity*log_pasture | 3 | -1.43 | 0.25 |
| fac bacterial diversity | introduced pollen diversity*Temp | 3 | 0.99 | 0.40 |
| fac bacterial diversity | introduced pollen diversity*Rain | 3 | -0.73 | 0.52 |
| fac bacterial diversity | introduced pollen diversity*Wind | 3 | -1.23 | 0.31 |
| fac bacterial diversity | introduced pollen diversity*log_pasture | 3 | 0.87 | 0.45 |

**Table S17.** Tests used to evaluate the significance of the highest order interaction or main effect (if no interaction was present) involving *A. mellifera,* pollen (Chao1) richness, and/or environmental variables on gut bacterial (Chao1) richness. Statistically significant (p $\leq$ 0.05) interactions are shown in bold. Abbreviations: Temp = Mean annual temperature, Rain = Mean annual precipitation, Wind = Average summer wind velocity, log_pasture = logit transformed values for percentage of pasture, DF = Degrees of freedom, Fac bacterial richness = facultative bacterial richness. The asterisk (*) within predictor variables denotes a model with main effect and interaction.

| *Response variables* | *Predictor variables* | *DF* | *t* | *p* |
| --- | --- | --- | --- | --- |
| overall bacterial richness | overall pollen richness | 5 | 1.4 | 0.22 |
| overall bacterial richness | native pollen richness | 5 | 0.09 | 0.93 |
| overall bacterial richness | ‘both’ pollen richness | 5 | -0.15 | 0.88 |
| overall bacterial richness | introduced pollen richness | 5 | 1.4 | 0.22 |
| core bacterial richness | overall pollen richness | 5 | -1.09 | 0.33 |
| core bacterial richness | native pollen richness | 5 | -1.61 | 0.17 |
| core bacterial richness | ‘both’ pollen richness | 5 | -0.46 | 0.66 |
| core bacterial richness | introduced pollen richness | 5 | -0.19 | 0.85 |
| **fac bacterial richness** | **overall pollen richness** | **5** | **3.56** | **0.02** |
| fac bacterial richness | native pollen richness | 5 | 0.99 | 0.36 |
| fac bacterial richness | ‘both’ pollen richness | 5 | -0.61 | 0.57 |
| **fac bacterial richness** | **introduced pollen richness** | **5** | **3.06** | **0.03** |
| overall bacterial richness | Temp | 9 | -0.19 | 0.86 |
| overall bacterial richness | Rain | 9 | -1.19 | 0.27 |
| overall bacterial richness | Wind | 9 | -1.49 | 0.17 |
| overall bacterial richness | log_pasture | 9 | 1.07 | 0.31 |
| core bacterial richness | Temp | 9 | 0.1 | 0.92 |
| core bacterial richness | Rain | 9 | 1.57 | 0.15 |
| core bacterial richness | Wind | 9 | 1.53 | 0.16 |
| core bacterial richness | log_pasture | 9 | -0.05 | 0.96 |
| fac bacterial richness | Temp | 9 | 0.61 | 0.56 |
| fac bacterial richness | Rain | 9 | -1.24 | 0.25 |
| fac bacterial richness | Wind | 9 | -1.17 | 0.27 |
| fac bacterial richness | log_pasture | 9 | 1.15 | 0.28 |
| overall bacterial richness | Rain*Temp | 7 | -0.48 | 0.65 |
| **overall bacterial richness** | **Rain*log_pasture** | **7** | **2.71** | **0.03** |
| overall bacterial richness | Rain*Wind | 7 | -1.48 | 0.18 |
| overall bacterial richness | Temp*log_pasture | 7 | 1.17 | 0.28 |
| overall bacterial richness | Wind* Temp | 7 | 0.42 | 0.69 |
| overall bacterial richness | Wind*log_pasture | 7 | 0.74 | 0.48 |
| core bacterial richness | Rain*Temp | 7 | -0.37 | 0.72 |
| core bacterial richness | Rain*log_pasture | 7 | 0.36 | 0.73 |
| core bacterial richness | Rain*Wind | 7 | -0.83 | 0.44 |
| core bacterial richness | Temp*log_pasture | 7 | -0.18 | 0.86 |
| core bacterial richness | Wind* Temp | 7 | -1.77 | 0.12 |
| core bacterial richness | Wind*log_pasture | 7 | 0.95 | 0.37 |
| fac bacterial richness | Rain*Temp | 7 | -0.44 | 0.68 |
| **fac bacterial richness** | **Rain*log_pasture** | **7** | **3.08** | **0.02** |
| fac bacterial richness | Rain*Wind | 7 | -1.18 | 0.28 |
| fac bacterial richness | Temp*log_pasture | 7 | 0.72 | 0.49 |
| fac bacterial richness | Wind* Temp | 7 | 0.17 | 0.87 |
| fac bacterial richness | Wind*log_pasture | 7 | 0.71 | 0.5 |
| overall bacterial richness | overall pollen richness*Temp | 3 | 0.28 | 0.8 |
| overall bacterial richness | overall pollen richness*Rain | 3 | 1.72 | 0.18 |
| overall bacterial richness | overall pollen richness*Wind | 3 | 0.51 | 0.64 |
| overall bacterial richness | overall pollen richness*log_pasture | 3 | 0.74 | 0.51 |
| overall bacterial richness | native pollen richness*Temp | 3 | 0.43 | 0.7 |
| overall bacterial richness | native pollen richness*Rain | 3 | -2.74 | 0.07 |
| overall bacterial richness | native pollen richness*Wind | 3 | -0.96 | 0.41 |
| overall bacterial richness | native pollen richness*log_pasture | 3 | 0.99 | 0.39 |
| overall bacterial richness | ‘both’ pollen richness*Temp | 3 | -1.04 | 0.37 |
| overall bacterial richness | ‘both’ pollen richness*Rain | 3 | -0.5 | 0.65 |
| overall bacterial richness | ‘both’ pollen richness*Wind | 3 | 0.11 | 0.92 |
| overall bacterial richness | ‘both’pollen richness*log_pasture | 3 | -0.92 | 0.43 |
| overall bacterial richness | introduced pollen richness*Temp | 3 | 1.1 | 0.35 |
| overall bacterial richness | introduced pollen richness*Rain | 3 | 1.07 | 0.36 |
| overall bacterial richness | introduced pollen richness*Wind | 3 | 0.13 | 0.9 |
| overall bacterial richness | introduced pollen richness*log_pasture | 3 | 1.65 | 0.2 |
| core bacterial richness | overall pollen richness*Temp | 3 | 1.34 | 0.27 |
| core bacterial richness | overall pollen richness*Rain | 3 | 0.16 | 0.89 |
| core bacterial richness | overall pollen richness*Wind | 3 | 1.45 | 0.24 |
| core bacterial richness | overall pollen richness*log_pasture | 3 | -0.23 | 0.83 |
| core bacterial richness | native pollen richness*Temp | 3 | 1.72 | 0.18 |
| core bacterial richness | native pollen richness*Rain | 3 | -0.74 | 0.51 |
| core bacterial richness | native pollen richness*Wind | 3 | 0.59 | 0.6 |
| core bacterial richness | native pollen richness*log_pasture | 3 | 0.02 | 0.98 |
| core bacterial richness | 'both' pollen richness*Temp | 3 | 0.93 | 0.42 |
| core bacterial richness | 'both' pollen richness*Rain | 3 | 1.64 | 0.2 |
| core bacterial richness | 'both' pollen richness*Wind | 3 | 0.24 | 0.82 |
| core bacterial richness | 'both' pollen richness*log_pasture | 3 | 0.89 | 0.44 |
| core bacterial richness | introduced pollen richness*Temp | 3 | -0.22 | 0.84 |
| core bacterial richness | introduced pollen richness*Rain | 3 | -0.38 | 0.73 |
| core bacterial richness | introduced pollen richness*Wind | 3 | 2.25 | 0.11 |
| core bacterial richness | introduced pollen richness*log_pasture | 3 | -0.74 | 0.51 |
| fac bacterial richness | overall pollen richness*Temp | 3 | 0.69 | 0.54 |
| fac bacterial richness | overall pollen richness*Rain | 3 | 1.79 | 0.17 |
| fac bacterial richness | overall pollen richness*Wind | 3 | 1.02 | 0.38 |
| fac bacterial richness | overall pollen richness*log_pasture | 3 | 0.96 | 0.41 |
| fac bacterial richness | native pollen richness*Temp | 3 | 0.54 | 0.63 |
| fac bacterial richness | native pollen richness*Rain | 3 | -3.05 | 0.06 |
| fac bacterial richness | native pollen richness*Wind | 3 | -0.81 | 0.48 |
| fac bacterial richness | native pollen richness*log_pasture | 3 | 1.7 | 0.19 |
| fac bacterial richness | ‘both’ pollen richness*Temp | 3 | -1.85 | 0.16 |
| fac bacterial richness | ‘both’ pollen richness*Rain | 3 | 0.23 | 0.84 |
| fac bacterial richness | ‘both’ pollen richness*Wind | 3 | -0.07 | 0.95 |
| fac bacterial richness | ‘both’ pollen richness*log_pasture | 3 | -0.67 | 0.55 |
| fac bacterial richness | introduced pollen richness*Temp | 3 | 1.87 | 0.16 |
| fac bacterial richness | introduced pollen richness*Rain | 3 | -0.24 | 0.83 |
| fac bacterial richness | introduced pollen richness*Wind | 3 | -0.46 | 0.68 |
| fac bacterial richness | introduced pollen richness*log_pasture | 3 | 2.64 | 0.08 |

**Table S18.** Tests to assess linear relationships between alpha diversity (Shannon’s diversity and Chao1 richness) of pollen foraged by *B. terrestris* and environmental factors. Statistically significant (p $\leq$ 0.05) relationships are shown in bold. Abbreviations: Temp = Mean annual temperature, Rain = Mean annual precipitation, Wind = Average summer wind velocity, Pasture = Percentage of pasture.

| *Response variables* | *Predictor variables* | *p* | *r^2^* |
| --- | --- | --- | --- |
| overall pollen diversity | Rain | 0.77 | -0.10 |
| native pollen diversity | Rain | 0.73 | -0.09 |
| ‘both’ pollen diversity | Rain | 0.69 | -0.09 |
| introduced pollen diversity | Rain | 0.53 | -0.06 |
| overall pollen diversity | Temp | 0.23 | 0.06 |
| native pollen diversity | Temp | 0.24 | 0.05 |
| ‘both’ pollen diversity | Temp | -0.11 | 0.87 |
| introduced pollen diversity | Temp | 0.35 | -0.0008 |
| **overall pollen diversity** | **Wind** | **0.05** | **0.30** |
| native pollen diversity | Wind | 0.24 | 0.05 |
| ‘both’ pollen diversity | Wind | 0.56 | -0.06 |
| introduced pollen diversity | Wind | 0.27 | 0.04 |
| overall pollen diversity | Pasture | 0.27 | 0.04 |
| native pollen diversity | Pasture | 0.84 | -0.11 |
| ‘both’ pollen diversity | Pasture | 0.06 | 0.25 |
| introduced pollen diversity | Pasture | 0.68 | -0.09 |
| overall pollen richness | Rain | 0.70 | -0.09 |
| native pollen richness | Rain | 0.96 | -0.11 |
| ‘both’ pollen richness | Rain | 0.66 | -0.08 |
| introduced pollen richness | Rain | 0.93 | -0.11 |
| overall pollen richness | Temp | 0.33 | 0.008 |
| native pollen richness | Temp | 0.45 | -0.04 |
| ‘both’ pollen richness | Temp | 0.49 | -0.05 |
| introduced pollen richness | Temp | 0.34 | 0.003 |
| overall pollen richness | Wind | 0.54 | -0.06 |
| native pollen richness | Wind | 0.36 | -0.005 |
| ‘both’ pollen richness | Wind | 0.92 | -0.11 |
| introduced pollen richness | Wind | 0.11 | 0.18 |
| overall pollen richness | Pasture | 0.36 | -0.005 |
| native pollen richness | Pasture | 0.35 | -0.003 |
| ‘both’ pollen richness | Pasture | 0.99 | -0.11 |
| introduced pollen richness | Pasture | 0.81 | -0.10 |

**Table S19.** Percentage of different plant types foraged by *A. mellifera* across Tasmania. ‘Native’ indicates native (including endemic) plant genera in Australia; ‘introduced’ indicates plant genera that have been introduced or naturalised in Tasmania; ‘both’ indicates plant genera containing both native and introduced species in Tasmania.

| Sites | Native pollen | Introduced pollen | ‘Both’ pollen |
| --- | --- | --- | --- |
| T5 | 0.03 | 65.87 | 34.10 |
| T9 | 44.84 | 6.96 | 48.20 |
| T10 | 25.44 | 69.55 | 5.01 |
| T18 | 33.27 | 54.09 | 12.63 |
| T22 | 2.02 | 28.21 | 69.77 |
| T30 | 19.36 | 78.30 | 2.33 |
| T32 | 15.54 | 38.39 | 46.07 |
| Average | 20.07 | 48.77 | 31.16 |
| Standard error | 6.11 | 9.64 | 9.60 |

**Table S20.** Percentage of different plant types foraged by *B. terrestris* across Tasmania. ‘Native’ indicates native (including endemic) plant genera in Australia; ‘introduced’ indicates plant genera that have been introduced or naturalised in Tasmania; ‘both’ indicates plant genera containing both native and introduced species in Tasmania.

| Sites | Native pollen | Introduced pollen | ‘Both’ pollen |
| --- | --- | --- | --- |
| T1 | 0.33 | 70.75 | 28.93 |
| T5 | 1.09 | 3.00 | 95.91 |
| T6 | 88.24 | 11.69 | 0.07 |
| T9 | 88.22 | 11.68 | 0.10 |
| T10 | 0.71 | 84.50 | 14.79 |
| T21 | 0.56 | 65.51 | 33.92 |
| T22 | 95.43 | 0.42 | 4.14 |
| T23 | 9.16 | 90.70 | 0.14 |
| T25 | 5.85 | 94.15 | 0.00 |
| T30 | 4.62 | 95.22 | 0.16 |
| T32 | 14.15 | 83.27 | 2.58 |
| T33 | 18.38 | 79.00 | 2.62 |
| Average | 27.23 | 57.49 | 15.28 |
| Standard error | 11.17 | 11.14 | 8.09 |

**Table S21.** Tests to assess linear relationships between alpha diversity (Shannon’s diversity and Chao1 richness) of pollen foraged by *A. mellifera* and environmental factors. Statistically significant (p $\leq$ 0.05) relationships are shown in bold. Abbreviations: Temp = Mean annual temperature, Rain = Mean annual precipitation, Wind = Average summer wind velocity, Pasture = Percentage of pasture.

| *Response variables* | *Predictor variables* | *p* | *r^2^* |
| --- | --- | --- | --- |
| overall pollen diversity | Rain | 0.12 | 0.30 |
| native pollen diversity | Rain | 0.41 | -0.03 |
| ‘both’ pollen diversity | Rain | 0.83 | -0.19 |
| introduced pollen diversity | Rain | 0.54 | -0.10 |
| overall pollen diversity | Temp | 0.31 | 0.04 |
| native pollen diversity | Temp | 0.34 | 0.02 |
| ‘both’ pollen diversity | Temp | 0.78 | -0.18 |
| introduced pollen diversity | Temp | 0.62 | -0.14 |
| overall pollen diversity | Wind | 0.78 | -0.18 |
| native pollen diversity | Wind | 0.76 | -0.18 |
| ‘both’ pollen diversity | Wind | 0.19 | 0.18 |
| introduced pollen diversity | Wind | 0.43 | -0.05 |
| overall pollen diversity | Pasture | 0.56 | -0.12 |
| native pollen diversity | Pasture | 0.46 | -0.06 |
| ‘both’ pollen diversity | Pasture | 0.17 | 0.22 |
| introduced pollen diversity | Pasture | 0.56 | -0.11 |
| **overall pollen richness** | **Rain** | **0.009** | **0.73** |
| native pollen richness | Rain | 0.35 | 0.01 |
| ‘both’ pollen richness | Rain | 0.60 | -0.13 |
| introduced pollen richness | Rain | 0.28 | 0.07 |
| overall pollen richness | Temp | 0.31 | 0.05 |
| native pollen richness | Temp | 0.26 | 0.09 |
| ‘both’ pollen richness | Temp | 0.87 | -0.19 |
| introduced pollen richness | Temp | 0.58 | -0.12 |
| overall pollen richness | Wind | 0.41 | -0.04 |
| native pollen richness | Wind | 0.59 | -0.13 |
| ‘both’pollen richness | Wind | 0.39 | -0.02 |
| introduced pollen richness | Wind | 0.25 | 0.11 |
| overall pollen richness | Pasture | 0.33 | 0.02 |
| native pollen richness | Pasture | 0.57 | -0.12 |
| ‘both’ pollen richness | Pasture | 0.31 | 0.04 |
| introduced pollen richness | Pasture | 0.13 | 0.28 |

**SUPPLEMENTARY FIGURES**

**
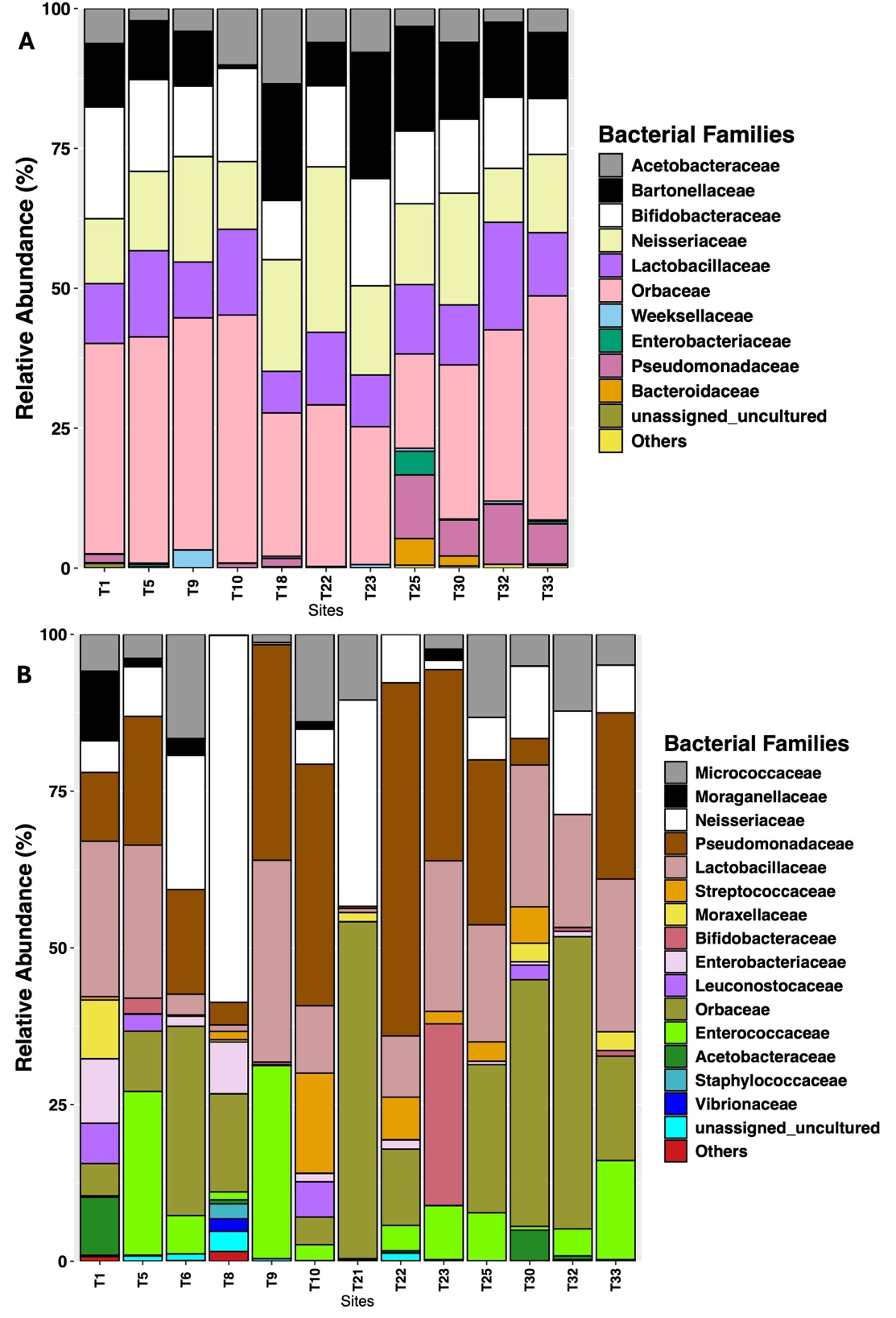
**

**Figure S1.** Key bacterial families found in the guts of (A) *A. mellifera* and (B) *B. terrestris* across Tasmania. In both plots, ‘Others’ represents sum of all bacterial families with relative abundance of less than 1%, while ‘unassigned_uncultured’ refers to bacteria that could not be classified into any specific family.


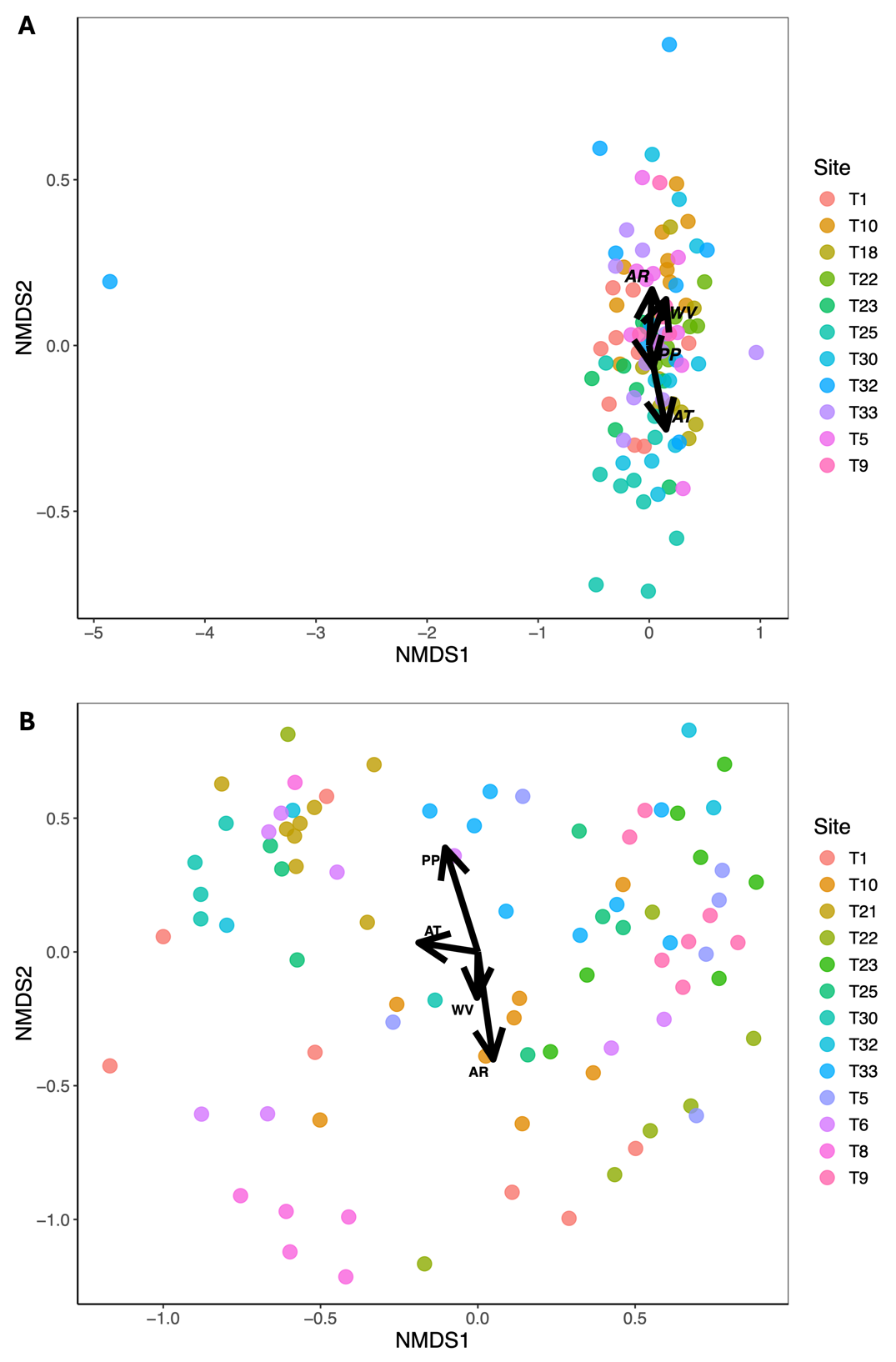


**Figure S2.** NMDS ordination of gut bacterial communities for (A) *A. mellifera* and (B) *B. terrestris* based on Bray-Curtis dissimilarity of ASV abundance of individual samples. Stress for both NMDS plots = 0.2. Abbreviations: AT = Mean annual temperature (°C), AR = Mean annual precipitation (mm), PP = Percentage of pasture (%), WV = Average summer wind velocity (m/s). Refer to Table S6 for summary of environmental vector correlations (envfit; 999 permutations).


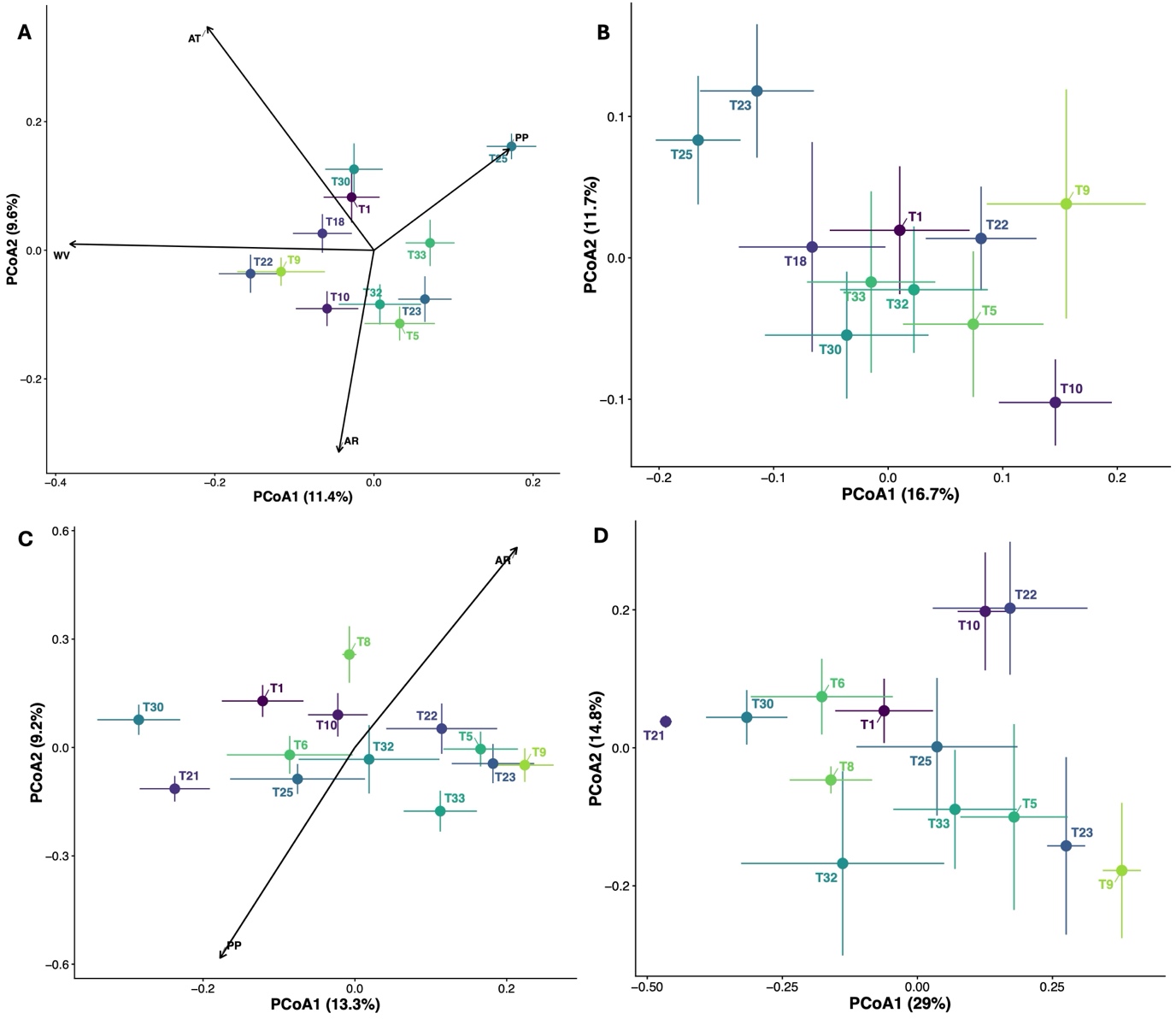


**Figure S3.** PCoA ordinations of gut microbiome composition based on Jaccard (A, C) and Bray–Curtis (B, D) dissimilarities for A. mellifera (A, B) and B. terrestris (C, D). Percent variance explained by the first two axes is shown on each axis. Abbreviations: AT = Mean annual temperature (°C), AR = Mean annual precipitation (mm), PP = Percentage of pasture (%), WV = Average summer wind velocity (m/s). Refer to Table S7 for summary of environmental vector correlations (envfit; 999 permutations).


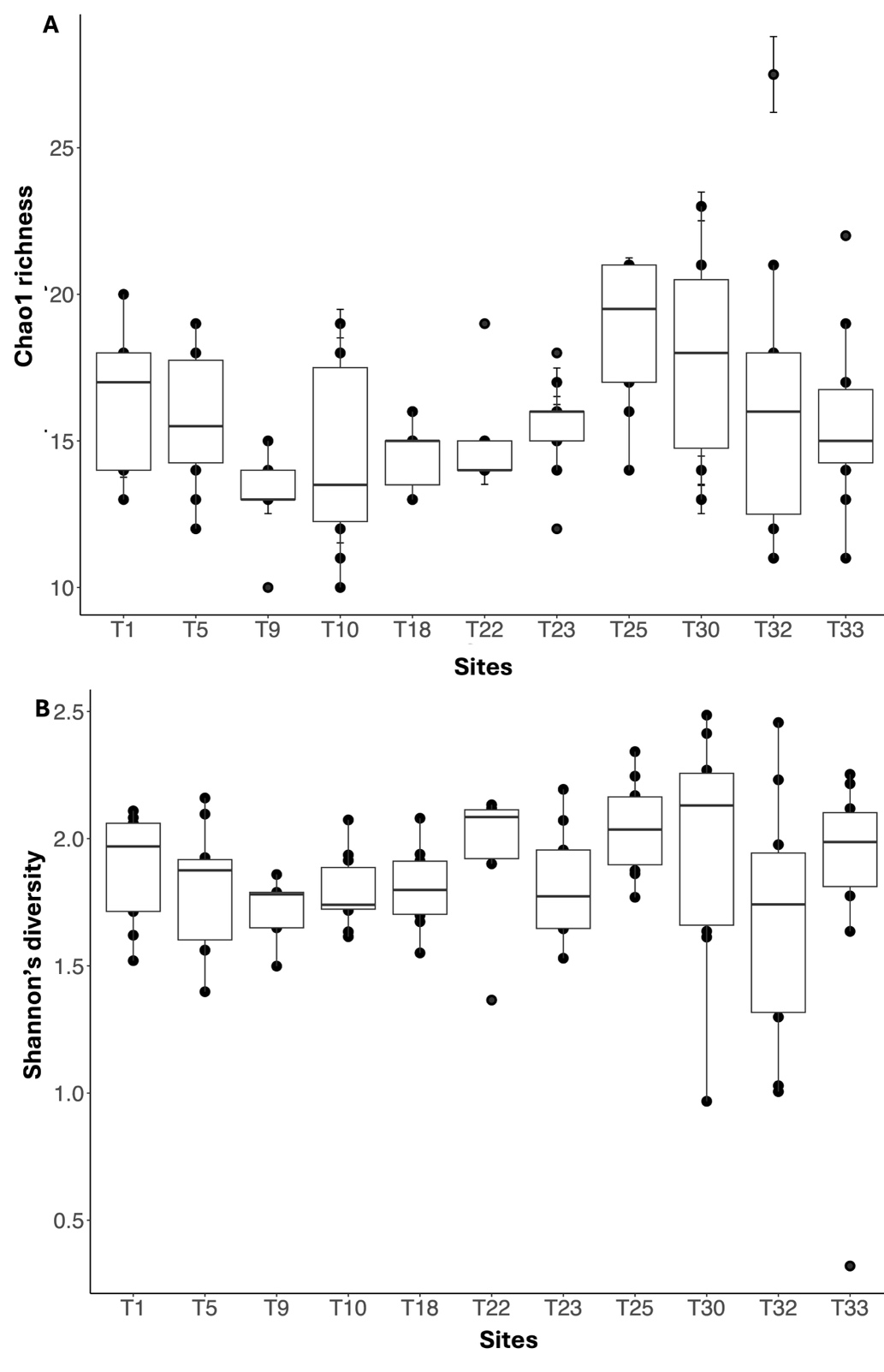


**Figure S4.** Alpha diversity of *A. mellifera* gut microbiomes across Tasmania. (A) Chao1 richness of *A. mellifera* per site. All sites showed statistical significance (ANOVA: p = 0.01) and pairwise site comparisons revealed T9 significantly differed from T25 (Tukey: p = 0.03). (B) Shannon’s diversity of *A. mellifera* per site*.* All sites were statistically insignificant (ANOVA: all p > 0.05). Refer to Table S5 for all corresponding ANOVA and Tukey post-hoc results for *A. mellifera* alpha diversity.

**
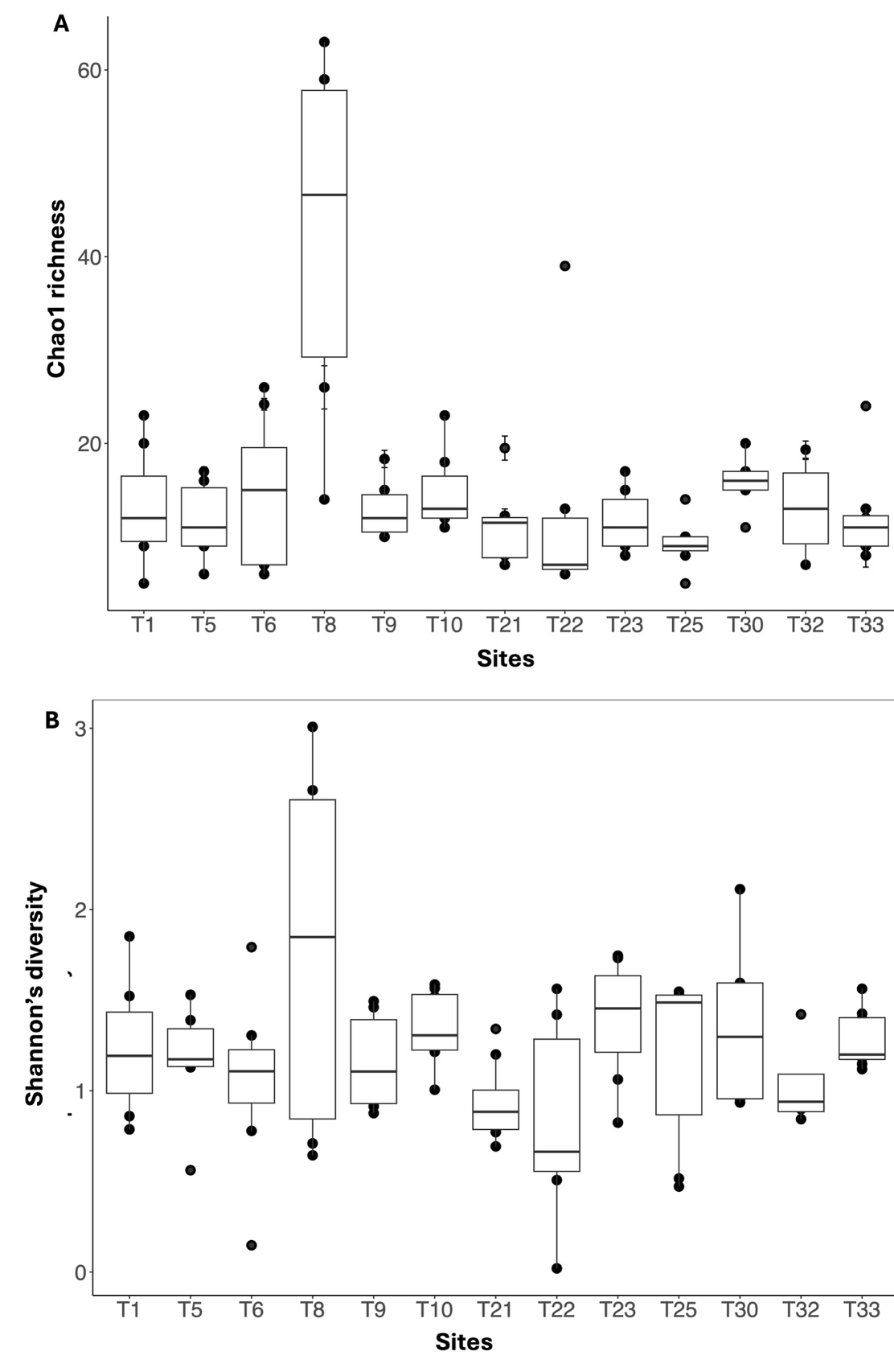
**

**Figure S5.** Alpha diversity of *B. terrestris* bee gut microbiomes across Tasmania. (A) Chao1 richness of *B. terrestris* per site. All sites showed statistical significance (ANOVA: p = 4.75e^-9^) with T8 significantly differing from all other sites (Tukey: p < 0.001) (B) Shannon’s diversity of *B. terrestris* per site. All sites showed marginal significance (ANOVA: p = 0.05) with T8 differing from T21 (Tukey: p = 0.04) and T22 (Tukey: p = 0.02). Refer to Table S6 for all corresponding ANOVA and Tukey post-hoc results for *B. terrestris* alpha diversity.

**Figure S6.** Positive relationships between mean annual precipitation and (A) facultative gut bacterial diversity, (B) overall gut bacterial diversity, (C) facultative gut bacterial richness, (D) overall gut bacterial richness of *B. terrestris* across Tasmania. Measure of diversity = Shannon; Measure of richness = Chao1.

**

**Figure S7.** Interaction effect of pasture x precipitation on (A) overall gut bacterial richness of *B. terrestris.* (B) overall gut bacterial richness of *A. mellifera* and (C) facultative gut bacterial richness of *A. mellifera.* Richness measure = Chao1.

**Figure S8.** The negative correlation between average summer wind velocity and (A) core gut bacterial diversity of *B. terrestris.* (B) overall diversity of pollen foraged by *B. terrestris.* Diversity measure = Shannon.


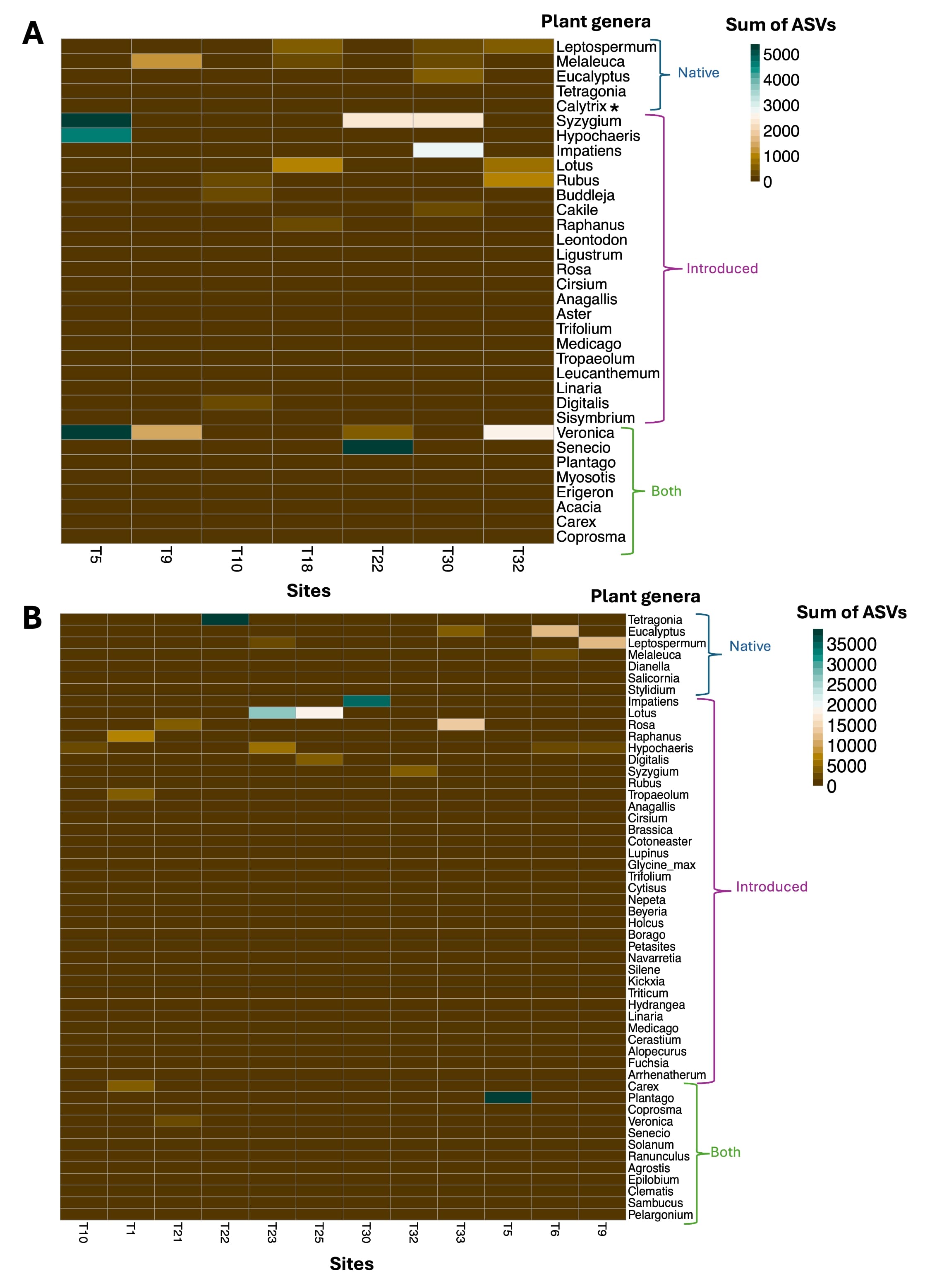


**Figure S9.** Heatmap displaying all plants identified from the pollen baskets of (A) *A. mellifera* and (B) *B. terrestris*. In both plots, the plant genera are categorized as native, introduced, or ‘both’. The colour scales indicate the sum of ASVs of different plant genera per site. *Calytrix is an endemic plant genus in Australia.

**Figure S10.** Species variation in response of the interaction between native pollen diversity and mean annual temperature on (A) facultative and (B) overall gut bacterial diversity. In both plots, circles represent *Apis* sites and triangles represent *Bombus* sites; site abbreviations (A=*Apis* and B=*Bombus*) are included alongside site names. Diversity measure = Shannon.

**

**Figure S11.** Positive correlations between facultative gut bacterial richness and (A) introduced pollen richness, (B) overall pollen richness of *A. mellifera* across Tasmania. Richness measure = Chao1.

**Figure S12.** The negative relationship between mean annual precipitation and overall richness of pollen foraged by *A. mellifera.*
